# Neurobiology of co-morbid stress and a western diet in mice: mitochondrial, proteomic and behavioral outcomes

**DOI:** 10.1007/s11011-026-01855-3

**Published:** 2026-04-27

**Authors:** Tessa Helman, Makayla Nicholas, Brock Lyon, Saba Naghipour, Chul-Kyu Kim, Trissha Ybanez, Kai Robertson, Tia A. Griffith, Jason N. Peart, Nicolas J. C. Stapelberg, John P. Headrick, Eugene F. Du Toit

**Affiliations:** 1 Centre for Healthy Brain Ageing (CHeBA), Discipline of Psychiatry and Mental Health, School of Clinical Medicine, UNSW, Sydney, Australia; 2 School of Pharmacy and Medical Sciences, Griffith University, Southport, Gold Coast, QLD 4217 Australia; 3 O’Brien Institute, St Vincent’s Institute of Medical Research, Fitzroy, Australia; 4 Bond University, Robina, Australia

**Keywords:** Anxiety, Co-morbidity, Depression, Mitochondria, Mouse, Respiration, Stress, Synaptogenesis, Western diet

## Abstract

Chronic stress (CS) and hyper-caloric diets promote affective disorders, however pathobiological interactions await definition. Neurobiological and behavioral influences of chronic stress (CS; 2 h/day restraint for 2 wks) were studied in male C57Bl/6 mice fed a control diet (CD; 12% kcal as fat/65% carbohydrate/23% protein) or Western diet (WD; 32% kcal fat/57% carbohydrate/11% protein) for 20 wks. CS induced weight loss and anxiety-like behavior, associated with reduced frontal cortex (FC) brain-derived neurotrophic factor (BDNF) and γ-aminobutyric acid (GABA) *vs*. elevated glutamate; and mitochondrial dysfunction, including reduced complex I (CI), electron transport system (ETS) and spare respiratory capacities. Hippocampus (HPC) appeared less responsive (CI flux selectively inhibited). Proteomics supports CS disruption of synaptogenesis and mitochondrial pathways, together with “adaptive” changes, including activation of neuroprotective chaperone-mediated autophagy (CMA), reelin and eukaryotic initiation factor 2 (EIF2) paths. The WD alone (inducing weight gain, insulin-resistance and reduced sucrose preference) had no independent effects on FC neurochemistry, FC or HPC respiratory function. Co-morbid CS in WD mice induced anxiety-like behavior and anhedonia, coupled with WD-related reductions in FC GABA and HPC BDNF not evident in un-stressed mice. A co-morbid WD also limited CS-dependent changes in ETS and spare respiratory capacities. Proteomics supports reduced CS activation of reelin and EIF2 pathways, and a switch from CMA activation to suppression, in co-morbid CS + WD mice. *Summary*: Mild anxiogenic stress disrupts mitochondrial function, glutamate:GABA balance and BDNF/synaptogenesis signaling, primarily in FC *vs.* HPC. Co-morbid CS + WD exaggerates neurochemical disturbances, and disrupts adaptive pathway responses, in association with anhedonia and anxiety-like behaviors.

**Supplementary Information:**

The online version contains supplementary material available at 10.1007/s11011-026-01855-3.

## Introduction

Chronic psychosocial stress and a Western type diet are important risk factors for mood and metabolic disorders. Psychological stress, diet and obesity are strongly inter-related, owing in part to shared neurocircuitry regulating stress responses and food intake (Masniam and Morris 2012), and shared mechanistic pathways as outlined for example in the psycho-immune-neuroendocrine (PINE) network and other integrative models (Stapelberg et al. 2015, 2018). These prominent co-morbidities may drive both adaptive and maladaptive changes in neurobiology and behavior. However, the interactions between an unhealthy diet and CS remain to be detailed. Indeed, experimental findings appear contradictory, with the influences of palatable high fat or carbohydrate diets ranging from protection against stress-related abnormalities (Finger et al 2011; Maniam et al. 2016), to a worsening of outcomes (Santos et al. 2018). We recently reported additive effects of a WD and mild stress on behavioral and metabolic disruption (Du Toit et al. 2020), and increased vulnerability to a WD in mice subjected to early life stress (Robertson et al. 2024). On the other hand, we also found stress in adult mice may counter WD-dependent changes in select cardiometabolic risk factors (Hatton-Jones et al. 2022). Interactions between high fat/carbohydrate diets and chronic stressors thus appear complex and variable, reflecting in part our incomplete understanding of underlying mechanisms.

As we have outlined previously, the biological networks underlying stress-related disorders may be subject to critical transitions, including inhibition or reversal of beneficial feedback loops (Stapelberg et al. 2018). Thus, co-morbid stressors may not simply additively disrupt neurobiological function, but inhibit/reverse intrinsic neuroprotective or adaptive responses. Such changes may underpin transition from a pre-disease to disease state (Stapelberg et al. 2018, 2019). While we have presented evidence of a highly integrated PINE network in stress-related disorders (Helman et al. 2022a, b), whether these predicted regulatory transitions arise awaits investigation. We here characterize the independent and combined neurobiological effects of CS and a Western-type diet with moderately elevated fat (32% of calories), and employ exploratory proteomics to test whether co-morbid CS and a WD may exacerbate behavioral and neurobiological dysfunction in association with maladaptive transitions in feedback mechanisms influencing stress resilience or vulnerability.

## Methods

### Animal ethics

All animal studies were performed in accordance with the ARRIVE 2.0 guidelines and “The Animal Care and Protection Act 2001 (QLD), and were approved by the Animal Ethics Committee of Griffith University in accordance with these policy guidelines Male C57Bl/6 mice were supplied by the Animal Resource Centre (Perth, Australia) and housed in the Griffith University Animal Facility for the duration of the study (ethics approval number MSC/02/20/AEC). This initial study assessed male mice, avoiding potential confounding effects of estrous cycle-related hormonal fluctuations in females (influencing behavioral and neurobiological outcomes; and increased variability requiring substantially larger sample sizes to preserve statistical power).

### Experimental models of stress and WD feeding

Male C57Bl/6 mice (8 wks of age) were randomly allocated to cages housing 4 mice each on arrival in the animal facility (total *n* = 64 mice; in 16 cages), and provided with ad libitum access to water and food. Mice were habituated to the facility for at least 1 wk prior to initiation of studies and maintained in a 12-h day/night lighting cycle at 21 °C and 40% humidity. Each cage was then randomly allocated by a different investigator (guided by computer-generated random sequences) to 4 experimental groups: CD, WD, CD + CS or WD + CS. A detailed experimental timeline is provided in the Supplement file. To test influences of diet, mice were subjected to 20 wks of either WD (*n* = 32; diet composition included 32% kcal as fat, 57% carbohydrate, 11% protein) or standard CD chow (*n* = 32; 12% kcal as fat, 65% carbohydrate, 23% protein; Specialty Feeds, WA, Australia) (Du Toit et al. 2020; Hatton-Jones et al. 2022). Throughout the final 2 experimental weeks, mice were either subjected to a mild CS protocol involving a daily 2-h period of restraint (between 0900 and 1100 h) in a plexiglass tube (Helman et al. 2022a, b; Park et al. 2014) or were handled for 2 min and returned to their cages (“un-stressed” mice). We calculated minimum sample sizes to detect significant differences in behavior, neurochemistry and respiratory function (80% power, *α* = 0.05), using experimental data (and variances) acquired from the same model; indicating *n* = 12–16 for different behavioral outcomes, and *n* = 8 for neurochemical and mitochondrial outcomes, respectively. Practical constraints limited our ability to assess neurochemistry and mitochondrial function in every single animal, thus these latter analyses were undertaken on every 2nd mouse assessed (*n* = 8 providing sufficient statistical power). All behavioral, metabolic, neurochemical, mitochondrial and proteomic analyses were undertaken by investigators blinded to animal identity and experimental groupings. Final statistical analysis of these different data-sets was not performed in a blinded manner.

### Behavioral analyses

Behavior was assessed via open field (OFT) and sucrose preference (SPT) tests as detailed previously (Griffith et al. 2022; Hatton-Jones et al. 2021; Helman et al. 2022a, b). While not possible to model all aspects of major depressive disorder (MDD) in animals, these low stress tests permit assessment of 2 key elements—anhedonia and anxiety-like behavior—while limiting the major stress and neurobiological disturbances associated with added tests of learned helplessness or behavioral despair (with the latter also of questionable validity; Gencturk and Unal 2024). Our approach is consistent with the “3 Rs” ethical framework, reducing animal numbers and duration of experimentation (whereas use of learned helplessness or behavioral despair tests necessitates more protracted experimentation to permit neurological recovery post-test, or use of additional animal groups to assess behavior and neurobiology).

Briefly, the OFT was conducted within a 70 cm × 70 cm arena with a 4 × 4 marked grid and a center square. Animals commenced the test in the center square and were recorded for 30 min via digital camera. Testing was conducted between 0800–1400 h to limit circadian influences, under moderate light conditions (125 Lux). Open field behavior was analyzed using the EthoVision XT program, including line crossings, distance, rearing, immobility and center square entries.

The SPT estimates hedonic behavior (Gencturk and Unal 2024), testing preference for a 1% sucrose solution over water. Mice were initially placed in a cage with 2 bottles containing water over a 24 h acclimation period. Bottles were then refilled, one with water and one with 1% sucrose for a further 24 h period during which consumption was measured. Sucrose preference (%) was calculated as:

$$\left(\frac{Weight of 1\% sucrose solution \left(g\right)}{Weight of 1\% sucrose solution \left(g\right)+weight of water \left(g\right)}\right)\text{ x} 100\%$$

### Tissue collection

At 24 h after the SPT, mice were anesthetized with 60 mg/kg (I.P) sodium pentobarbital. Blood was collected into uncoated tubes and rested on ice for 60 min prior to 10 min centrifugation at 1000* g* (4 °C). Brains were rapidly removed into ice-cold phosphate-buffered saline and dissected under microcopy into FC, HPC, hypothalamus and nucleus accumbens from left and right hemispheres. Right hemisphere tissue was frozen in liquid N_2_ for protein analysis, while left hemisphere tissue was immediately used for mitochondrial respiratory analysis (Sect. "Mitochondrial analyses"), as recently detailed for cardiac tissue (Naghipour et al. 2023). Hypothalamus and nucleus accumbens were also analyzed (see Supplement file).

### Metabolic and endocrine profiles

Blood was sampled via tail bleed in fasted animals for analysis of glucose (Accu-Check II glucometer; Roche Diagnostics, Castle Hill, Australia), insulin (ELISA; #90080, Crystal Chem, Downer Grove, Illinois, USA), triglycerides (Triglyceride Quantification Colorimetric/Fluorometric Kit, BioVision, California, USA) and cholesterol (QuickDetect™ Total cholesterol Mouse ELISA Kit, Biovision, California, USA). Non-fasted blood was analyzed for leptin and ghrelin (Mouse Leptin LEP and Mouse ghrelin GHRL ELISA Kits, Cusabio, Houston, Texas), norepinephrine (Norepinephrine ELISA Kit #KA3836, Abnova, Taipei, Taiwan) and melatonin (Melatonin ELISA Kit #E4630, Milpitas, California). Insulin sensitivity was estimated using the homeostatic model assessment for insulin-resistance (HOMA-IR) (Du Toit et al. 2020; Griffith et al. 2022), a validated measure in mice (Lee et al. 2008):

$$\mathrm{HOMA}-\mathrm{IR}=\left[\text{fasting insulin }\left(\mathrm{ng}/\mathrm{mL}\right)\times \text{fasting glucose }\left(\mathrm{mg}/\mathrm{dL}\right)\right]/405.$$

### Mitochondrial analyses

Mitochondrial function was monitored in a high-resolution Oxygraph-O2k system (Oroboros, Innsbruck, Austria) at 37 °C, using the Substrate-Uncoupler-Inhibitor-Titration protocol (Pesta and Gnaiger 2012). Chambers were calibrated with 2.2 mL Mir05 respiration media (0.5 mM EGTA, 3 mM MgCl_2_·6H_2_O, 60 mM K-lactobionate, 20 mM taurine, 10 mM KH_2_PO_4_, 20 mM HEPES, 110 mM sucrose, 1 g/L fatty acid-free bovine serum albumin, pH 7.1) until stable O_2_ flux was maintained. Fresh samples of FC, HPC, hypothalamus or nucleus accumbens were homogenized in cold Mir05 respiration media (1 mg tissue per mL) and a 2.2 mL sample loaded into each chamber. After 5 min baseline equilibration, 5 mM pyruvate, 2 mM malate and 10 mM glutamate were added before titration of ADP (1–5 mM) to assess CI, leak and maximum respiration, respectively. Cytochrome *c* (10 μM) was added to test outer mitochondrial membrane integrity, followed by 10 mM succinate for CI and CII linked maximum respiration. Titration with carbonyl cyanide m-chlorophenyl hydrazone (0.5–1.5 mM) permitted measurement of maximum respiratory capacity. Complex I and III inhibition was achieved with 1 µM rotenone and 5 mM antimycin A, respectively, allowing measurement of residual O_2_ consumption (ROX). Complex IV capacity was assessed by sequential addition of 2 mM ascorbate and 0.5 mM N, N, N′, N′-tetramethyl-p-phenylenediamine. Values for ROX were subtracted from fluxes to remove “non-mitochondrial” activity. Flux control ratios were calculated by normalizing fluxes to maximal respiratory capacity during uncoupling (see Table S1). To ensure optimum conditions for oxidative phosphorylation, chamber O_2_ concentrations were maintained above 30 µM by opening chambers to equilibrate gas and liquid phases until baseline concentrations of 180–200 µM were restored. Respiratory rates were recorded using OROBOROS DataLab 7.0 software, and units of respiration expressed as pmol O_2_/sec/mg.

### Protein extraction and analysis

Frozen FC and HPC samples were homogenized in chilled buffer (1:9 sample:buffer *v/v*; Bio-Rad Laboratories, USA) and centrifuged 10 min at 4500* g* (4 °C). Supernatant was aliquoted into 1.5 mL tubes and stored at −80 °C. Protein content was determined using a bicinchoninic acid assay (ThermoFisher, Illinois, USA), with absorbance measured on a FLUOstar plate reader (BMG LabTech, Victoria, Australia) at a wavelength of 540 nm.

#### ELISA analysis

Samples containing 45–80 µg protein were transferred to chilled tubes, and MilliQ Ultrapure Water (Merck-Millipore, Bedford, USA) added to normalize volumes. Samples were assayed via ELISA for GABA (BioVision, E4456-100), tryptophan (Immusmol, BA-E-2700), leptin (Cusabio, CSB-E04650m), and glutamate (Abnova, KA1909, BDNF (Abnova, KA0331), serotonin (Abnova KA2518), noradrenaline (Abnova, KA3836) and dopamine (Abnova, KA3838).

#### Discovery proteomics

Proteome-wide analyses permits un-biased exploration of potential pathway involvement in responses to stress (eg. Filipovic et al. 2024; Filipovic and Turck 2025). Here we apply proteomics to explore pathway changes with CS and/or a WD, and to test whether neuroprotective responses to stressors may be inhibited/absent, or in some cases reversed (eg. pathway activation with CS in CD mice *vs.* pathway inhibition with CS in WD mice) under co-morbid conditions (Stapelberg et al. 2018, 2019). Throughout the work we describe responses that are canonically neuroprotective or beneficial as ‘adaptive’ (neurobiological adjustments thought to limit/counteract the detrimental impacts of stress); and responses thought to promote dysfunction as ‘maladaptive’ (neurobiological changes thought to mediate/promote the impacts of stress). For example, activation of BDNF or neurogenesis signaling, autophagic quality control, mitochondrial biogenesis, NRF2 or IEF2 stress responses are considered ‘adaptive’; while inhibition of such paths (or the activation of detrimental processes, such as TP53 death signaling, ROS generation or mitochondrial membrane permeabilization/depolarization) is interpreted as ‘maladaptive’. Relevant examples are highlighted in Table 3.

Frozen brain tissue samples containing 50 µg protein were prepared for proteomic analysis via nano-LC MS/MS, as outlined in detail in the Supplement. Digested peptides were separated by nanoLC using an Ultimate3000 nano RSLC UPLC and an autosampler system (ThermoFisher, Illinois, USA). Peak lists were generated using Mascot Daemon together with Mascot Distiller and searched against an in-house Mascot database server (Matrix Science; v2.5.1) for identification/quality checking. Quantitative proteomic processing of raw files was performed using MaxQuant (v2.1.3.0) (Tyanova et al. 2016). Searches were performed against the UniProt mouse database (downloaded 29–08–2022) with a precursor mass tolerance of ± 4.0 ppm. Carbamidomethylation of cysteine was set as a fixed modification, while methionine oxidation and protein N-terminal acetylation were set as variable modifications. Differential protein abundance analysis was conducted in Perseus (v2.0.6.0) using label-free quantification (LFQ) values. For each comparison, log2-transformed LFQ intensities were compared between groups using two-sample t-tests, and proteins were considered differentially expressed based on both the t-test *p*-value (*p* ≤ 0.05) and a fold-change threshold (≥ 1.2 for up-regulation and ≤ 0.83 for down-regulation). Pathway enrichment analysis of differentially expressed proteins was performed using QIAGEN Ingenuity Pathway Analysis (IPA), and canonical pathway enrichment significance was assessed using Fisher’s exact test (*p* ≤ 0.05). Briefly, the gene identifiers and their fold-change for FC and HPC samples of each experimental group were separately uploaded. Experimental analyses included: a) impact of diet alone (CD *vs*. WD); b) impact of stress alone (CD *vs*. CS); and c) effect of stress in WD mice (WD *vs*. WD + CS). Significant interaction networks (*p* ≤ 0.05) and molecular and cellular functions were identified on known protein–protein interactions from the published literature. All relevant data are available in the paper and Data Supplement files. Complete proteomic datasets are available via ProteomeXchange with the identifier PXD074187.

### Statistical analysis

Statistical analysis was completed using GraphPad Prism version 8.1.1 (GraphPad Software, La Jolla, California, USA) and Microsoft Excel. Results are presented as mean ± SEM. Outlying data were identified for removal using ROUT method (*Q* = 1%), with normality of data determined via visual inspection of Q-Q plots. Homoscedasticity was confirmed for all measures using Levene’s Test. A 2-way ANOVA was used to test for main effects of either diet or stress, and any interaction, for every outcome measure. Where significant effects were identified, multiple comparisons were performed using the Holm-Sidak correction. For non-normally distributed data (assessed via Shapiro–Wilk test) a Kruskal–Wallis test was employed with Dunn’s test for pairwise comparisons—this was undertaken specifically for analysis of spare respiratory capacities for HPC, hypothalamus and nucleus accumbens. In all tests, effects were considered significant when *p* < 0.05. Effects sizes for significantly differing measures are shown as partial eta squared (η^2^_p_), indicating the variance in a dependent variable accounted for by independent variables (diet, stress or their interaction). Values range from 0 to 1, with ~ 0.01 reflecting a small effect, ~ 0.06 moderate and ≥ 0.14 a large effect. The 95% confidence intervals (CI_95_) and effect sizes (η^2^_p_) are provided for all significantly modified variables throughout the manuscript. Given the fundamental limitations of dichotomous null hypothesis testing, recommendations of the American Statistical Association regarding these drawbacks and the importance of effect sizes and confidence intervals (Slim et al. 2026; Szucs & Ioannidis 2017; Wasserstein & Lazar 2016; Wasserstein et al. 2019), coupled with the exploratory nature of this work, we also flag outcome measures with *p* < 0.10 and associated effect sizes (identifying potentially biologically relevant changes for further consideration).

## Results

### Metabolic influences of WD feeding and CS

**Fig. 1:**
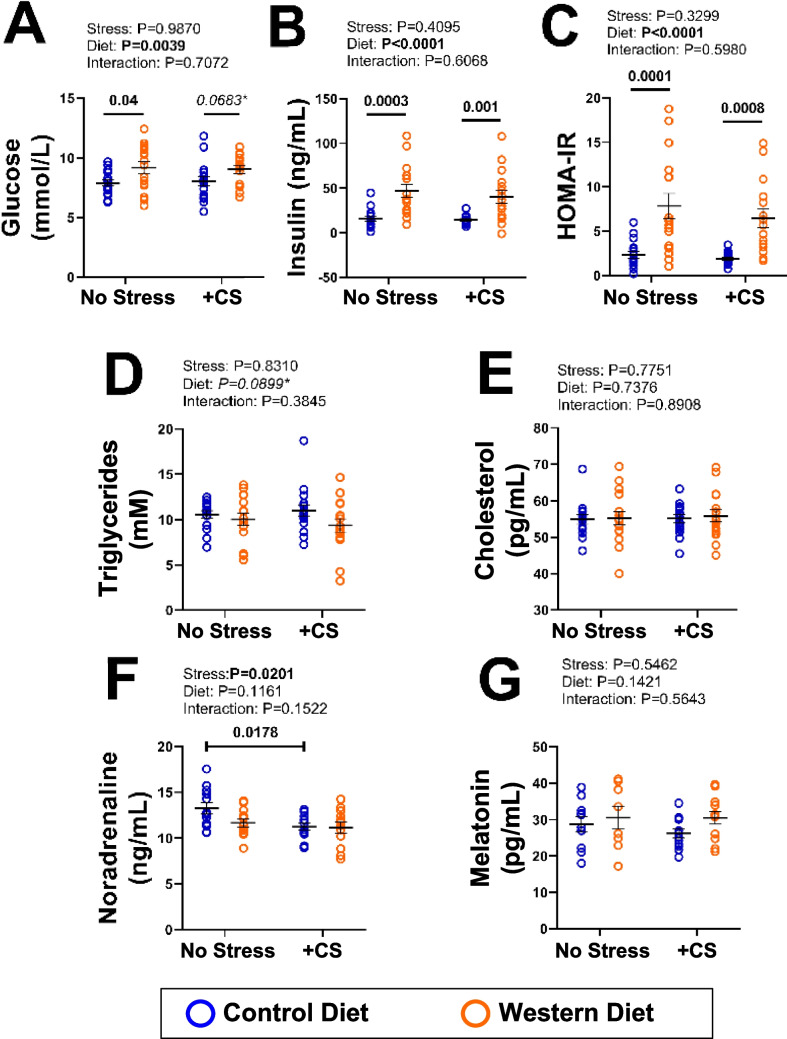
Circulating metabolic and neuroendocrine factors in male mice fed a control (CD) or Western diet (WD) for 20 wks ± chronic stress (CS) in the final 2 wks. Results shown for: **A**) glucose; **B**) fasting insulin; **C**) HOMA-IR; **D**) triglycerides; **E**) cholesterol; **F**) noradrenaline; and **G**) melatonin. Data analyzed via 2-way ANOVA with a Holm-Sidak post-hoc test where appropriate (*p*-values provided in capped or uncapped lines). Statistically significant results (at *p* < 0.05) are highlighted in bold. Data shown as means ± SEM (*n* = 16/group)

The WD increased body weight by ~ 15% (compared with CD mice) over 20 wks (Table 1). Mild stress reduced final weight by ~ 10% in both CD and WD mice, reversing weight gain to loss over the final 2 wks (Table 1). We estimated food intakes from mean values for 4 cages per group: while not significantly differing (reflecting, in part, resultant power limitation), this indicates ~ 20% higher daily caloric intake with the WD, and a tendency to reduced food intake with stress in WD mice (Table 1). Metabolic and endocrine outcomes are summarized in Fig. 1. Significant main effects of diet were identified for fasting glucose [*F*(1,60) = 9.019, *p* = 0.0039, η^2^_p_ = 0.13], insulin [*F*(1,59) = 28.36, *p* < 0.0001, η^2^_p_ = 0.33] and HOMA-IR [*F*(1,59) = 31.11, *p* < 0.0001, η^2^_p_ = 0.35] (Fig. 1). Post-hoc analysis identified WD-induced elevations in fasting glucose (no stress (NS), *p* = 0.04, CI_95_ = 8.2–10.2), fasting insulin (NS, *p* = 0.0003, CI_95_ = 32.1–62.3; CS, *p* = 0.001, CI_95_ = 24.8–55.3) and HOMA-IR (NS, *p* = 0.0001, CI_95_ = 4.9–10.9; CS, *p* = 0.0006, CI_95_ 24.8–55.3). A WD related increase in glucose in CS mice was also evidenced at *p* = 0.068 (CI_95_ 8.3–9.8), with a moderate effect size (η^2^_p_ = 0.13) (Fig. 1A). Interestingly, metabolic factors were largely unchanged in mice subjected to stress alone (Fig. 1), although CS did reduce weight by ~ 10% (Table 1). Circulating triglycerides, cholesterol and melatonin were unaltered (Fig. 1), while noradrenaline declined with CS in CD mice (*p* = 0.0201, η^2^_p_ = 0.12, CI_95_ = 10–6–11.9.9).

**Table 1:** Measures of body weight, weight change and caloric intake

	Control Diet (CD)	Western Diet (WD	Control Diet + Stress (CD + CS)	Western Diet + Stress (WD + CS)
Initial Body Weight (g)	23.1 ± 0.1	22.2 ± 0.1	22.4 ± 0.1	22.7 ± 0.1
Final Body Weight (g)	34.8 ± 0.7	39.5 ± 1.2 *p* < *0.001 vs. CD* *CI*_*95%*_* 36.9–42.1, η*^*2*^_*p*_ = *0.36*	31.2 ± 0.3 *p* < *0.0001 vs. CD* *CI*_*95%*_* 30.6–31.9, η*^*2*^_*p*_ = *0.21*	35.0 ± 0.7 *p* < *0.0001 vs CD* + *CS* *CI*_*95%*_* 34.5–38.0, η*^*2*^_*p*_ = *0.36*
∆ Weight Final 2 Wks (g)	1.14 ± 0.17	2.17 ± 0.20	−1.44 ± 0.85 *p* < *0.0001 vs. CD, WD* *CI*_*95%*_* −1.73—−1.14, η*^*2*^_*p*_ = *0.88, 0.04*	−2.71 ± 0.20 *p* < *0.0001 vs WD, CD* *CI*_*95%*_* −2.33—−3.10, η*^*2*^_*p*_ = *0.88, 0.04*
Final Caloric Intake (kJ/mouse/day)	27.0 ± 1.0	32.3 ± 1.1	27.2 ± 0.4	28.6 ± 0.6

Body weight (from wks 1–20) and caloric intake (from cage consumption, wks 5–20) in male mice fed a control (CD) or Western diet (WD) for 20 wks ± chronic stress (CS) in the final 2 wks. Data analyzed via 2-way ANOVA and Holm-Sidak post-hoc comparisons. Data shown as mean ± SEM (*n* = 16/group; excluding food intake which was estimated from mean values for 4 cages of 4 mice, per group). *P*-values indicating significant differences are detailed within the Table

### Behavioral changes with WD feeding and CS

**Fig. 2:**
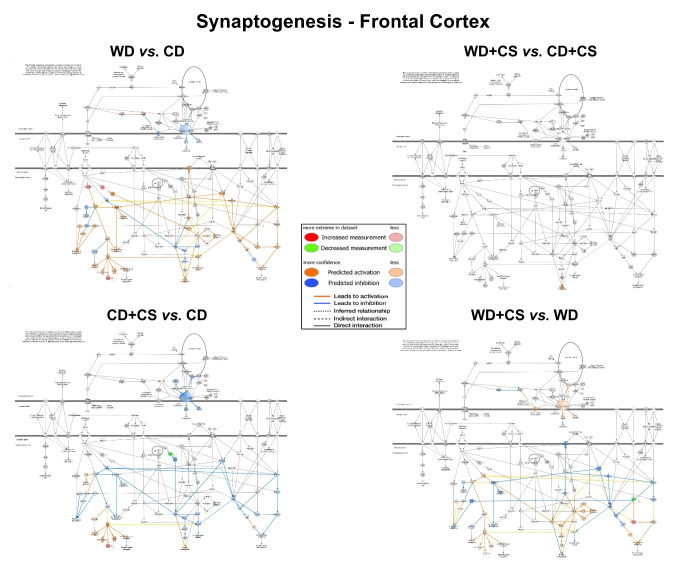
Open field and sucrose preference test outcomes in male mice fed a control (CD) or Western diet (WD) for 20 wks ± chronic stress (CS) in the final 2 wks. Results shown for: **A**) distance travelled (cm); **B**) periphery zone entries/center square entries (ratio); **C)** wall-seeking frequency (number); **D**) inactivity duration (seconds); **E**) rearing frequency (number); **F**) rearing duration (seconds); and** G**) sucrose preference. Data analyzed via 2-way ANOVA with a Holm-Sidak post-hoc test where appropriate (*p*-values provided in capped or uncapped lines). Statistically significant results (at *p* < 0.05) are highlighted in bold. Data shown as means ± SEM (*n* = 16/group)

Two-way ANOVA identified significant effects of stress on locomotor activity [*F*(1,60) = 40.68, *p* < 0.0001, η^2^_p_ = 0.40, CI_95_ = 2650–3269 and 2568–3111 for CS in CD and WD] and wall-seeking behavior [*F*(1,60) = 10.97, *p* = 0.0016, η^2^_p_ = 0.16, CI_95_ = 38.9–51.5 and 39.7–45.7 for CS in CD and WD], with post-hoc analysis supporting a CS-dependent increases in locomotion and wall-seeking in both diet groups (Fig. 2). Conversely, sucrose preference was significantly influenced by diet [*F*(1,58) = 45.78, *p* < 0.0001, η^2^_p_ = 0.44], with a significant decline with WD feeding in both un-stressed (*p* < 0.0001, CI_95_ = 0.47–0.64) and stressed (*p* < 0.0001, CI_95_ = 0.47–0.68) mice (Fig. 2). This supports a diet-dependent, stress-independent influence on sucrose preference.

### Changes in neurotransmitter and hormone levels with WD feeding and CS

#### Frontal cortex

**Fig. 3:**
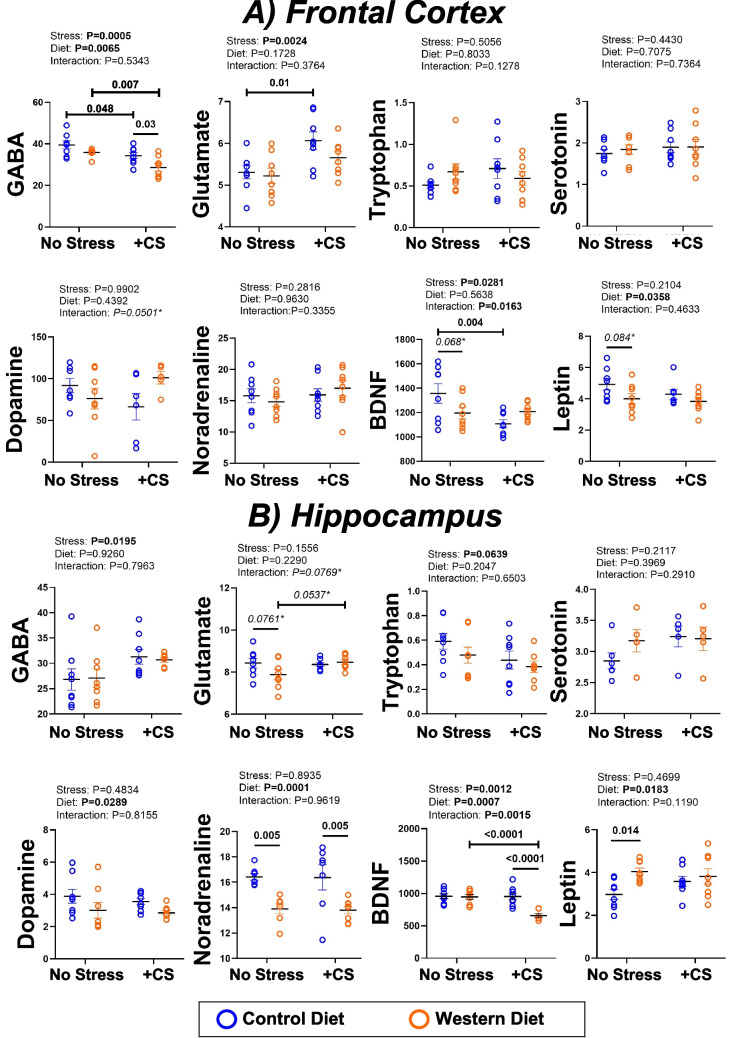
Neurochemical outcomes in male mice fed a control diet (CD) or Western diet (WD) for 20 wks ± chronic stress (CS) in the final 2 wks. Results are shown for** A**) frontal cortex and **B**) hippocampus. Mediators assessed include: GABA (pg/ml), glutamate (µg/ml), tryptophan (µg/ml), serotonin (ng/ml), dopamine (ng/ml), noradrenaline (ng/ml), BDNF (pg/ml) and leptin (ng/ml). Data analyzed via 2-way ANOVA, with a Holm-Sidak test for post-hoc comparisons (*p-*values provided in capped or uncapped lines). Statistically significant results (at *p* < 0.05) are highlighted in bold. Potentially relevant results (*p* < 0.10) are *italicized* and highlighted with an asterisk. Data shown as means ± SEM (*n* = 8/group)

Stress significantly reduced GABA (η^2^_p_ = 0.37, CI_95_ = 30.8–37.7 and 24.4–32.8 in CD and WD) and BDNF (η^2^_p_ = 0.16, CI_95_ = 1028–1187 and 1151–1265 for CS in CD and WD) and increased glutamate (η^2^_p_ = 0.29, CI_95_ = 5.6–6.6 in CD mice) in FC tissue (Fig. 3A). The WD reduced GABA in stressed mice (η^2^_p_ = 0.24, CI_95_ = 24.4–32.8). In addition, there was some evidence of WD related falls in BDNF and leptin in un-stressed mice, at *p-*values of 0.068 and 0.084; although the BDNF effect size was small (η^2^_p_ = 0.01, CI_95_ = 1082–1310) whereas the leptin effect was large (η^2^_p_ = 0.15, CI_95_ = 3.2–4.8) (Fig. 3A).

#### Hippocampus

There were fewer neurochemical changes in HPC (Fig. 3B). However, stress significantly reduced BDNF in WD (not CD) mice (η^2^_p_ = 0.33, CI_95_ = 595–724), with values also lower in stressed WD *vs*. stressed CD mice (η^2^_p_ = 0.32, CI_95_ = 595–724). There was also evidence of a WD dependent increase in glutamate, at *p* = 0.076 in un-stressed mice (CI_95_ = 7.3–8.4) and *p* = 0.054 in CS mice (CI_95_ = 8.2–8.7), though the effect size was only moderate (η^2^_p_ = 0.05). Hippocampal leptin was increased by the WD in un-stressed mice (η^2^_p_ = 0.18, CI_95_ = 3.7–4.4), while noradrenaline was reduced by the WD in both un-stressed and stressed mice (η^2^_p_ = 0.46, CI_95_ = 13.0–14.8 and 13.1–14.5 for NS and CS) (Fig. 3B).

### Mitochondrial function changes with WD feeding and CS

#### FC and HPC

**Fig. 4:**
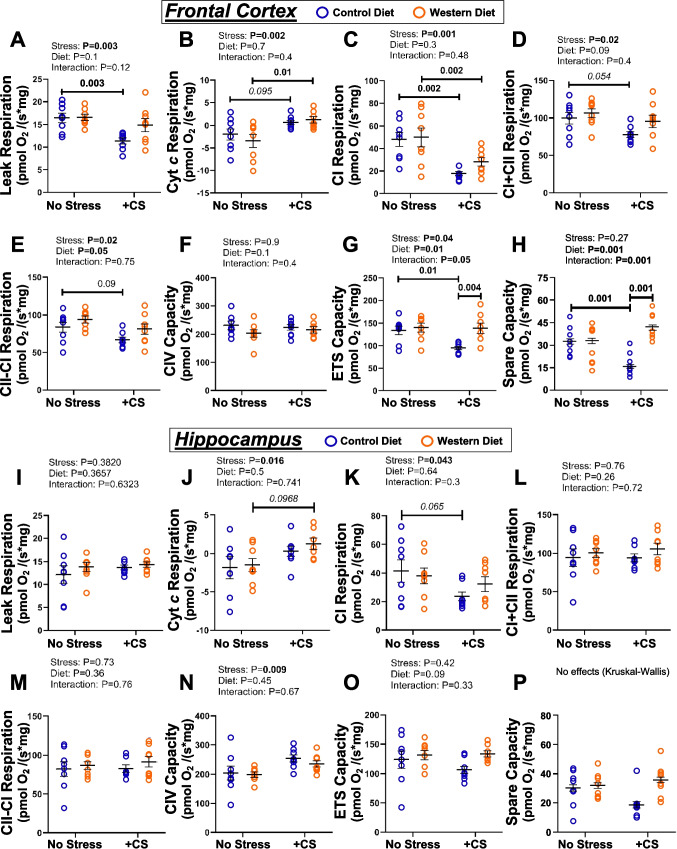
Mitochondrial respiratory function in male mice fed a control diet (CD) or Western diet (WD) for 20 wks ± chronic stress (CS) in the final 2 wks. Results shown for frontal cortex (**A-H**) and hippocampus (**I-P**). Measures include: **A**) & **I**) leak respiration; **B**) & **J**) outer mitochondrial membrane integrity (indicated by cytochrome *c* respiration); **C)** & **K)** Complex I respiration; **D**) & **L**) Complex I + II respiration; **E**) &** M**) Complex II respiration; **F**) &** N**) Complex IV capacity; **G**) &** O**) maximum respiratory capacity; and** H**) &** P**) spare respiratory capacity. Data analyzed via 2-way ANOVA, with a Holm-Sidak test for post-hoc comparisons where appropriate (*p-*values provided in capped or uncapped lines). Statistically significant results (at *p* < 0.05) are highlighted in bold. Potentially relevant results (*p* < 0.10) are *italicized* and highlighted with an asterisk. Data shown as means ± SEM (*n* = 8/group)

Mitochondrial respiration in FC tissue was impaired with CS, while WD feeding had limited independent impact (Fig. 4). Combined CS in WD fed mice produced a mix of outcomes without further worsening respiratory function. Chronic stress reduced leak respiration in CD mice (Fig. 4A; η^2^_p_ = 0.28, CI_95_ = 9.8–13.0), CI respiration in both diet groups (Fig. 4C; η^2^_p_ = 0.42, CI_95_ = 13.9–21.9 and 18.8–37.4 in CD and WD mice), and maximum ETS (Fig. 4G; η^2^_p_ = 0.15, CI_95_ = 84.5–103.4) and spare respiratory capacity (Fig. 4H; η^2^_p_ = 0.15, CI_95_ = 9.8–21.4) in CD mice. There were minimal influences of stress on CIV capacity (Fig. 4F), while CS increased cytochrome *c* dependent respiration in WD (Fig. 4B; η^2^_p_ = 0.29, CI_95_ = −0.4–2.8), together with evidence of a strong effect (η^2^_p_ = 0.29) in CD mice, though at *p* = 0.095 (Fig. 4B). The ANOVA supported inhibitory effects of CS on CI + CII (Fig. 4D) and CII-CI respiration (Fig. 4E), though no differences were evident at *p* < 0.05 in post-hoc analyses. However, there was evidence of CS-related reductions in CI + CII and CII-CI in CD mice, with large effect sizes (η^2^_p_ = 0.17 and 0.20) at *p* = 0.054 and 0.09, respectively (Fig. 4D and E).

The WD alone had no significant effect on routine measures of respiration (or FCR values) in FC. However, as noted above, co-morbid stress in WD mice increased cytochrome *c* dependent respiration (Fig. 4B), and reduced CI respiration (Fig. 4C). Interestingly, CS-dependent reductions in maximum ETS (Fig. 4G) and spare respiratory capacities (Fig. 4H) were only evident in CD mice, with the WD apparently limiting these stress effects. As a result, both measures were higher in stressed WD *vs*. stressed CD mice (η^2^_p_ = 0.21, CI_95_ = 110.6–164.6 and 34.2–48.9 for ETS and spare respiratory capacities).

Mitochondrial respiration in HPC appeared less sensitive to CS and WD than FC (Fig. 4I-P). Measures of respiration appeared generally insensitive to CS alone, although there was evidence of a fall in CI flux in CD mice at *p* = 0.065, with a substantial effect size (η^2^_p_ = 0.14, CI_95_ = 16.4–30.9) (Fig. 4K). No WD-dependent changes in routine respiration were detected in HPC (Fig. 4I-P). Co-morbid CS in WD mice also failed to substantially influence HPC respiration, apart from evidence of a CS elevation in cytochrome *c* dependent respiration in WD mice at *p* = 0.097, with a large effect size (η^2^_p_ = 0.20, CI_95_ = −0.12–2.98) (Fig. 4J).

Flux control ratios in FC and HPC generally mirrored patterns of change in respiratory function (Table 2). Stress decreased the FCR for CI and increased CII and CI + CII FCRs (Table 2). The FCR values in HPC also generally mirrored flux responses (Table 2). No WD-dependent changes in FCR values were detected in HPC. However, FCRs for CII and CI + CII were selectively decreased by CS in WD and not CD mice (Table 2).

**Table 2:** Mitochondrial flux control ratios in FC and HPC

	Control Diet (CD)	Western Diet (WD	Control Diet + Stress (CD + CS)	Western Diet + Stress (WD + CS)
FRONTAL CORTEX				
CI FCR	0.36 ± 0.03	0.35 ± 0.05	0.19 ± 0.01 *p* < *0.005 vs. CD* *CI*_*95*_* 0.16–0.22, η*^*2*^_*p*_ = *0.46*	0.20 ± 0.02 *p* < *0.005 vs. WD* *CI*_*95*_* 0.16–0.24, η*^*2*^_*p*_ = *0.46*
CII FCR	0.40 ± 0.02	0.43 ± 0.05	0.66 ± 0.01 *p* < *0.0001 vs. CD* *CI*_*95*_* 0.63–0.69, η*^*2*^_*p*_ = *0.50*	0.50 ± 0.01 *p* < *0.01 vs. CD* + *CS* *CI*_*95*_* 0.46–0.53, η*^*2*^_*p*_ = *0.25*
LCR	0.12 ± 0.01	0.11 ± 0.01	0.12 ± 0.01	0.11 ± 0.01
CI + CII FCR	0.77 ± 0.01	0.77 ± 0.02	0.83 ± 0.02 *p* < *0.005 vs. CD* *CI*_*95*_* 0.78–0.89, η*^*2*^_*p*_ = *0.01*	0.70 ± 0.01 *p* = *0.051 vs. WD* *CI*_*95*_* 0.67–0.72, η*^*2*^_*p*_ = *0.01*
HIPPOCAMPUS				
CI FCR	0.33 ± 0.03	0.29 ± 0.03	0.19 ± 0.01 *p* < *0.01 vs. CD* *CI*_*95*_* 0.16–0.23, η*^*2*^_*p*_ = *0.29*	0.22 ± 0.02
CII FCR	0.44 ± 0.03	0.48 ± 0.03	0.67 ± 0.03 *p* < *0.0001 vs. CD* *CI*_*95*_* 0.59–0.75, η*^*2*^_*p*_ = *0.44*	0.53 ± 0.02 *p* < *0.01 vs. CD* + *CS* *CI*_*95*_* 0.49–0.56, η*^*2*^_*p*_ = *0.10*
LCR	0.10 ± 0.01	0.10 ± 0.01	0.12 ± 0.01	0.10 ± 0.01
CI + CII FCR	0.77 ± 0.02	0.76 ± 0.01	0.84 ± 0.03 *p* < *0.05 vs. CD* *CI*_*95*_* 0.77–0.91, η*^*2*^_*p*_ = *0.06*	0.75 ± 0.02 *p* < *0.01 vs. CD* + *CS* *CI*_*95*_* 0.69–0.80, η*^*2*^_*p*_ = *0.15*

Mitochondrial flux control ratios determined in male mice fed a control diet (CD) or Western diet (WD) for 20 wks ± chronic stress (CS) in the final 2 wks. Measures include FCRs for Complex I, Complex II and leak respiration (LCR), and Complex I + II-linked respiration. Data analyzed via 2-way ANOVA, with a Holm-Sidak test for post-hoc comparisons. Data shown as means ± SEM (*n* = 8/group). *P*-values indicating significant differences are detailed within the Table, with effect sizes (η^2^_p_) and 95% confidence intervals (CI_95_) for altered variables

#### Hypothalamus and nucleus accumbens

We also assessed hypothalamus and nucleus accumbens respiratory function, with outcomes presented in the Data Supplement (Figs. S1-S4). Both regions were largely insensitive to a WD and CS. Interestingly, CIV fluxes for hypothalamus were selectively increased by CS or WD feeding, but not their combination (Fig. S1). No FCRs were altered in hypothalamus (Fig. S2). In nucleus accumbens, CS in WD mice increased CI + CII respiration above that with CS in CD mice (Fig. S3), and select FCR changes emerged (Fig. S4): reduced CI FCR in WD + CS mice, and CI + CII FCR with CS alone (similar trend in WD + CS tissue). Combined WD + CS increased spare respiratory capacity relative to the WD, and CII-CI respiration relative to WD or CS.

### Proteome changes with WD feeding and CS

Proteome analysis identified ~ 2100 proteins across FC (CD, 2124; CD + CS, 2149; WD, 2170; WD + CS, 2108) and HPC (CD, 2114; CD + CS, 2090; WD, 2116; WD + CS, 2063). Differentially expressed proteins (DEPs) were identified via a combination of statistical and empirical thresholds to ensure high confidence in protein abundance differences. For DEPs in FC (Figs. S5-S8): main CS effect = 69; main WD effect = 58; combination effect = 50 proteins. For DEPs identified in HPC (Figs. S9-S12): main CS effect = 104; main WD effect = 76; combination effect = 75 proteins.

To explore biological processes and mechanisms, we examined cellular component/function and undertook pathway enrichment/functional network analyses. At a cell component level in FC, CS and WD predominantly influenced pre- and post-synaptic proteins (particularly glutamatergic) together with cytosolic and mitochondrial components (Figs. S5-S7). The WD additionally altered elements of the myelin sheath/neuron projection.

Within HPC, CS influenced proteins of the myelin sheath, synapses (pre- and post-synapse, glutamatergic) and axon; followed by cytosolic, neuron projection and mitochondrial elements (Figs. S9-S11). The WD influenced proteins within the cytosol, exosome, neuronal body and neurofilaments (Fig. S10.

In terms of FC biological functions (Figs. S5-S7, panel B): CS alone influenced protein translation, proteasomal protein catabolism, mitochondrial respiration/TCA cycle and protein tyrosine kinase processes; WD feeding modified nerve growth/development and plasticity, and also influenced proteasomal protein catabolism, protein tyrosine kinase processes and the oxidative stress response. Co-morbid stress in WD mice primarily modified nerve growth, development and death processes, suggesting more direct disruption of FC neurogenesis and neuronal death under co-morbid conditions. Regarding HPC biological functions (Figs. S9-S11, panel B): CS and a WD each modified nerve growth/development processes, with stress additionally modifying endocytosis/exocytosis. Co-morbid CS in WD mice significantly modified synaptic exocytosis/endocytosis processes and purine nucleotide biosynthesis.

Pathway changes from z-score analysis provide information on general up- or down-regulation of overall networks. In FC, stress selectively up-regulated EIF2 and CMA pathway components (Figs. S5C and S8). While CS up-regulated CMA in CD mice, the opposite was evident in WD mice, with general down-regulation of the path in co-morbid mice (Fig. S8). Interestingly (and contrasting greater changes in mitochondrial function, neurotransmitter and BDNF levels within FC), a substantially higher number of stress-sensitive paths were evident in HPC *vs.* FC (Fig. S9C). Almost all were down-regulated (including PKA, melatonin, p70s6k, neuropathic pain, 14–3-3, apelin, insulin secretion, synaptic long-term potentiation/depression, synaptogenesis and mitochondrial dysfunction pathways), with only CMA and GPCR integration of enteroendocrine signaling paths up-regulated. This suggests a broader stress reactivity in HPC paths *vs.* more select sensitivities in FC.

The WD alone exacerbated FC mitochondrial dysfunction while up-regulating reelin and synaptogenesis pathways (Fig. S6C). The WD similarly up-regulated CMA and synaptogenesis paths in HPC, together with actin cytoskeleton and integrin signaling. However, the WD also down-regulated hippocampal PKA, NO and ROS, and epithelial adherens junction pathway elements. In FC, co-morbid CS + WD down-regulated mitochondrial dysfunction and CMA pathways, and up-regulated neuronal reelin signaling (Figs. S7C and S8). In HPC, co-morbid CS and a WD up-regulated SNARE, integrin and synaptogenesis signaling (Figs. S11C and S12). As for FC, CS appears to up-regulate the CMA path in HPC of CD mice, and down-regulate the path in WD mice (Fig. S12). Deeper investigation of select responses permits assessment of the potential relevance of these changes (see *3.6* below).

### Select pathway responses: mitochondrial dysfunction, CMA, synaptogenesis, reelin, NRF2 and EIF2

Top pathways modified across regions were associated with energy regulation (mitochondrial function), translational control (EIF2 signaling), nerve growth/remodeling (synaptogenesis, reelin signaling) and cell signaling (integrin signaling) (Figs. S8 and S12). To explore functional outcomes, we focus on paths governing mitochondrial (dys)function, cellular quality control (CMA), synaptogenesis and associated reelin signaling, and defenses against cell stress (NRF2, EIF2). Details of the reelin, NRF2 and EIF2 pathway responses are included in the Data Supplement (Figs. S13-S15). Table 3 summarizes FC neurobiological and pathway responses to CS that appear to be shared/additive with those for the WD, inhibited/reversed by a co-morbid WD, or only evident under co-morbid conditions.

**Table 3:**
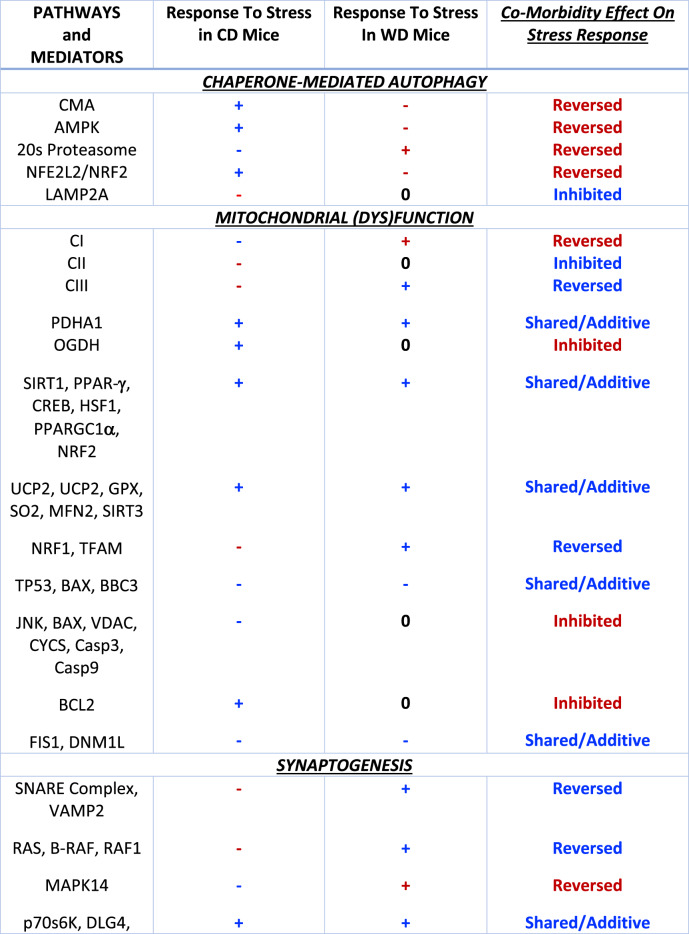

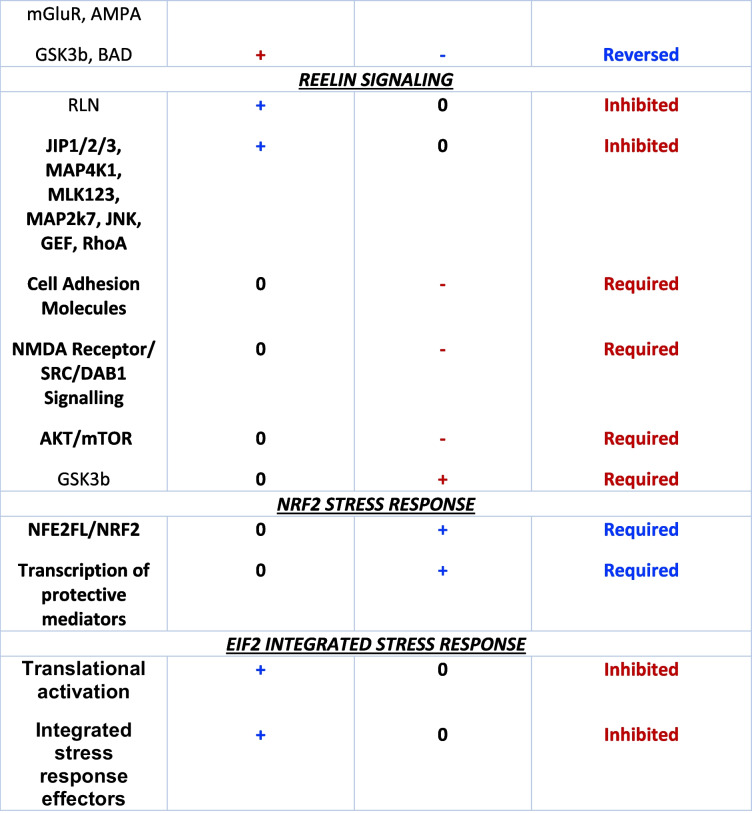
Summary of select FC proteome pathway responses to CS that are shared or additive, inhibited/reversed, or that are only evident under co-morbid (CS + WD) conditions

**Fig. 5:**
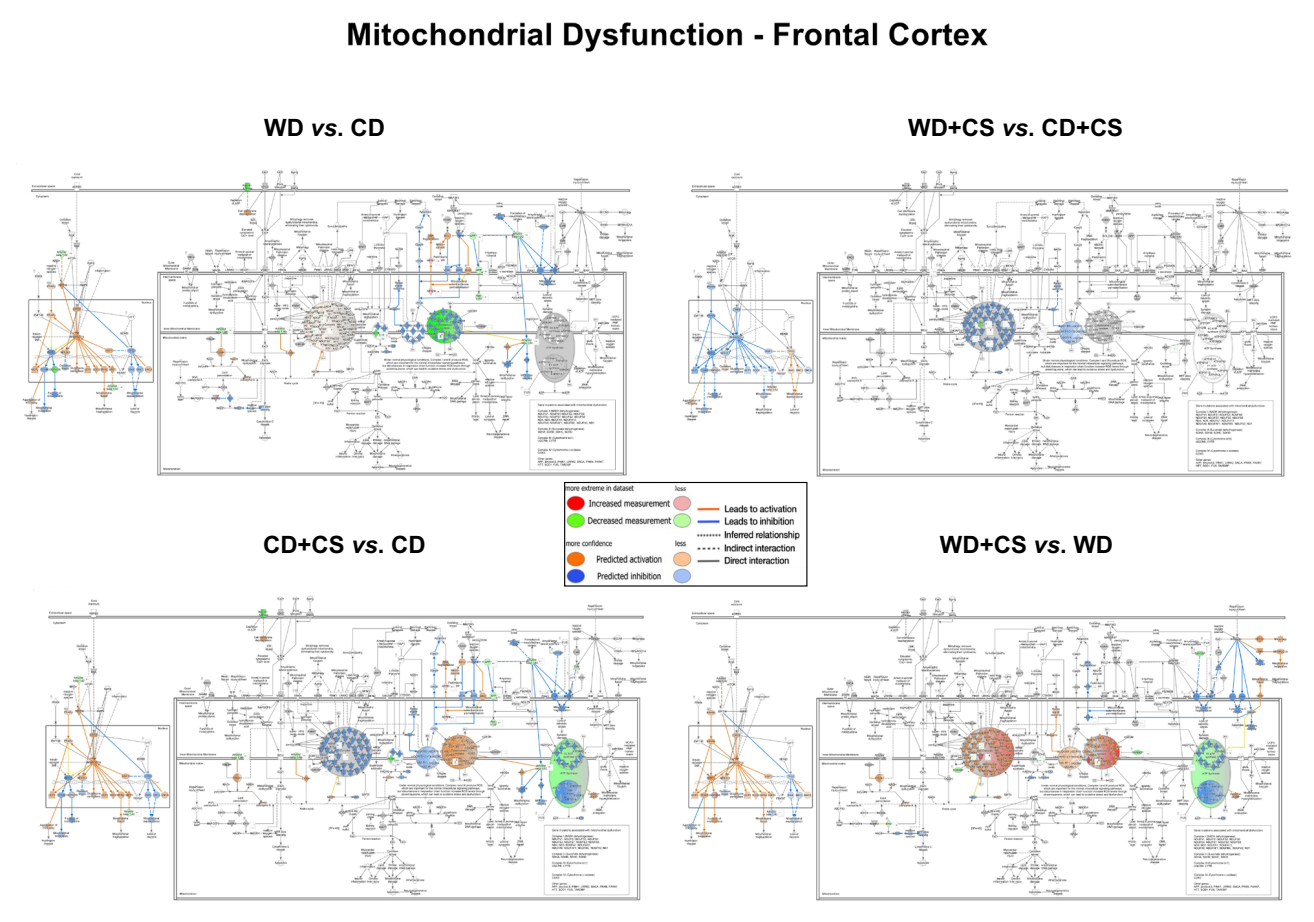
Effects of CS and a WD on the “mitochondrial dysfunction” pathway in FC tissue. Shown are the influences of the WD alone (WD vs. CD), stress alone (CD + CS vs. CD), stress in WD mice (WD + CS vs. WD), and of diet in stressed mice (WD + CS vs. CD + CS)

**Fig. 6:**
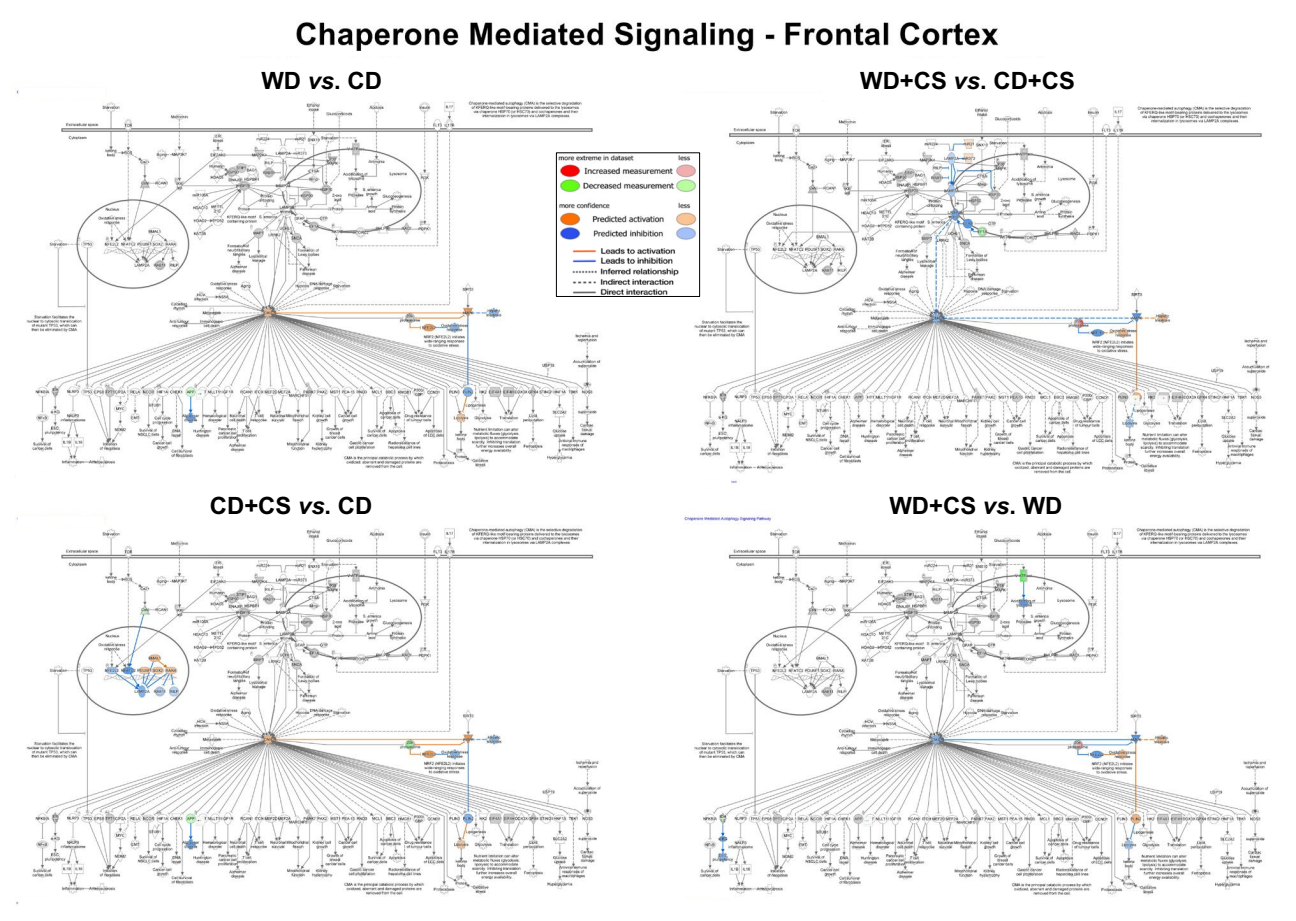
Effects of CS and a WD on the “chaperone mediated autophagy signaling” pathway in FC tissue. Shown are influences of the WD alone (WD vs. CD), stress alone (CD + CS vs. CD), stress in WD mice (WD + CS vs. WD), and of diet in stressed mice (WD + CS vs. CD + CS)

**Fig. 7:**
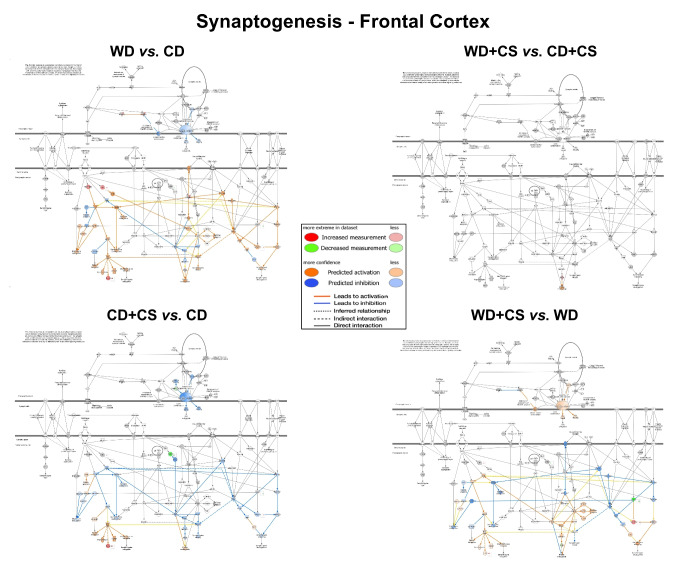
Effects of CS and a WD on the “synaptogenesis signaling” pathway in FC tissue. Shown are influences of the WD alone (WD vs. CD), stress alone (CD + CS vs. CD), stress in WD mice (WD + CS vs. WD), and of diet in stressed mice (WD + CS vs. CD + CS)

Pathway responses to CS in CD and WD mice, and effects of co-morbidity. Pathway elements are either activated (+), inhibited (-) or unaltered (0). Blue indicates a response (or effect of co-morbidity) predicted to be adaptive/neuroprotective; Red indicates a response (or effect of co-morbidity) predicted to be maladaptive/detrimental. See Figs. 5, 6, and 7 and S13-S15 for pathway changes

#### Mitochondrial (dys)function in FC

Consistent with FC respiratory changes, the mitochondrial (dys)function pathway was one of the most significantly modified (Fig. 5). Effects of diet (NADH related CI activation; suppressed CII, CIII and CIV expression/activity) and stress (suppressed CI, CIII and ATP-synthase expression/activity; CIV activation) were distinct, though influences on related nuclear signaling were almost identical. These mixed effects on respiratory complexes are somewhat consistent with respiratory function changes (Fig. 4, Table 2). Both CS and a WD appear to induce adaptive changes in nuclear signaling. Stress-dependent promotion of SIRT1, PPAR-γ and CREB activation of nuclear PPARGC1α is predicted to enhance NRF2 protection, anti-oxidant capacity, mitochondrial fusion and SIRT3 activity; and inhibit TP53 death signaling and mitochondrial depolarization (Fig. 5). Reduced cytosolic amyloid β1–42 expression will also lower oxidative stress, MPTP activity and mitochondrial fragmentation; and activate pyruvate/TCA cycle metabolism. Inhibition of cytosolic JNK, activation of cytosolic BCL2, and downstream inhibition of BAX, VDAC and CYCS may limit membrane permeabilization and apoptosis; while inhibition of FIS1 and DNM1L will limit mitochondrial fragmentation. In contrast, beneficial NRF1/TFAM mediated mitochondrial biogenesis may be inhibited by CS.

The WD induced similar beneficial changes in nuclear signaling, and in JNK, BCL2, BAX, VDAC and CYCS activities (Fig. 5). Lower CaMK2 levels may additionally limit mPTP activity. As for CS, the WD may also inhibit NRF1/TFAM mitochondrial biogenesis. Effects on CII and CIII are similar to those of stress, while proteome changes support reduced rather than increased CIV expression and activity, and a potential activation of CI (with no clear shifts in ATP-synthase). This mix of changes may explain general lack of effect of the WD on respiratory fluxes.

Subjecting WD mice to co-morbid stress resulted in a distinct increase in activities or expression of CI, CIII and CIV, and reduced activity/expression of ATP-synthase elements—reflecting a reversal of the effects of stress alone on CI and CII, and a common inhibitory influence on ATP synthase in both diet groups. Direct comparison supports greater reductions in CI and CIII activities with co-morbid WD + CS *vs.* CS alone, and reversal of the influence of CS on nuclear signaling—reduced beneficial (PPAR-γ, PPARGC1α, CREB, NRF1, NRF2) and enhanced detrimental (TP53, BAX, BBC3) signaling (Fig. 5).

#### Chaperone-mediated autophagy in FC

Stress appears to activate CMA, involving AMPK stimulation and reduced expression of inhibitory 20 s proteasome (Fig. 6). However, lysosomal LAMP2A receptor expression may fall with reduced NRF2/NFAT2c activities (and upstream calcineurin expression), and increased activities of inhibitors (BMAL1, SOX2, RARA, POU5F). A stress-dependent decline in APP is likely protective. The WD alone is also predicted to activate CMA via AMPK, though without modifying nuclear determinants of LAMP2A expression (Fig. 6). In contrast, co-morbid CS + WD may repress CMA via AMPK inhibition (and increased 20 s proteasome expression), and limit lysosomal acidification via reduced V-ATPase expression. Direct comparison of WD + CS *vs*. CD + CS mice supports a relative decline in AMPK activity and elevation in 20 s proteasome in the co-morbid group, with reduced lysosomal LAMP2A function (linked to inhibitory miR21 and miR373, and declining Ef1a). A relative decline in Nrf2 activity favors oxidative stress.

These profiles collectively support AMPK/20 s proteasome related activation of CMA with stress or a WD, with distinct effects on nuclear determinants: stress-specific NRF2/NFAT2c inhibition and BMAL1, SOX2, RARA and POU5F1 activation *vs.* no independent influence of WD feeding. In contrast to these adaptations, stress in WD fed mice may inhibit CMA via opposing changes in AMPK/20 s proteasome and relative reductions in lysosomal LAMP2A function and NRF2 protection.

#### Synaptogenesis in FC

Stress and WD feeding had opposing influences on synaptogenesis (Fig. 7). Most arms of synaptogenesis signaling appear inhibited by stress, including complexin expression and SNARE complex assembly, although p70S6k dependent protein translation, mGluR and AMPA receptor activities may be selectively activated, together with DLG4 expression. These changes suggest a net inhibitory influence of stress, despite potentially adaptive changes at the level of p70S6K related elements. A WD may also limit SNARE complex formation and other elements of synaptogenesis (including RAS, RAF1, BRAF signaling); however contrasting the influence of CS, the WD is predicted to broadly stimulate most elements of the synaptogenesis pathway, including increased CRK/CRKL and DLG4 expression (Fig. 7).

Imposition of stress in WD mice shares some similarities with stress in CD mice, including reduced activities of CREB, TIAM, RAF1/ERK1/2 and RAC1-PAK1-LIMK1 signaling; and increased p70S6K related signaling (Fig. 7). However, SNARE complex formation may be activated. (as are RAS, RAF1, BRAF and RAP signaling), while CDK5 and WASF1 levels are selectively reduced and increased, respectively. These profiles support a negative impact of stress on synaptogenesis in CD and WD mice, whereas a WD alone has some stimulatory influences.

#### Reelin signaling in FC

Stress is predicted to reduce APP expression (and amyloid ß plaque accumulation), and selectively activate IP1/2/3-Gef-RHOA signaling (and downstream cytoskeleton rearrangement) together with parallel MAP4K1-JNK activity (promoting SAPK/JNK signaling, brain development) (Fig. S13). The WD had more extensive effects, additionally increasing CRK and CaMK2 expression and activities of associated Akt-mTOR (mediating dendrite growth/neurogenesis), ERK1/2 (mediating long-term potentiation of hippocampal neurons) and the RAPGEF1-Rap1 associated integrin α5ß1/NECTIN3/CDH2 pathways (promoting cell migration/adhesion, synaptogenesis, synaptic maintenance). In contrast, CS in WD mice inhibited most elements of the reelin path, and decreased CDK5 while increasing WASF1 expression (Fig. S13). There is thus evidence stress and a WD independently promote reelin signaling and associated neurogenesis, synaptogenesis and neuroplasticity, whereas co-morbidity inhibits these outcomes.

#### NRF2 mediated oxidative stress response in FC

The CS protocol alone did not modify the NRF2 pathway in FC, while the WD inhibits cytosolic and nuclear elements of this path (Fig. S14). Although CS appears to activate the NRF2 path in WD mice (suggesting greater oxidant stress), direct comparison indicates a relative decline in cytosolic and nuclear NRF2 signaling in WD + CS *vs.* CD + CS mice, congruent with greater dysfunction under co-morbid conditions.

#### EIF2 pathway response in FC

Stress alone almost exclusively activated elements of EIF2 signaling in FC tissue (Fig. S15). The WD had negligible effects alone, and appeared to eliminate stress sensitivity of EIF2 signaling. Direct comparison confirms a broad activation of EIF2 by stress in CD *vs*. WD mice (Fig. S15).

#### Hippocampal pathway responses

Mitochondrial pathway changes with CS suggest beneficial activation of nuclear PPAR-γ, PPARGC1α, CREB, NRF1 and NRF2 signaling, and detrimental activation of TP53/BCL-dependent mitochondrial depolarization and cell loss in HPC (Fig. S16). Other beneficial responses include reduced FIS1 expression, and cellular and mitochondrial Ca^2+^ entry. This array of beneficial changes, coupled with select effects that include CI activation and CIII inhibition, is consistent with generally unaltered respiratory fluxes in HPC. Similarly, the WD had no consistent influence on mitochondrial processes, whereas co-morbid stress in WD mice may: beneficially activate nuclear signaling (including STAT1, PPAR-γ, PPARGC1α, CREB, NRF1/NRF2 signaling); down-regulate detrimental TP53/BCL protein signaling; increase CI and CII activities, and the activity/expression of ATP-synthase elements; and inhibit CIV activity/expression. This complex response may explain limited respiratory function changes in HPC, while there is clear evidence of adaptations via STAT1, PPAR-γ, PPARGC1α, CREB, NRF1 and NRF2 signaling.

As for FC, hippocampal CMA appears stimulated by stress or a WD, involving AMPK activation and reduced 20 s proteasome expression (Fig. S17). However, there is evidence for reduced activities of the HSP90-proteasome, lysosomal LAMP2A and lysosomal acidification. The effects of CS on HPC CMA were similar in both CD and WD mice, with the latter also involving AMPK activation and reduced lysosomal Lamp2a activity.

The synaptogenesis pathway response in HPC was also mixed (Fig. S18). Both CS and a WD reduced SNARE complex formation, and are predicted to activate: PI3K-AKT and BRAF/MAPK14/RAB5 signaling; microtubule formation via GSK3β sensitive MAP1B/MAPT/CLASP2 signaling; and synapsin function. However, while CS inhibited additional pathways (including p70S6k, CREB-TIAM, RAF1/ERK1/2, RAK1/PAK1/LIMK1 and WASF signaling), these were all activated by a WD. Co-morbid CS in WD mice had limited additional impact, potentially reducing SNARE complex function.

Chronic stress was inhibitory for reelin signaling in HPC, reducing CAMK2, APOE and ARP2-3 expression and inhibiting ERK1/2 activity (Fig. S19). The WD alone also reduced APOE together with RHOA expression. Stress in WD mice had limited influence on HPC reelin signaling. No differences in HPC reelin signaling emerged in WD + CS *vs.* CD + CS mice.

Contrasting the FC response, the NRF2 pathway in HPC was repressed by stress and activated by a WD (Fig. S20). This suggests a propensity for stress-driven oxidative damage in HPC (where NRF2 defenses may be inhibited) *vs*. FC. In HPC, CS up-regulated EIF2 signaling, with evidence of increased ribosomal 40 s subunit levels (Fig. S21). The WD had relatively minor effects, while CS in WD mice also up-regulated EIF2 signaling (albeit to a lesser extent).

## Discussion

Chronic stress and a WD are prevalent drivers of chronic affective and other disorders. However, their interactions are incompletely understood. Pre-clinical studies reveal both detrimental (Santos et al. 2018; Shively et al. 2023) and protective (Finger et al. 2011; Maniam et al. 2016) influences of high fat or WD feeding on stress reactivity and outcomes. Results of the present study reveal a particular stress sensitivity of FC *vs*. HPC, including significant mitochondrial dysfunction, reduced BDNF and altered glutamate:GABA balance. Interestingly, co-morbid CS in WD mice resulted in co-expression of anhedonia and anxiety-like behaviors; and the WD appears to have wider influences on neurochemical and respiratory measures under co-morbid conditions. Proteome analysis provides evidence co-morbid CS and a WD may reduce or even reverse stress-sensitive pathway responses (Table 3). This is consistent with the concept that chronic stressors may suppress or reverse adaptive feedback to increase central nervous system (CNS) vulnerability and disease development (Stapelberg et al. 2018, 2019).

### Behavioral and neurochemical effects of individual and co-morbid WD feeding and CS

Stress and Western-type diets induce anxiety or depressive behaviors in humans and animal models. Shifts in immuno-inflammatory, neurotransmitter and neurotrophic processes are implicated (Langgartner et al. 2019; Marx et al. 2021), with emerging support for mitochondrial (Ciubuc-Batcu et al. 2024) and gut microbiome involvement (Cruz-Pereira et al. 2020; Marx et al. 2021). The diet here models a human WD (estimated by the US Department of Agriculture to contain 35%, 49% and 16% calories as fat, carbohydrate and protein). Distinct from very high fat content diets (45–60% of calories) used to induce experimental obesity, this more biologically relevant formulation was predicted to moderately influence energy balance and weight (Hu et al. 2018). This is borne out by 15% weight gain, contrasting the profound obesity (*eg*. ≥ 60% weight gain) reported with very high fat diets. The moderate fat level may also explain limited anxiogenic influences of the WD alone: neurological perturbations with diets containing ≥ 60% fat substantially exceed those of lower fat level diets (*eg*. Morrison et al. 2010). Behavior may also be indirectly influenced by metabolic outcomes. For example, while wall-seeking and hyper-locomotion are consistent with anxiogenesis, locomotion may be influenced by body weight and system insulin sensitivity. Additionally, differing effects of lipids and other diet elements on brain lipid profiles, oxidative stress, inflammation and reward and satiety pathways (Klaus 2005; Sanchez et al. 2024; Sharma 2021) may contribute to distinct behavioral outcomes across diets.

Interestingly, WD feeding appears to have potentially detrimental influences on neurochemical measures in CS mice not evident in un-stressed mice (Fig. 3). The WD reduced FC GABA, and HPC BDNF and noradrenaline, under co-morbid conditions, contrasting no independent effect of the WD alone in FC (and altered HPC leptin and noradrenaline). Moreover, the WD had no independent effects on mitochondrial respiration, while modifying both FC and HPC respiratory parameters under co-morbid conditions. Our observations are collectively consistent with exaggerated neurobiological disruption under co-morbid conditions.

Reduced sucrose preference in WD mice suggests anhedonia (Fig. 2)—a robust outcome in rodents, compared with somewhat variable influences of stress. Previous investigations implicate leptin in WD-related anhedonia (Ates et al. 2014). Peripheral leptin inhibits taste responses to sweet compounds, and central leptin suppresses sucrose-dependent dopaminergic activation (Domingos et al. 2011). Interestingly, the WD increased HPC leptin, while tending to reduce FC leptin, effects evident only in un-stressed mice. Increased HPC leptin may reflect allostasis, limiting intake of hyper-caloric food (Kanoski et al. 2011) and contributing to anhedonia. This is consistent with stability of leptin levels and sucrose preference in CS mice. We report a link between declining FC and HPC leptin and anxiogenesis in a murine model of type 2 diabetes (Griffith et al. 2022), congruent with limited anxiogenic effects of the WD (which increased HPC leptin). Leptin signaling is distinct in FC *vs*. HPC (Burgos-Ramos et al. 2010), with these regions differentially regulated under different metabolic or feeding conditions. For example, MRI analysis reveals differential sub-cortical (HPC, hypothalamus) hyper-activation *vs*. cortical hypo-activation in Prader-Willi syndrome (genetic obesity involving extreme hyperphagia) compared with people with ‘simple’ obesity (or non-obese controls) (Holsen et al. 2012). The question arises as to whether the WD-dependent decline in sucrose preference reflects a broadly anhedonic outcome and/or diet specific shifts in leptin-dependent feeding behavior? It is notable in this regard that stress, alone or in combination with the WD, failed to modify sucrose preference.

We also observed region-specific changes in the inhibitory and excitatory neurotransmitters GABA and glutamate. This indicates a stress-dependent excitation:inhibition (E:I) imbalance in FC that may be exacerbated by a WD. This contrasts relative insensitivity of HPC neurotransmitter levels. Region-specific differences in sensitivity, and evidence of an E:I imbalance in FC, have been noted in models of chronic stress (Hasler et al. 2007) or high-fat feeding (Sandoval-Salazar et al. 2016). A more pronounced stress-dependent decline in GABA with WD feeding may reflect top-down control of GABAergic inhibitory processes to disrupt feeding behavior (Alonso-Alonso and Pascual-Leone 2007; Sandoval-Salazar et al. 2016). Few studies directly assess the interactive impacts of stress and a WD on glutamatergic pathways. However, it is clear diet has an influence, with caloric restriction, n-3 polyunsaturated fatty acid or glutamine supplementation all improving glutamatergic signaling and limiting affective or cognitive dysfunction (Fadó et al. 2022). A WD may also reduce AMPA and NMDA receptor subunits, and glutamate and glutamine levels (Fadó et al. 2022). Interestingly, Francis et al. (2022) report that a WD reduces the glutamatergic modulator kynurenic acid, the only biomarker significantly associated with depression symptoms. Co-morbid CS and a WD are thus predicted to worsen glutamatergic signaling via multiple molecular effects.

Roles of neurotrophic factors are of interest, with BDNF essential to neuronal stress resilience and metabolic adaptations (Pelleymounter et al. 1995). Our results are consistent with a metabolic-neurotrophic-sympathetic interaction between leptin, BDNF and noradrenaline (Wang et al. 2020), exhibiting greater stress-sensitivity in FC. Stress reduced FC BDNF in both diet groups, whereas co-morbid CS and a WD was necessary to reduce BDNF in HPC, similar to prior reports (Macedo et al. 2015). Other studies suggest BDNF may mediate anorexigenic effects of appetite regulators, including leptin, ghrelin and insulin (Rosas-Vargas et al. 2011). Stress-related reductions in BDNF may thus have varied influences, including altered metabolism and satiety, and reduced neuronal resilience and plasticity. Proteome analysis supports an inhibitory influence of CS on synaptogenesis in both diet groups, although adaptive changes may arise in reelin and other pathways.

### Stress-dependent mitochondrial respiratory dysfunction is pronounced in FC

There is increasing attention to mitochondrial dysfunction in stress-related disorders (Ciubuc-Batcu et al. 2024; Klinedinst and Regenold 2015). Here, CS inhibited most measures of FC mitochondrial respiration, whereas the WD alone had limited impact. Somewhat unexpectedly, imposition of stress in WD mice largely failed to further worsen respiratory function, although higher cytochrome *c* dependent respiration suggests reduced membrane integrity. Conversely, CS-dependent reductions in ETS and spare respiratory capacities were not evident in WD mice. While this might be interpreted as reflecting benefit from the WD in stressed animals, reductions in select respiratory elements with CS may serve to limit mitochondrial stress (Filipović and Turck 2025). This WD effect evident only in CS + WD mice may thus be maladaptive, consistent with our hypothesis regarding impaired adaptive changes under co-morbid conditions. Mitochondrial function in hypothalamus and nucleus accumbens appear even less responsive to stress and diet, confirming a heightened sensitivity of FC respiration.

Respiratory function changes are generally congruent with proteome analysis, with the mitochondrial (dys)function path a prominent CS-sensitive process exhibiting greater sensitivity in FC *vs.* HPC. A mix of potentially adaptive and detrimental responses again emerges with CS or a WD alone: proteome analysis not only supports CS-dependent suppression of CI-III and ATP-synthase in FC, but beneficial influences of CS or a WD on molecular determinants of oxidative stress, apoptosis, and mitochondrial biogenesis, membrane integrity, mPTP activity and fission. Similar adaptive changes are evident in HPC, although TP53/BCL signaling may be enhanced. Crucially, co-morbid CS and a WD may not only reduce ATP-synthase expression/activity and CI and CIII activities, but inhibit or reverse these beneficial changes in nuclear signaling (Fig. 5, Table 1).

Impaired cortical CI respiration has been reported in rat (Zhao et al. 2023) and mouse (Santos-Silva et al. 2023) stress models. Transcriptomic analysis in the latter identified changes in mitochondrial pathways, including decreased expression of ETS components. There is also evidence a single restraint episode can inhibit CI respiration in rat forebrain (Couturier et al. 2016). Others report stress-dependent mitochondrial dysfunction in diverse animal models (Gong et al. 2011; Grigoruţă et al. 2020), together with evidence of adaptive responses (Nold et al. 2019).

Distinct from CS, the WD alone had limited independent influence on mitochondrial function, contrasting outcomes with more extreme dietary modification and obesity (de Mello et al. 2019; Kelty et al. 2023; Langley et al. 2020; Tyagi et al. 2018). However, there are also reports of limited (Couturier et al. 2016) or even adaptive effects of such extreme diets (Crescenzo et al. 2019; Lord et al. 2021). Our results appear consistent with limited respiratory effects of diets with less than ~ 40% of calories from fat, and a WD-related shift in fatty acid dependent ATP generation. Apparently adaptive influences of the WD alone on determinants of oxidative stress and mitochondrial biogenesis also meshes with increased transcription or expression of anti-oxidants and biogenesis mediators in the HPC of pigs fed a high-fat diet (Kelty et al. 2023). Importantly, and similar to findings for FC neurochemistry, the WD alone had no independent effect on either FC or HPC mitochondrial function yet influenced ETS and spare respiratory capacities and CII FCR in FC, and CII and CI + CII FCRs in HPC. These observations are again consistent with greater neurobiological impacts of the WD under co-morbid conditions.

In prior proteomic profiling, Filipović and colleagues identify remodeling of synaptic and non-synaptic mitochondria in the HPC of stressed rats (Filipovic et al. 2024; Filipović and Turck 2025). While reporting differing responses in sub-groups defined as “resilient” or “non-resilient”, this categorization was based only on sucrose preference with no other behavioral or basic phenotypic measures (e.g. body weight, metabolic or endocrine markers) assessed. Despite this major limitation, findings suggest preservation of hedonic behavior may involve stress-related increases in pyruvate dehydrogenase (PDH) and ATP-synthase components and reductions in CI and CIII components, together with relatively higher TCA cycle, chaperone/quality-control and anti-oxidant protein levels. Such changes are theorized to enhance bioenergetic capacity and mitochondrial maintenance, and limit oxidative stress. Nonetheless, and as in the present study, causal involvement of mitochondrial protein changes remains to be established. That said, the CS-dependent inhibition of FC CI and CIII and activation of CMA (important in mitochondrial maintenance) here is consistent with these reported HPC changes (Filipović and Turck 2025). While co-morbid CS and a WD also reduced CI and CIII activities, ATP-synthase expression was impaired and beneficial shifts in CMA and nuclear pathways governing oxidative stress, apoptosis, biogenesis, membrane integrity, mPTP activity and fission appear to be inhibited or reversed under co-morbid conditions (Fig. 5, Table 3). Overall, these proteome profiles are consistent with a detrimental impact of co-morbid CS and a WD, potentially reducing bioenergetic capacity, mitochondrial defenses and quality control.

### A WD and CS induce both “adaptive” and “maladaptive” pathway responses

While proteomic analysis suggests CS may impair mitochondrial and synaptogenesis pathways, it also provides evidence of adaptive pathway responses to CS or a WD alone. These include enhanced CMA quality control, reelin-dependent nerve growth/development and EIF2-dependent defense signaling. Importantly, these adaptive responses to stress appear to be absent or reversed in mice also fed a WD. Such regulatory transitions with co-morbid CS and a WD, coupled with suppression of neuroprotective pathways (Table 3), are predicted to increase CNS vulnerability.

#### Chaperone-mediated autophagy

The WD and CS are predicted to activate CMA in FC and HPC, whereas co-morbidity appears to blunt the CMA response in FC. Chaperone-mediated autophagy is essential in protein quality control and homeostasis. Oxidative and other stressors trigger adaptive CMA activation, protecting cells by degrading oxidized proteins and limiting ROS accumulation (Le et al. 2022). Selectivity of CMA supports protective roles during nutritional stress (Madrigal-Matute et al. 2022), and in limiting chronic disease development and aging (Cuervo and Wong 2014). This mode of autophagy is particularly important in neurons due to their complex architecture, long lifespan, and inability to dilute aggregate load via cell division. Recent proteome analysis of prefrontal cortex supports improved quality control via CMA and the proteasome system in adaptation to stress in rats (Filipovic et al. 2024). Importantly, proteome data here suggests a transition from such beneficial CMA pathway activation with CS or a WD to a detrimental pathway inhibition under co-morbid conditions.

#### Synaptogenesis

Mechanisms of stress-related MDD ultimately converge on the synapse (Fries et al. 2023), and recent proteome analysis associates synapse-related proteins with stress-resilience in rats (Filipovic et al. 2024). Our data suggests CS may inhibit synaptogenesis pathways in both FC and HPC, whereas the WD appears to have select stimulatory effects. The predicted influences of stress (including inhibition of SNARE complex formation, and PI3K, AKT, CREB and mTOR activities) may inhibit microtubule stabilization, synaptic spine density and actin filament branching. Curiously, effects of a WD alone generally oppose these CS-dependent changes, though SNARE complex formation appears inhibited by a WD, and CRK, CRKL, CAMK2 and DLG4 expression were all increased. The latter change may be neuroprotective (Gao et al. 2023). Regulation of SNARE complex assembly is crucial to synaptic integrity (Davletov et al. 2007), and links between dietary lipids and SNARE proteins have been reported (Darios and Davletov 2006). Differential influences of CS on CDK5 (down) and WASF1 (up) in WD mice are also of interest. Under normal conditions, CDK5-dependent phosphorylation of WASF1 inhibits its ability to regulate actin polymerization (Kim et al. 2006). A differential shift in CDK5/WASF1 is thus likely to disturb this balance, influencing neuronal migration and neurite outgrowth.

#### Reelin signaling in FC

Reelin is also key to neuronal migration and synaptogenesis, and influences GABA/glutamate signaling (Faini et al. 2021). Impaired reelin signaling is linked to synaptic dysfunction and altered cortical excitability (Tissir and Goffinet 2003), and is implicated in brain aging, MDD and psychosis (Caruncho et al. 2016; Tueting et al. 1999). Here, CS or a WD are predicted to individually activate the reelin path in FC, consistent with evidence nutritional stress alters reelin expression in brain (Roberts et al. 2019), and particularly FC (Labouesse et al. 2017). Such activation may facilitate energetic homeostasis (Sabbir et al. 2021) and improve cyto-architectural outcomes (Cheerathodi and Ballif 2011). Importantly, the WD appears to inhibit (or reverse) reelin pathway changes with CS, consistent with a transition from adaptation to detriment with co-morbid CS and a WD.

#### NRF2 and EIF2 stress response pathways

The NRF2 response is a critical defense, controlling expression of > 200 proteins to limit oxidative damage (Morris et al. 2019). The apparent inability of CS to independently modify this defense path in FC suggests minimal oxidant generation and/or CS-dependent suppression of NRF2 pathway sensitivity. The ability of CS to inhibit the pathway in HPC is consistent with the latter, suggesting a mechanism by which stress might sensitize the CNS to injury. Inhibition of the NRF2 response by the WD is consistent with reported reductions in NRF2 activity (Morrison et al. 2010) and increases in oxidative damage (Tan and Norhaizan 2019) with elevated dietary fats.

The “integrated stress response”—in which phospho-regulation of EIF2 optimizes stress adaptation processes—was also activated by CS. This adaptive response also appears to be prevented in WD mice. Several responses to CS interpreted as neuroprotective or adaptive thus appear to be inhibited in WD mice, identifying multiple mechanisms by which co-morbid CS and a WD may promote CNS vulnerability (Table 3).

### Study limitations

Experimentation was undertaken in male mice, thus potential sex differences are not considered. Females may have different metabolic and physiological responses to WD feeding (Murtaj et al. 2022) and psychological stress (Helman et al. 2022a, b). In this initial analysis, our primary focus was to identify effects and interactions between 2 major disease risks in a detailed integrative manner. Further work will be necessary to test whether social and metabolic stressors induce similar or distinct neurobiological and behavioral outcomes in females.

A second limitation relates to assessment of anhedonia in studies involving altered palatability of food/drink (noted above). Increased dietary fats trigger an energy homeostasis imbalance which may inhibit hedonic feeding and interfere with the capacity of sucrose preference to broadly assess anhedonia. That said, a recent study shows a high fat diet produces similar changes in depressive-like behaviors assessed via diet-dependent (*eg*. sucrose preference) and independent (*eg*. forced swim) tests (Tsai et al. 2022). Future work should nonetheless include additional measures to more effectively test depressive behaviors. Assessment of cognitive disturbances would also be of value.

Finally, we do not report on corticosterone levels in this study, thus cannot conclude definitively that the social stress model represents a relatively mild form of chronic stress. Although we have shown this mode of stress does not elevate resting corticosterone levels at the end of stress protocol (Helman et al. 2022a,b), this is consistent with observations that chronic stress-dependent elevations in corticosterone decline over time (e.g. Pérez-Tejada et al. 2013; Yalcin et al. 2011). Analysis of acute corticosterone reactivity in additional studies might provide useful information regarding stress severity. Nonetheless, absence of anhedonia and moderate changes in open field behavior are consistent with a relatively modest stressor.

### Conclusions

The present study demonstrates that CS and a WD impact neurobiological processes in a region specific manner, with apparently heightened vulnerability in FC *vs.* HPC. Stress significantly disrupts mitochondrial respiratory function and associated pathways in FC. Moderate CS or a WD also induce adaptive pathway changes, however their co-morbidity may overwhelm the neurobiological system, potentially reflecting additive influences on shared biological mechanisms and transitions in feedback control (Stapelberg et al. 2018, 2019). While we do not establish causal involvement in behavioral disturbances, as with the studies of Filipovic and others correlating neurobiological and behavioral measures (Ates et al. 2014; (Flipovic et al. 2024; Filipović, & Turck 2025; Francis et al. 2022; Griffith et al. 2022; Morrison et al. 2010; Pérez-Tejada et al. 2013; Santos-Silva et al. 2023; Zhao et al. 2023), these exploratory analyses identify rational candidates for targeted mechanistic (and therapeutic) investigation. Findings here lend support to mitochondrial involvement, while changes in several adaptive pathway responses under co-morbid conditions (Table 3) are consistent with the conceptual model of critical transitions in networks governing neurologic vulnerability and the development of stress-related disorders (Stapelberg et al. 2018, 2019).

## Supplementary Information

Below is the link to the electronic supplementary material.

Supplementary file1 (PDF 3460 KB)

## Data Availability

All data supporting the findings of this study are available in the paper and **Supplement** file, with the proteomics datasets available via ProteomeXchange with the identifier PXD07418, and raw respirometery and behavioral data available from the authors upon reasonable request.

## Declarations

### Declaration of AI use

The authors declare that no generative AI or AI-assisted technologies were used in the preparation of this manuscript.

## Abbreviations

AMPA: α-Amino-3-hydroxy-5-methyl-4-isoxazolepropionic acid
AMPK: AMP-activated protein kinase
APP: Amyloid precursor protein
BBC3: BCL2 binding component 3
BCL: BCL2 binding component
BDNF: Brain-derived neurotrophic factor
BMAL1: Basic helix-loop-helix ARNT like 1
BRAF: B-Raf proto-oncogene, serine/threonine kinase
CaMK2: Calcium/calmodulin-dependent protein kinase
CD: Control diet
CI: Mitochondrial Complex I
CII: Mitochondrial Complex II
CIII: Mitochondrial Complex III
CIV: Mitochondrial Complex IV
CDK: Cyclin-dependent kinase
CMA: Chaperone-mediated autophagy
CREB: CAMP response element-binding protein
CRK: Proto-oncogene c-Crk
CYCS: Cytochrome c, somatic
DEP: Differentially expressed protein
DLG4: Post-synaptic density protein 95
EIF2: Eukaryotic initiation factor 2
ERK: Extracellular signal-regulated kinase
ETS: Electron transport system
FC: Frontal cortex
FCR: Flux control ratio
GABA: Gamma-aminobutyric acid
GPCR: G-protein coupled receptor
HOMA-IR: Homeostasis model assessment of insulin resistance
HPC: Hippocampus
JAK: Janus kinase
JNK: C-Jun N-terminal kinase
LAMP2A: Lysosome-associated membrane protein 2
LIMK1: LIM domain kinase 1
MDD: Major depressive disorder
mGluR: Metabotropic glutamate receptor
MPTP: Mitochondrial permeability transition pore
NFAT: Nuclear factor of activated T-cells
NO: Nitric oxide
NRF: Nuclear factor erythroid 2-related factor
OFT: Open field test
p70s6k: 70 Ribosomal S6 kinase
PAC1: Pituitary adenylate cyclase-activating polypeptide type I receptor
PKA: Protein kinase A
POU5F: POU domain, Class 5, transcription factor
PPAR-γ: Peroxisome proliferator-activated receptor gamma
PPARGC1α: PPAR-g coactivator 1 alpha
RAC1: Rac family small GTPase 1
RAF1: Raf-1 proto-oncogene, serine/threonine kinase
RAP: Receptor associated protein
RARA: Retinoic acid receptor alpha
RHOA: Ras homolog family member A
ROS: Reactive oxygen species
ROX: Residual O_2_ consumption
SAPK: Stress-activated protein kinase
SIRT: Sirtuin
SNARE: Soluble N-ethylmaleimide-sensitive factor attachment protein receptor
SOX2: SRY-related HMG-box gene 2
SPT: Sucrose preference test
STAT: Signal transducers and activators of transcription
TCA: Tricarboxylic acid
TFAM: Transcription factor A, mitochondrial
TP53: Tumor protein p53
VDAC: Voltage-dependent anion channel
WASF1: Wiskott–Aldrich syndrome protein family member 1
WD: Western diet
