# Supplementary material for: Neurobiology of co-morbid stress and a western diet in mice: mitochondrial, proteomic and behavioral outcomes
Source: Metab Brain Dis. 2026 Apr 27;41(1):94. doi: 10.1007/s11011-026-01855-3 (PMC13121405; doi:10.1007/s11011-026-01855-3)

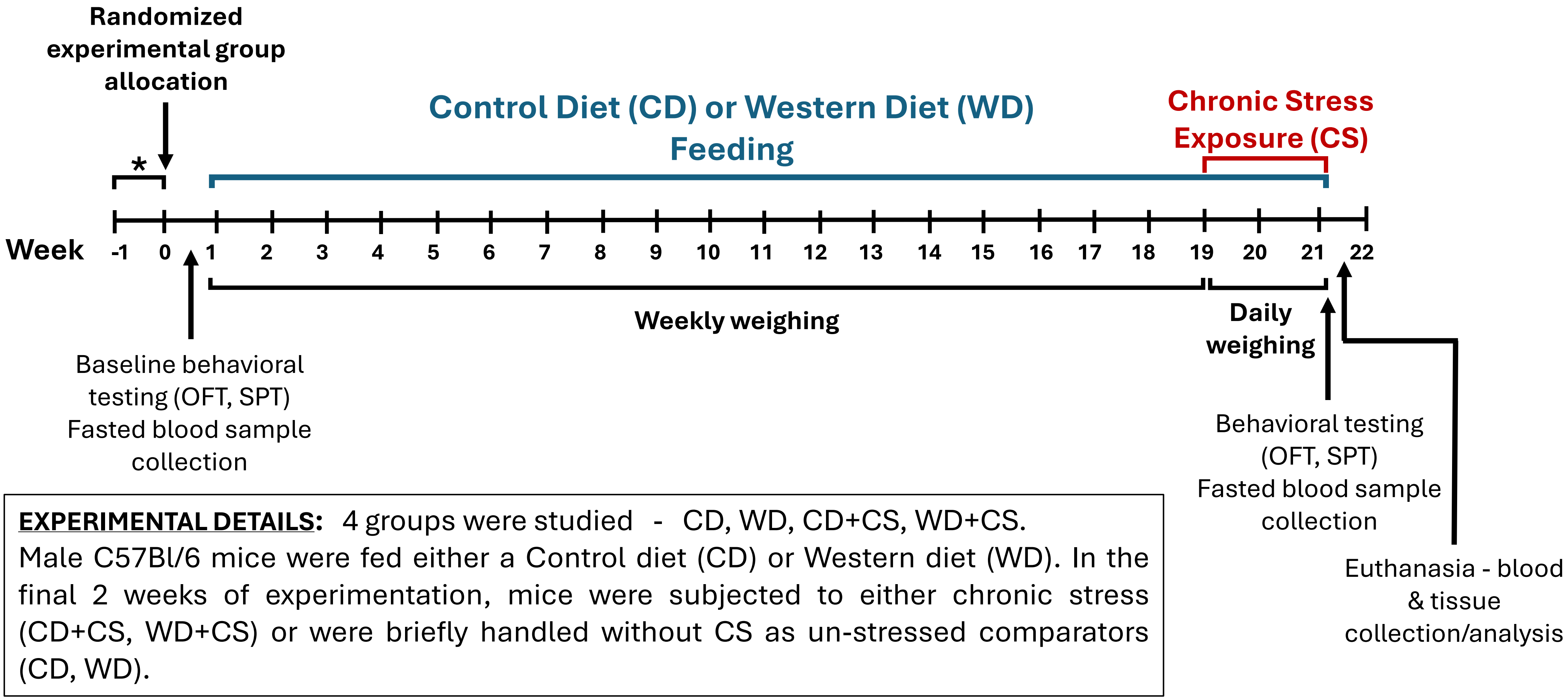

<u>EXPERIMENTAL DETAILS</u>: 4 groups were studied - CD, WD, CD+CS, WD+CS.
Male C57Bl/6 mice were fed either a Control diet (CD) or Western diet (WD). In the final 2 weeks of experimentation, mice were subjected to either chronic stress (CD+CS, WD+CS) or were briefly handled without CS as un-stressed comparators (CD, WD).

Table S1: Select mitochondrial parameters and their calculation.

Measure / Description	Calculation
Sole cytochrome c respiration (Cyt c Respiration<br>Change in respiratory flux on addition of exogenous cytochrome c; measure of outer mitochondrial membrane integrity	Respiration after cytochrome c - Respiration before cytochrome c
Leak consumption ratio (LCR)<br>Leak respiration is uncoupled/non-phosphorylating respiration - proton and electron leak in presence of NADH-generating substrates	Leak respiration / Maximal respiratory capacity
Spare respiratory capacity<br>Amount of additional ATP that may be produced via oxidative phosphorylation	Maximal respiratory capacity – Respiration after Complex I & II
Complex I (CI) FCR<br>CI-linked respiration relative to maximal respiration	Complex I OxPhos / Maximal respiratory capacity
Complex II (CII) FCR<br>CII-linked respiration relative to maximal respiration	[Complex I & II OxPhos - Complex I OxPhos] / Maximal respiratory capacity
Combined Complex I & II (CI+CII) FCR<br>Combined CI+CII linked respiration relative to maximal respiration	Complex I FCR + Complex II FCR

Nano-LC MS/MS Analysis of Brain Tissue

Sample volumes equivalent to 50 µg protein were transferred to chilled tubes and MilliQ Ultrapure Water (Merck-Millipore, Bedford, USA) added to normalize loading volumes. Reduction of each sample was completed using 100 mM dithiothreitol (Bio-Rad Laboratories Inc., Hercules, USA) and 100 mM ammonium bicarbonate (Sigma Aldrich, Missouri, USA) in MilliQ water (1:1:3) at 37°C for 30 min, followed by alkylation using 200 mM iodoacetamide (Sigma Aldrich, Missouri, USA) for 15 min at room temperature. Sample pH was checked using pH indicator strips (Merck-Millipore, Bedford, USA) and adjusted to pH 7-9 where necessary. Samples were buffer exchanged on Amicon 3 kDa spin filters (Merck-Millipore, Bedford, USA) using 100 mM ammonium bicarbonate. The protein solution was digested with Sequencing Grade Modified Trypsin (Promega Corporation, Madison, USA) at 37°C for 16-18 hrs at a sample-to-trypsin ratio of 1:50. Each sample was transferred into a 1.5 mL tube with volume normalized using 10 mM ammonium bicarbonate. To stop the trypsin reaction, 1 µL of 99% formic acid was added, followed by brief vortex and centrifugation at 21,300*g* for 5 min (ambient temperature). A 2 µL un-diluted sample was transferred to a LC-MS vial and loaded onto the autosampler (maintained at 4°C).

Digested peptides were separated by nanoLC using an Ultimate3000 nano RSLC UPLC and an autosampler system (ThermoFisher, Illinois, USA). Samples were concentrated and desalted on a micro C18 pre-column (300 µm x 5 mm, Dionex) with H_2O:CH_3CN (98:2, 0.1 % TFA) at 15 µL/min. After a 4 min wash the pre-column was switched (Valco 10 port UPLC valve, Valco, Houston, TX) into line with a fritless nano column (75 µ x ~20 cm) containing C18AQ media (1.9 µm, 120 Å Dr Maisch, Ammerbuch-Entringen Germany). Peptides were eluted using a linear gradient of H_2O:CH_3CN (98:2, 0.1% formic acid) to H_2O:CH_3CN (64:36, 0.1% formic acid) at 200 nL/min over 50 min. High voltage (2000 V) was applied to low volume Titanium union (Valco) with the column oven heated to 45°C (Sonation, Biberach,

Germany) and the tip positioned ~0.5 cm from the heated capillary (T=300°C) of a QExactive Plus (Thermo Electron, Bremen, Germany) mass spectrometer. Positive ions were generated by electrospray and the QExactive operated in data-dependent acquisition mode. A survey scan of m/z 350-1750 was acquired (resolution=70,000 at m/z 200, with an accumulation target value of 1,000,000 ions) and lock-mass enabled (m/z 445.12003). Up to the 10 most abundant ions (>80,000 counts, underfill ratio 10%) with charge states >+2 and <+7 were sequentially isolated (width m/z 2.5) and fragmented by HCD (NCE = 30) with a AGC target of 100,000 ions (resolution = 17,500 at m/z 200). The m/z ratios selected for MS/MS were dynamically excluded for 30 sec.

Fig. S1. Hypothalamus mitochondrial respiration in tissue from male mice fed a control diet (CD) or Western diet (WD) for 20 wks ± chronic stress (CS) in the final 2 wks

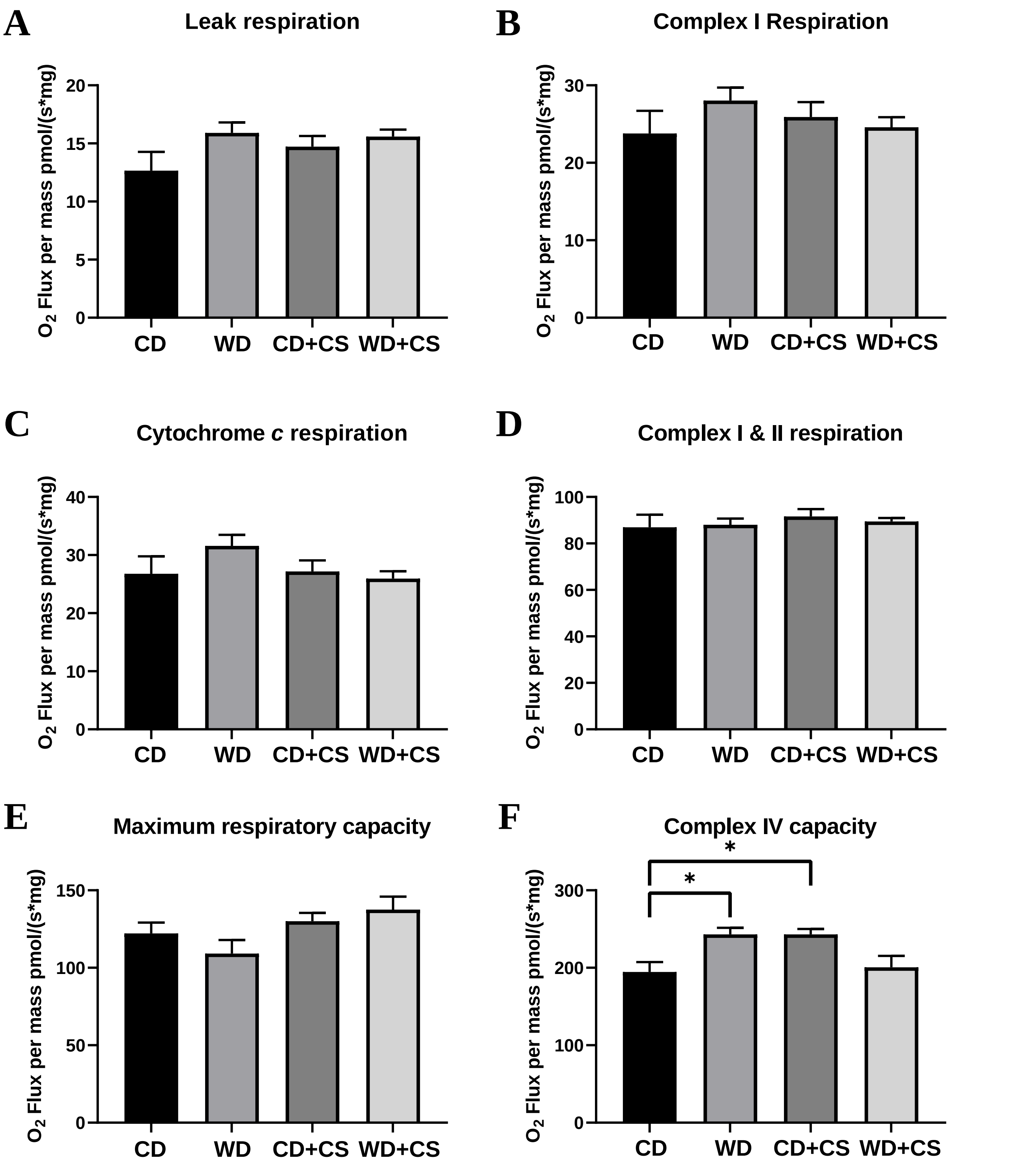

Mitochondrial respiration from mouse hypothalamus. **A)** Leak respiration. **B)** Complex I respiration. **C)** Outer Mitochondrial membrane integrity. **D)** Complex I & II linked respiration **E)** Maximum respiratory capacity. **F)** Complex IV capacity. * $p < 0.05$. Data presented as mean±SEM. n=7-8 per group. Abbreviations: CTRL, control group; WD, western diet group; CS, chronic stress group; WD + CS, western diet and chronic stress group.

Fig. S2. Hypothalamus respiratory flux control ratios in tissue from male C57Bl/6 mice subjected to CS and/or WD feeding

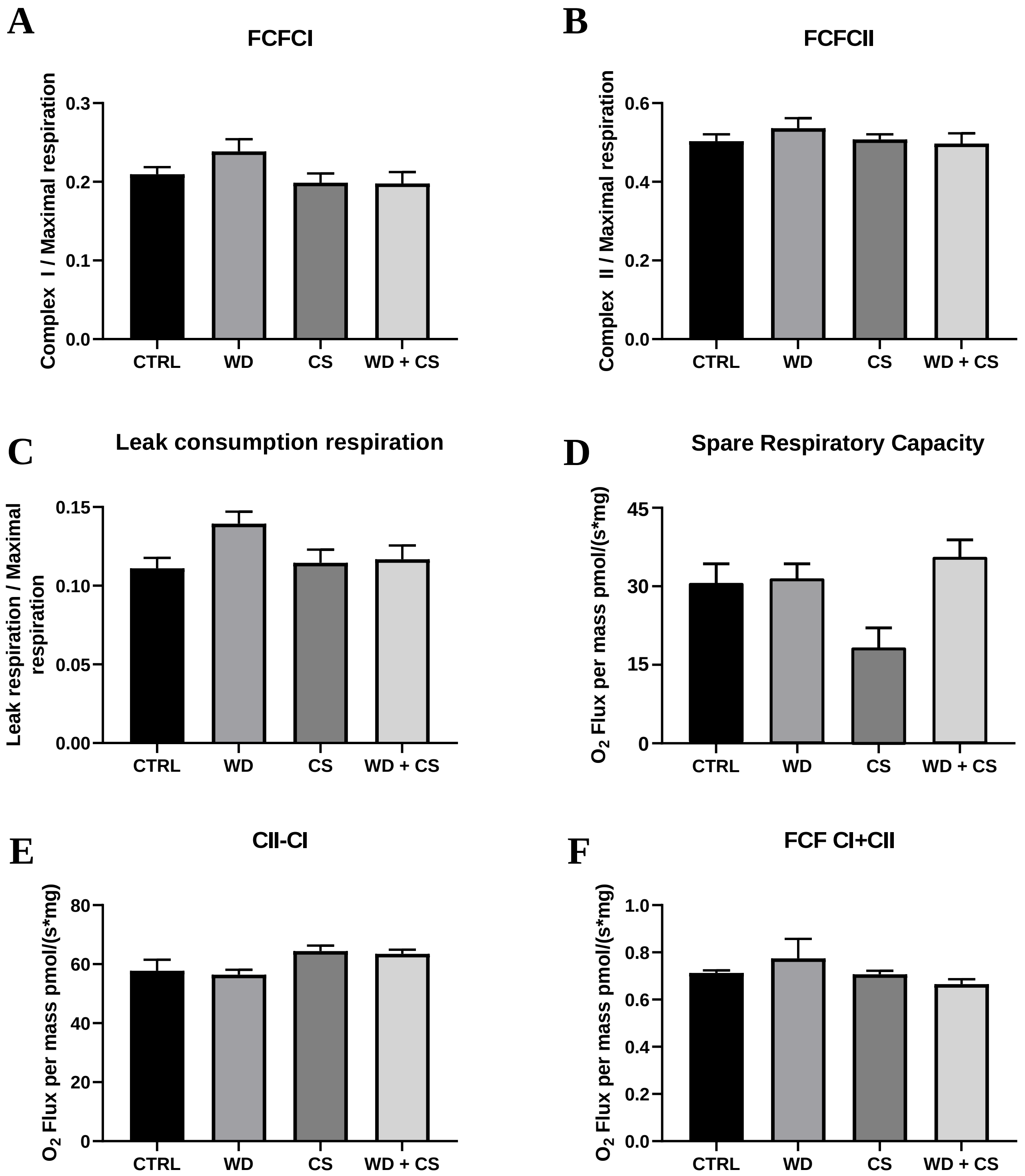

Flux control ratios for mouse hypothalamus. **A)** Flux control factor of Complex I. **B)** Flux control factor of Complex II. **C)** Flux control factor of leak respiration. **D)** Spare respiratory capacity. **E)** Complex II respiration. **F)** Flux of the sum of Complex I and CII-linked respiration. Data presented as mean± SEM. n=7-8 per group. Abbreviations: CTRL, control group; WD, western diet group; CS, chronic stress group; WD + CS, western diet and chronic stress group.

Fig. S3. Nucleus accumbens mitochondrial respiration in tissue from male mice fed a control diet (CD) or Western diet (WD) for 20 wks ± chronic stress (CS) in the final 2 wks

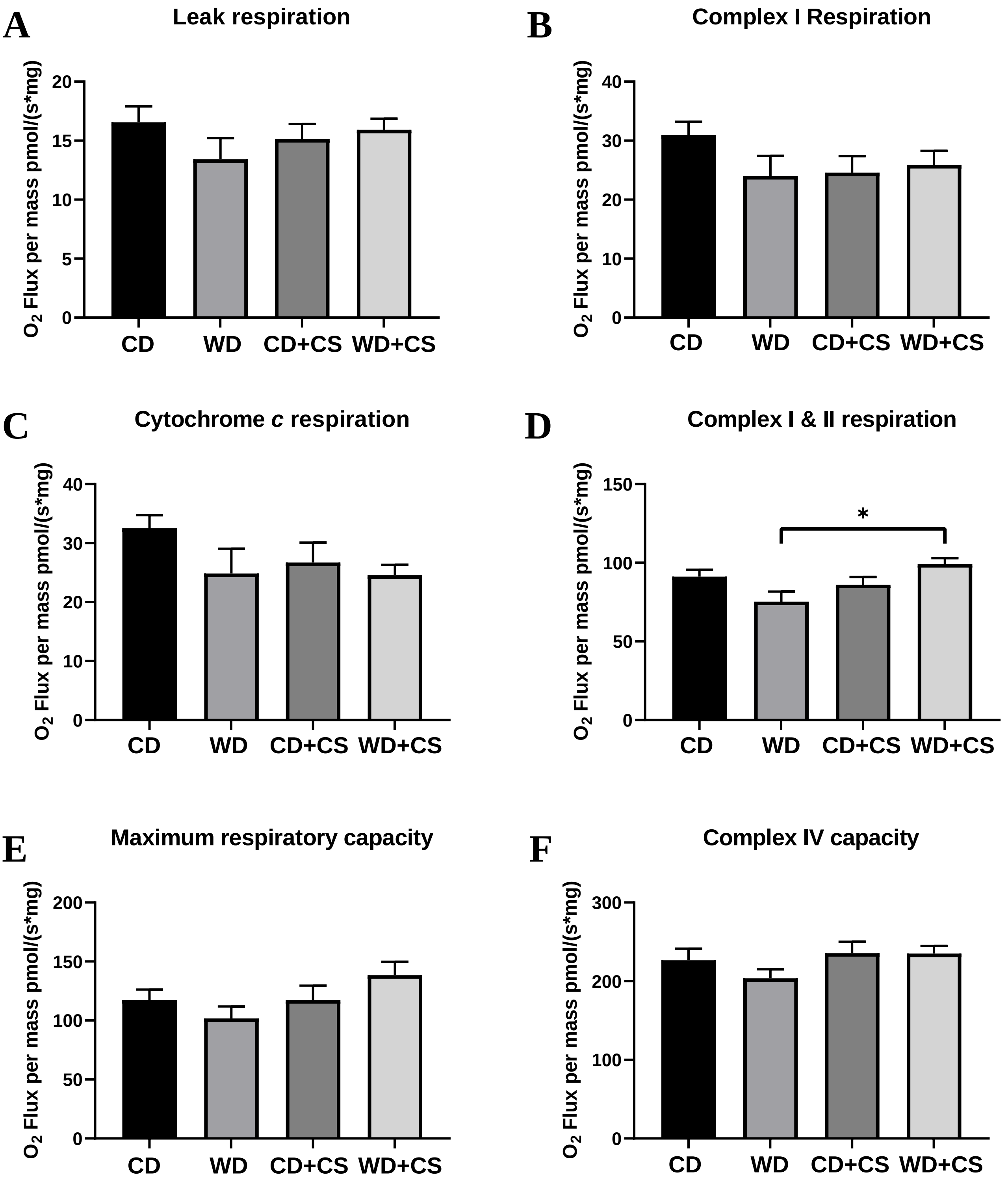

Mitochondrial respiration from mouse nuclues accumbens. **A)** Leak respiration. **B)** Complex I respiration. **C)** Outer Mitochondrial membrane integrity. **D)** Complex I & II linked respiration. * $p < 0.05$. **E)** Maximum respiratory capacity. **F)** Complex IV capacity. Data presented as mean±SEM. n=7-8 per group. Abbreviations: CTRL, control group; WD, western diet group; CS, chronic stress group; WD + CS, western diet and chronic stress group.

Fig. S4. Nucleus accumbens respiratory flux control ratios in tissue from male C57Bl/6 mice subjected to CS and/or WD feeding

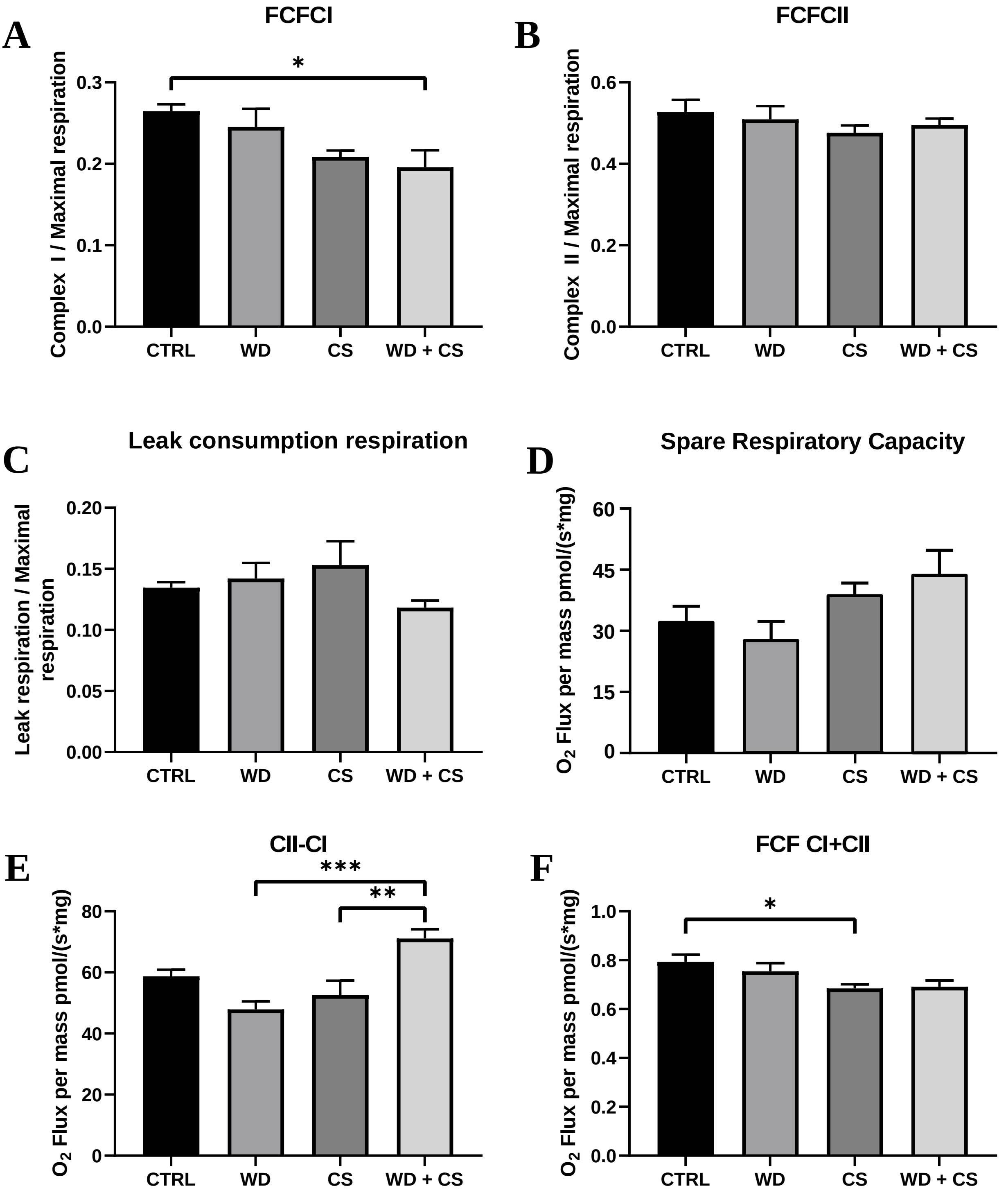

Flux control ratios for mouse nucleus accumbens. **A)** Flux control factor of Complex I. * $p < 0.05$. **B)** Flux control factor of Complex II. **C)** Flux control factor of leak respiration. **D)** Spare respiratory capacity. * $p < 0.05$. **E)** Complex II respiration. ** $p < 0.01$, *** $p < 0.0005$. **F)** Flux of the sum of Complex I and CII-linked respiration. * $p < 0.05$. Data presented as mean± SEM. n=7-8 per group. Abbreviations: CTRL, control group; WD, western diet group; CS, chronic stress group; WD + CS, western diet and chronic stress group.

Fig. S5. Proteome analysis in FC tissue: CD+CS *vs.* CD

A) Venn diagram and volcano plot for DEPs

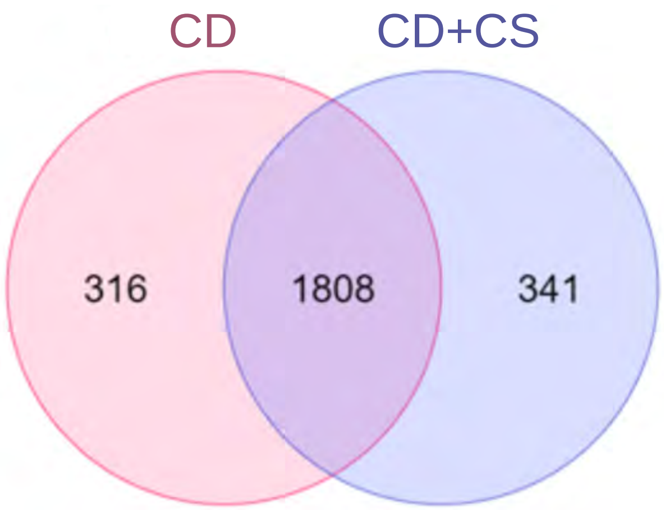

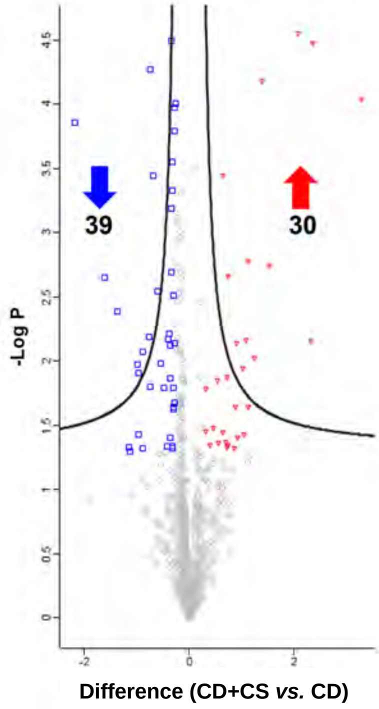

B) Cell component (GO:CC), molecular function (GO:MF) and biological processes (GO:BP)

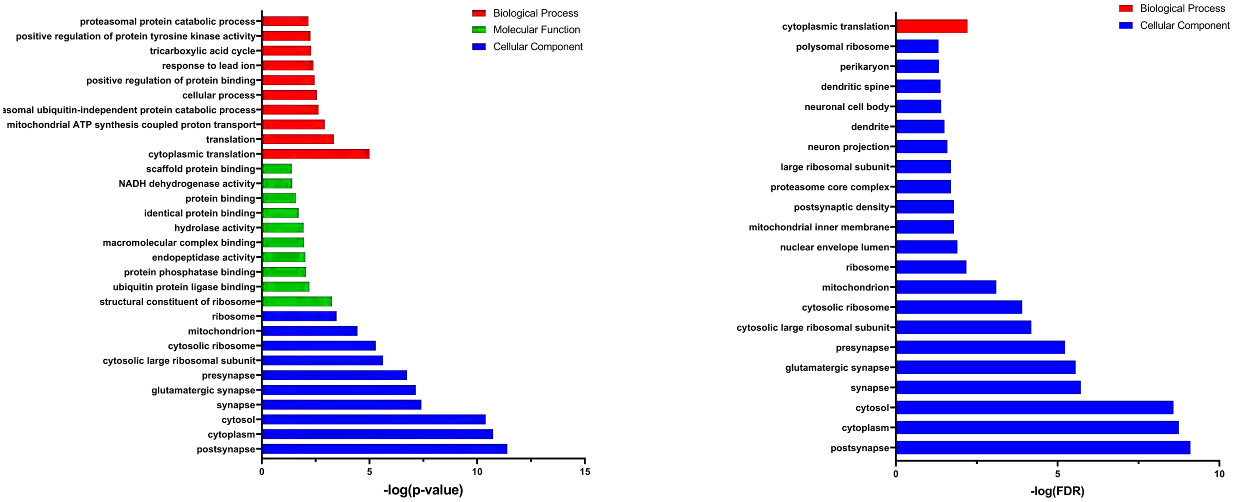

C) Modified pathways (z-score)

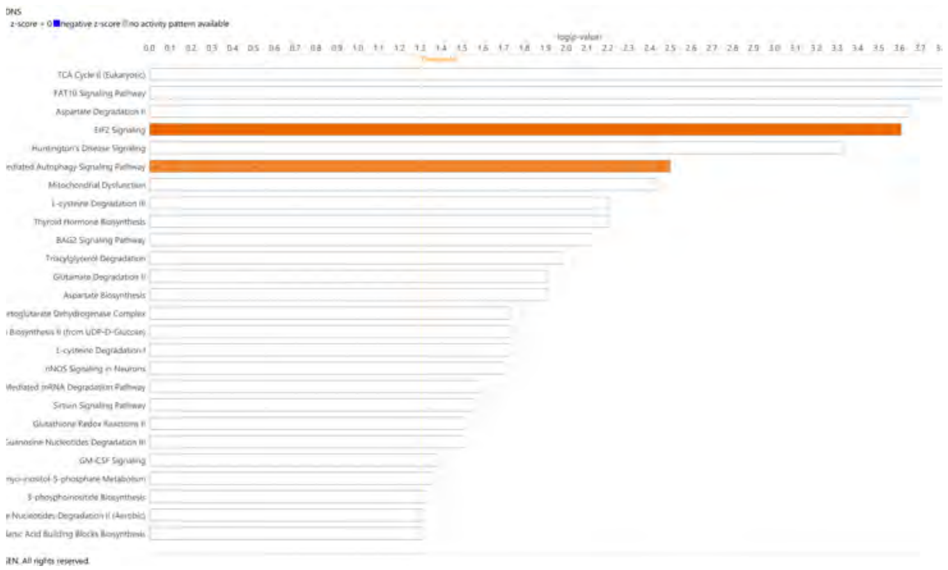

Fig. S6. Proteome analysis in FC tissue: WD *vs.* CD

A) Venn diagram and volcano plot for DEPs

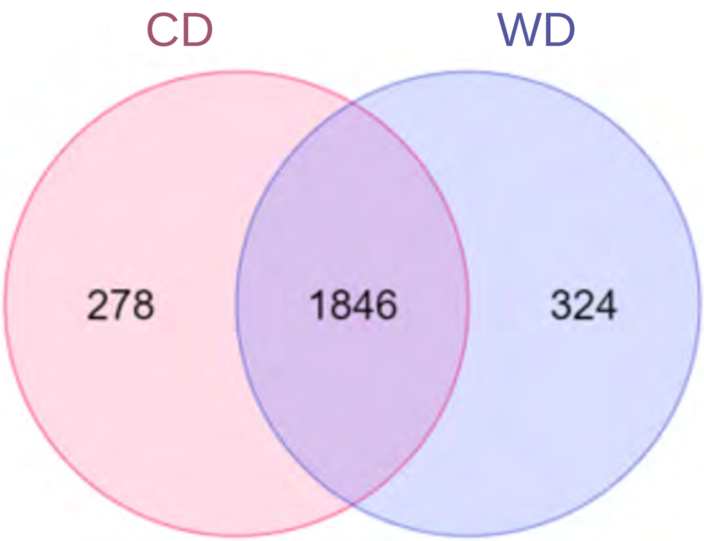

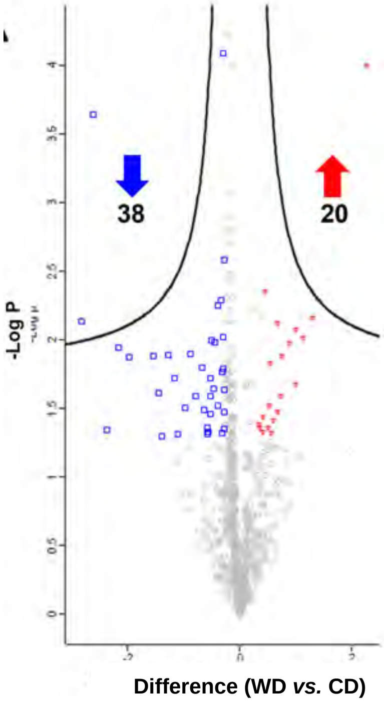

B) Cell component (GO:CC), molecular function (GO:MF) and biological processes (GO:BP)

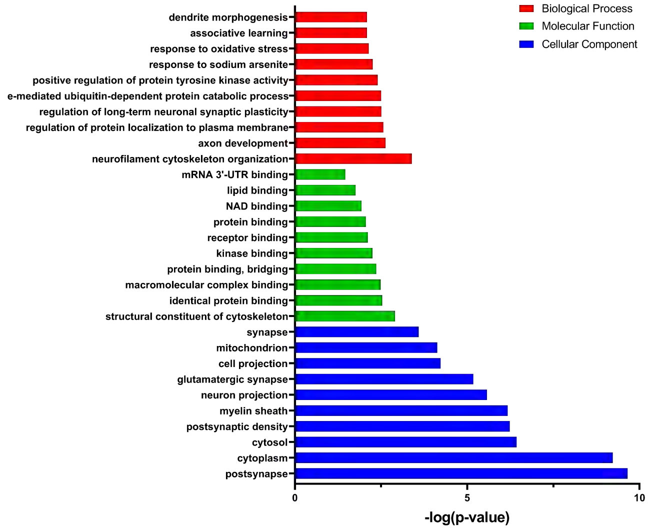

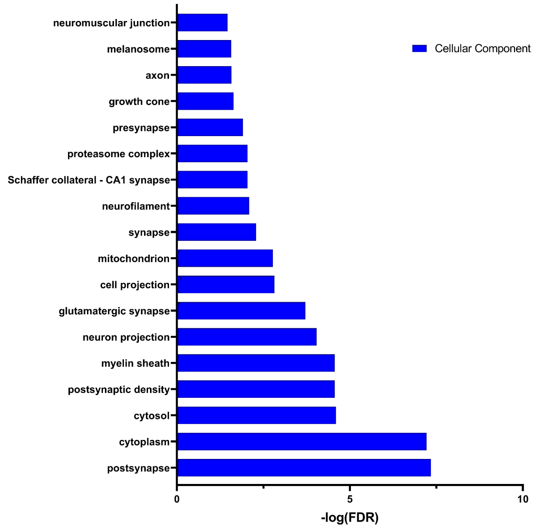

C) Modified pathways (z-score)

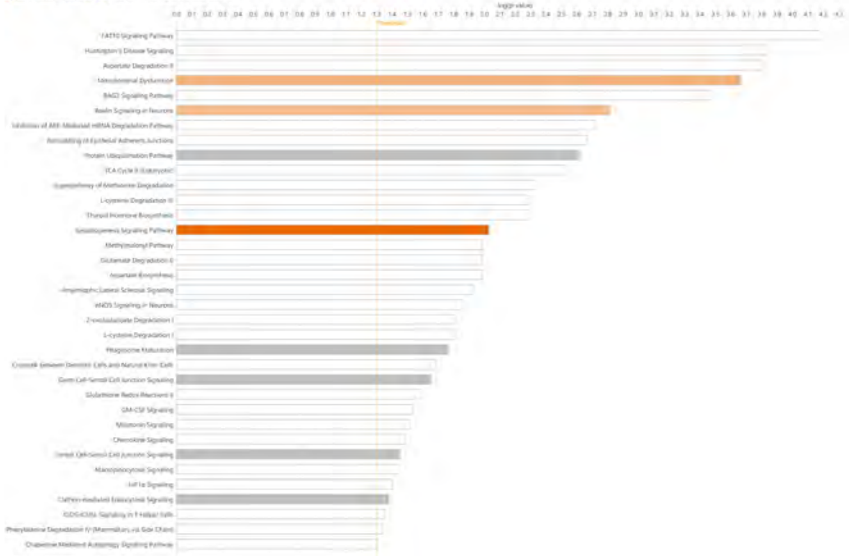

Fig. S7. Proteome analysis in FC tissue: WD+CS *vs.* WD

A) Venn diagram and volcano plot for DEPs

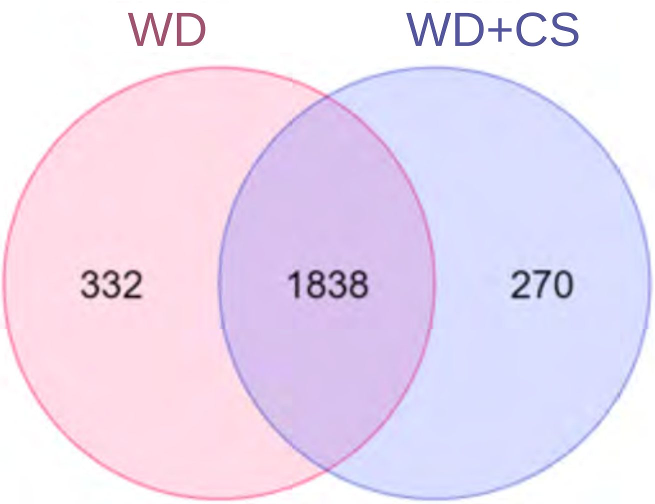

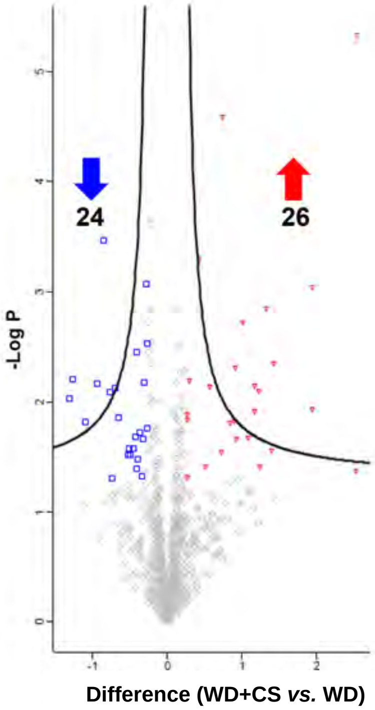

B) Cell component (GO:CC), molecular function (GO:MF) and biological processes (GO:BP)

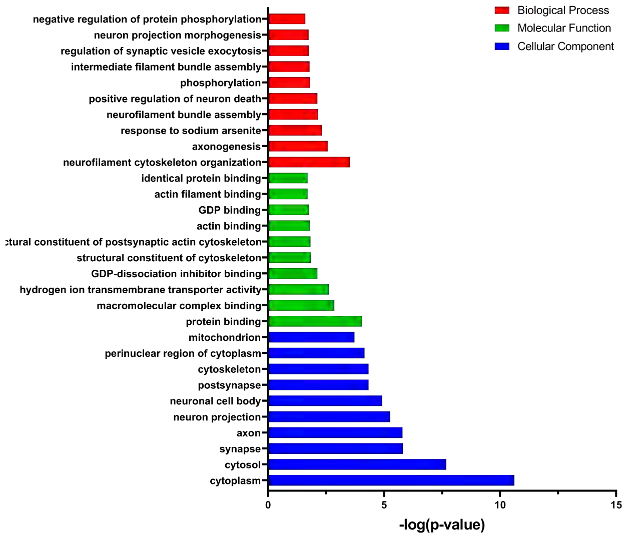

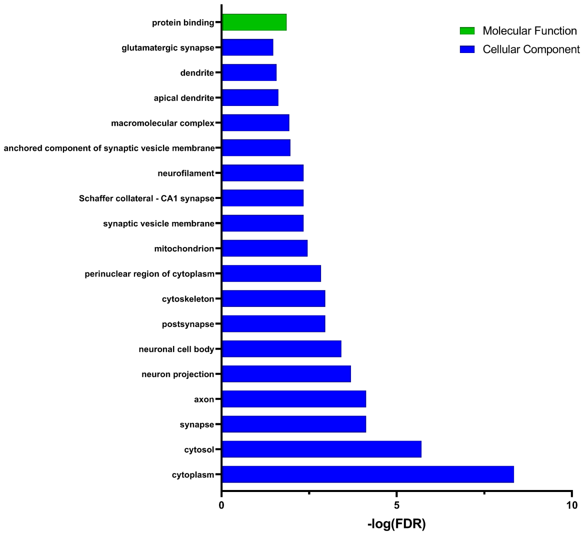

C) Modified pathways (z-score)

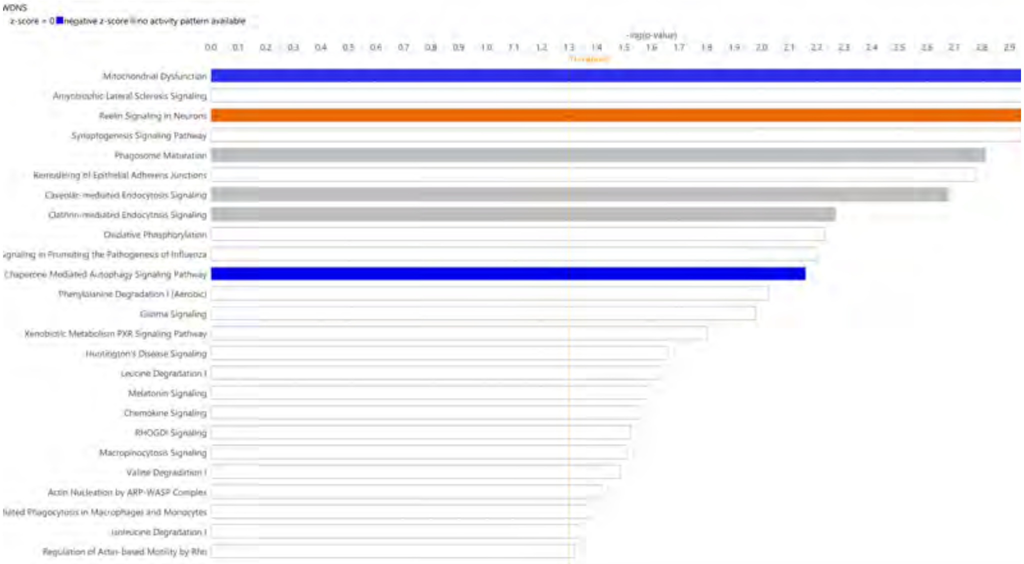

Fig. S8. Stress and Diet Sensitive Pathways in FC tissue

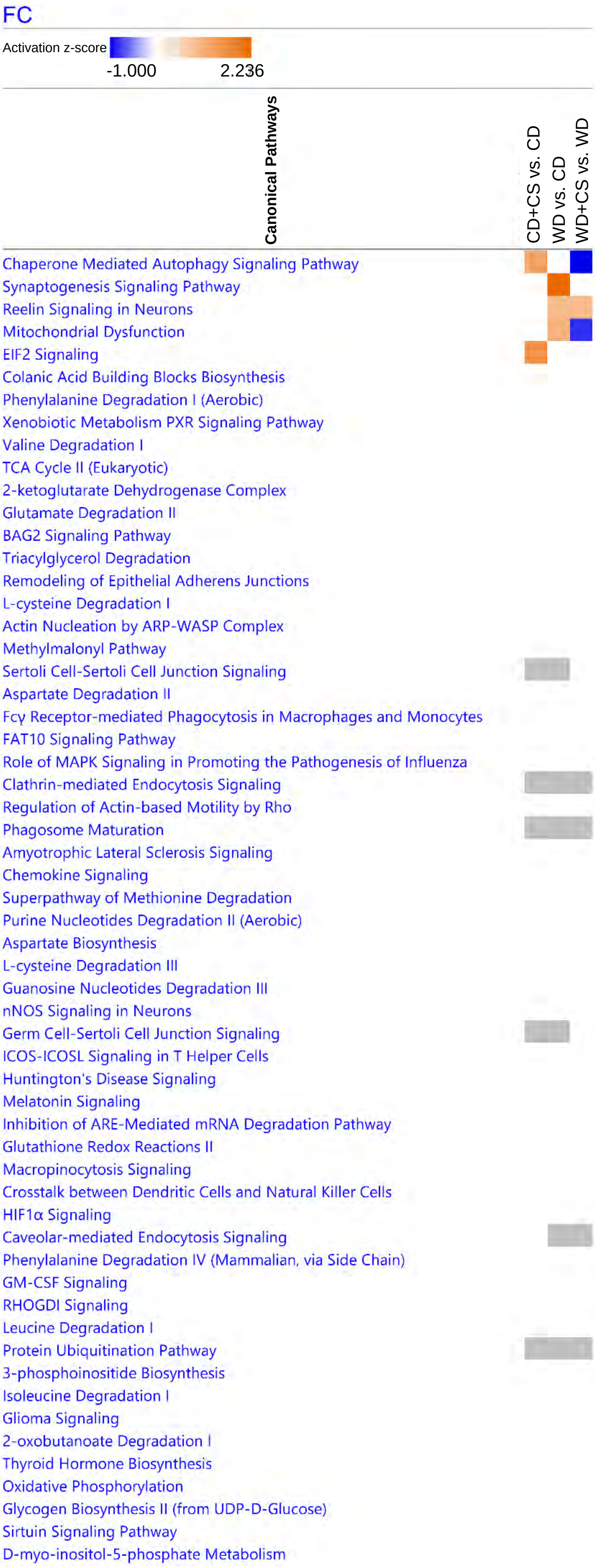

Fig. S9. Proteome analysis in HPC tissue: CD+CS *vs.* CD

A) Venn diagram and volcano plot for DEPs

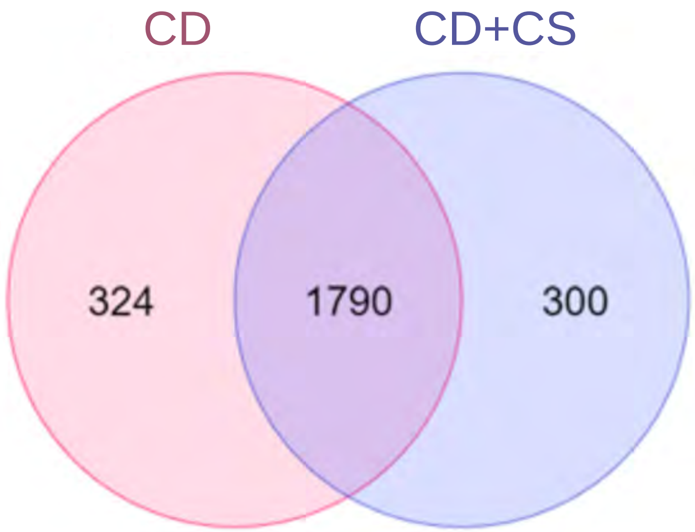

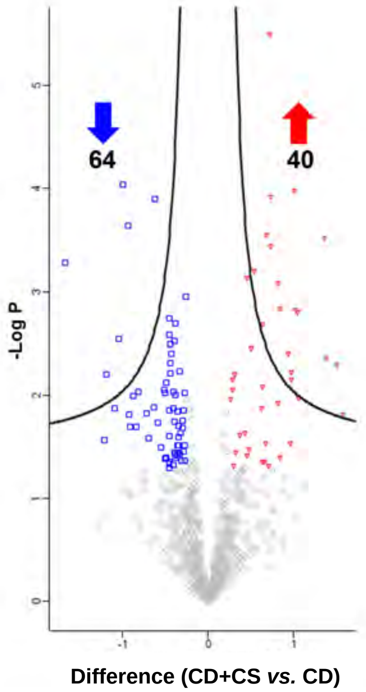

B) Cell component (GO:CC), molecular function (GO:MF) and biological processes (GO:BP)

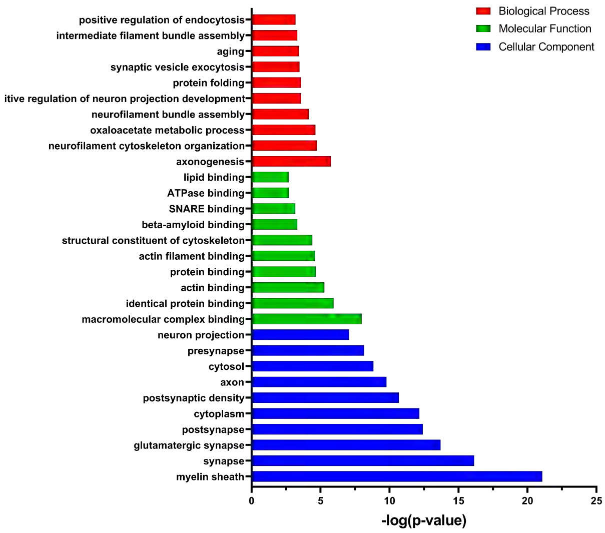

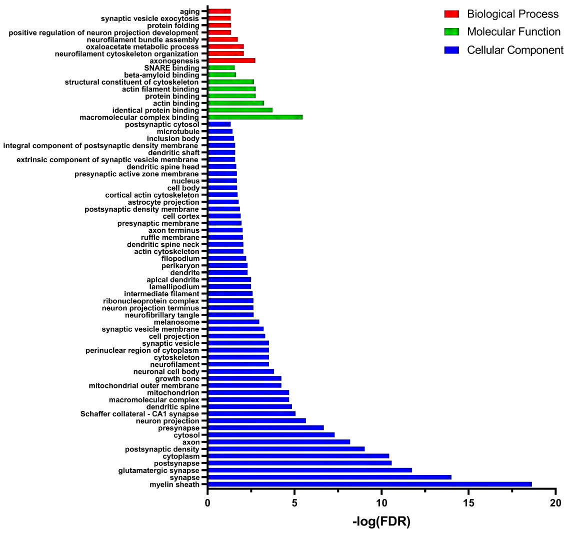

C) Modified pathways (z-score)

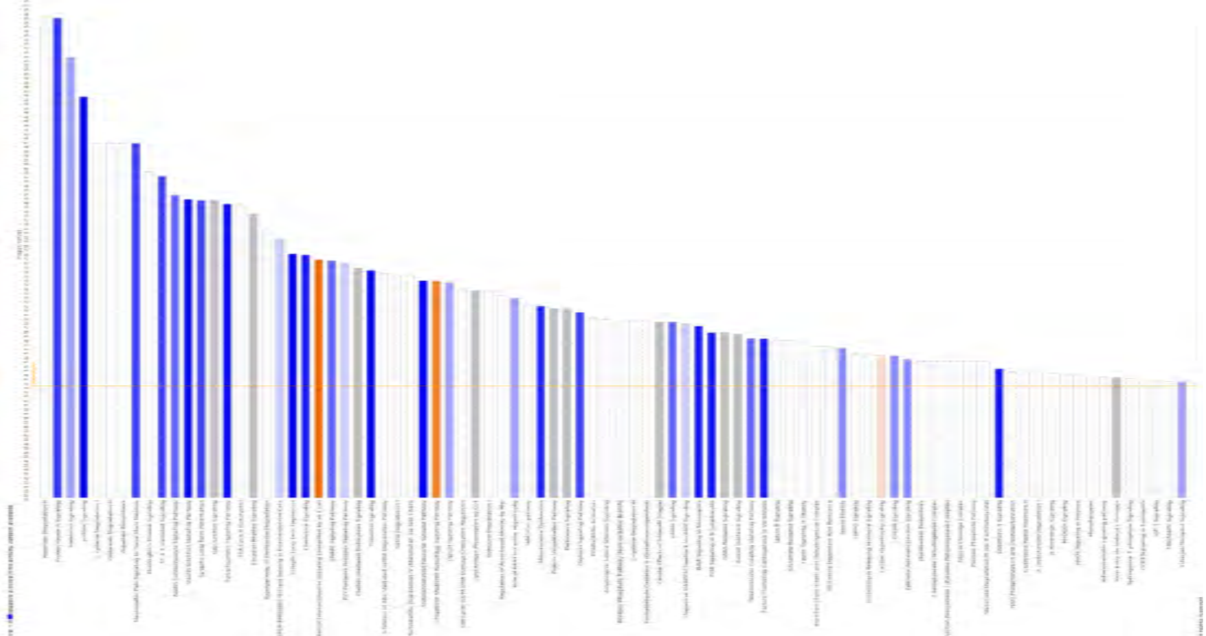

Fig. S10. Proteome analysis in HPC tissue: WD *vs.* CD

A) Venn diagram and volcano plot for DEPs

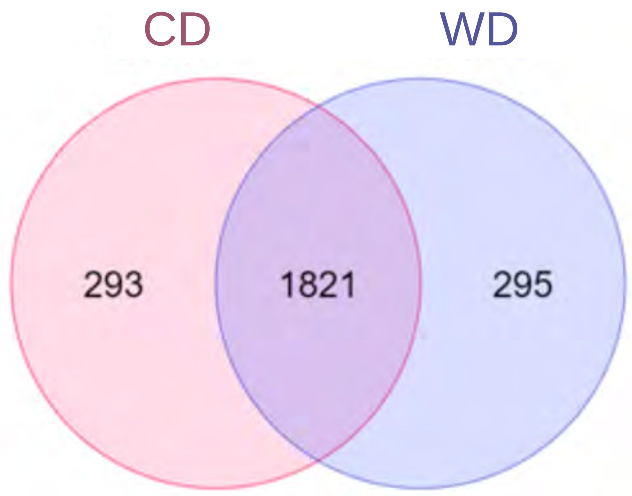

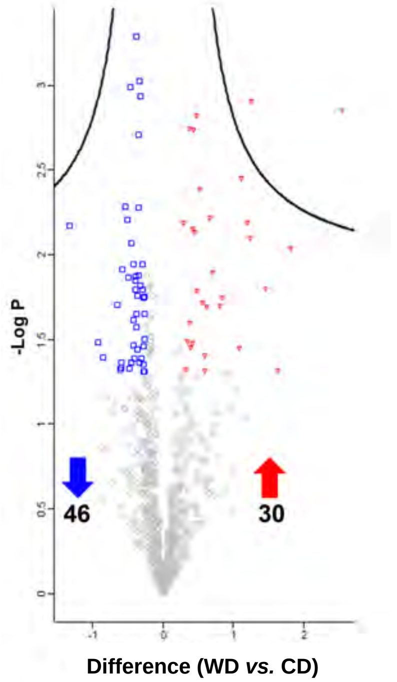

B) Cell component (GO:CC), molecular function (GO:MF) and biological processes (GO:BP)

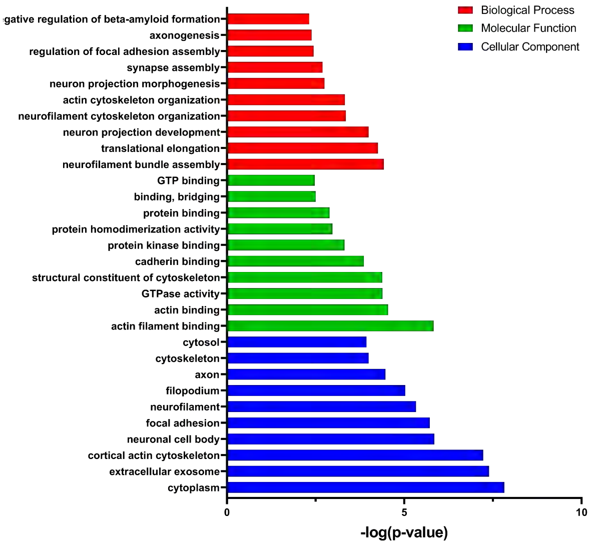

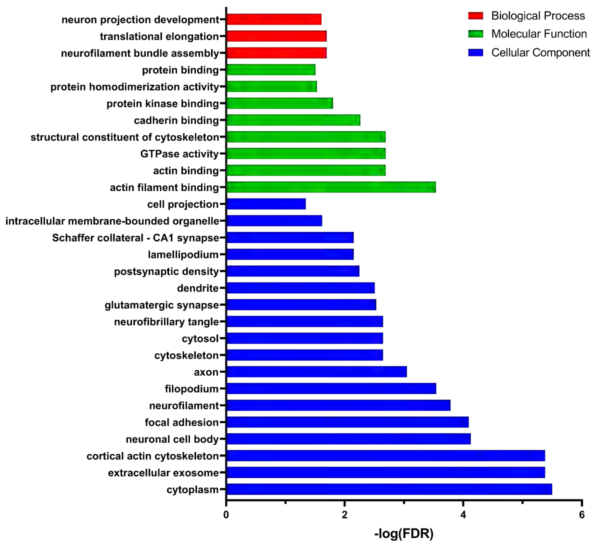

C) Modified pathways (z-score)

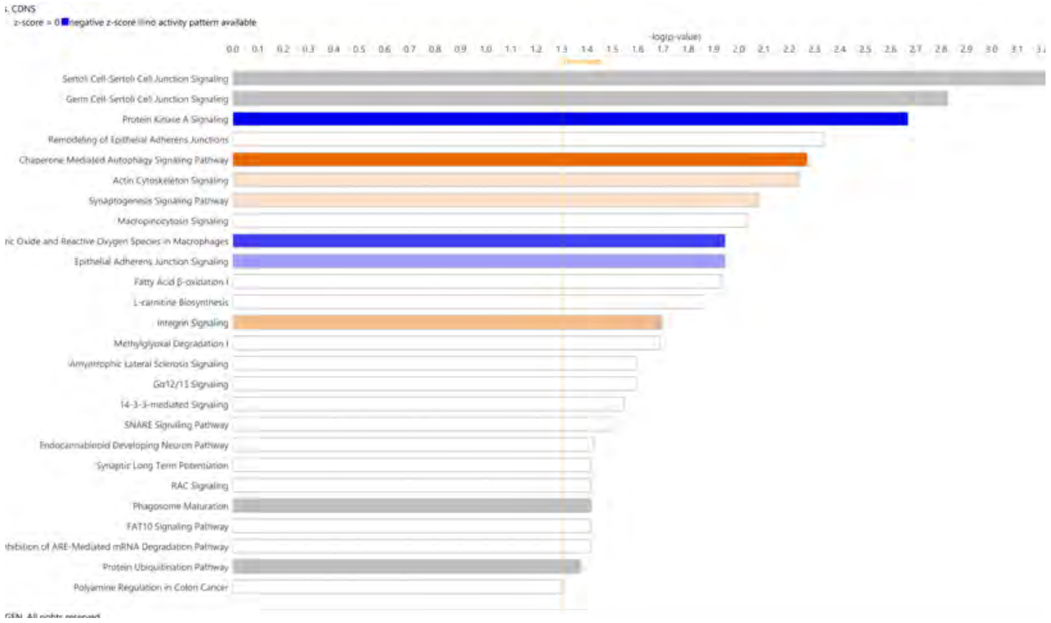

Fig. S11. Proteome analysis in HPC tissue: WD+CS *vs.* WD

A) Venn diagram and volcano plot for DEPs

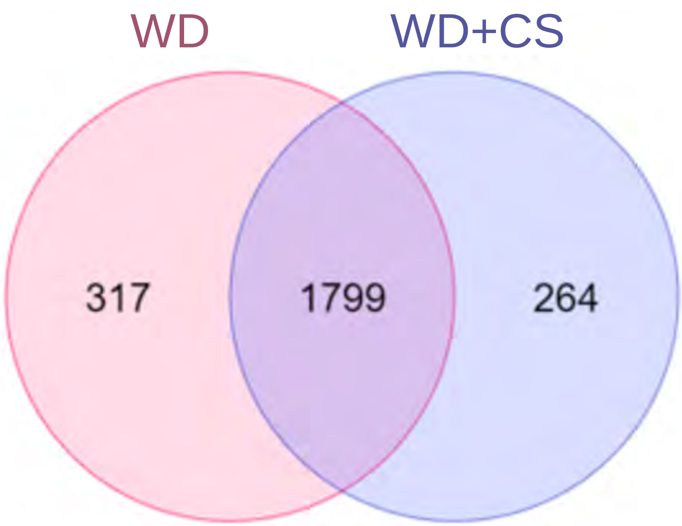

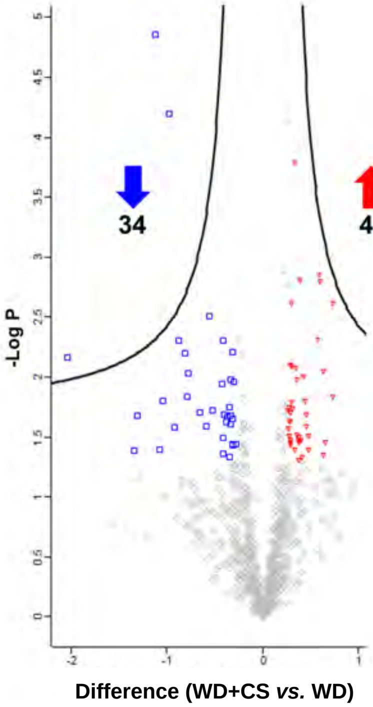

B) Cell component (GO:CC), molecular function (GO:MF) and biological processes (GO:BP)

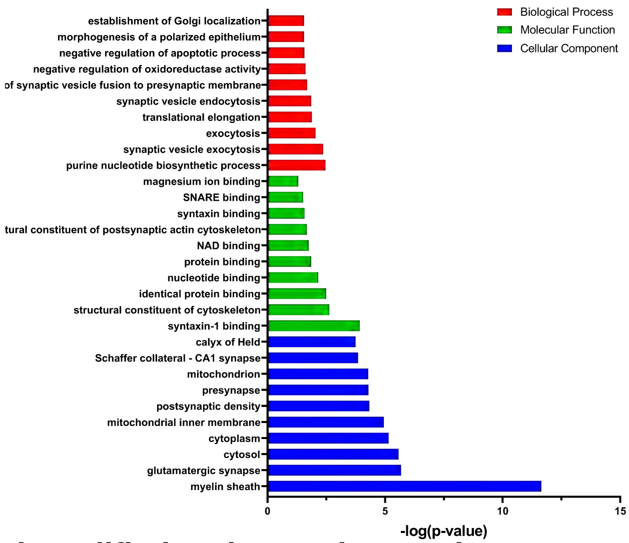

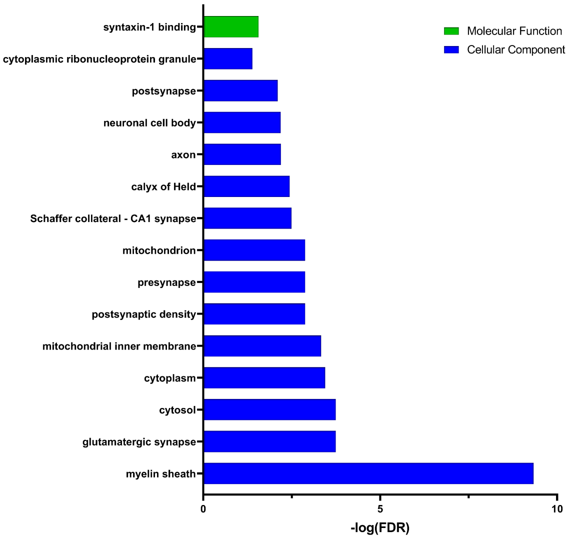

C) Modified pathways (z-score)

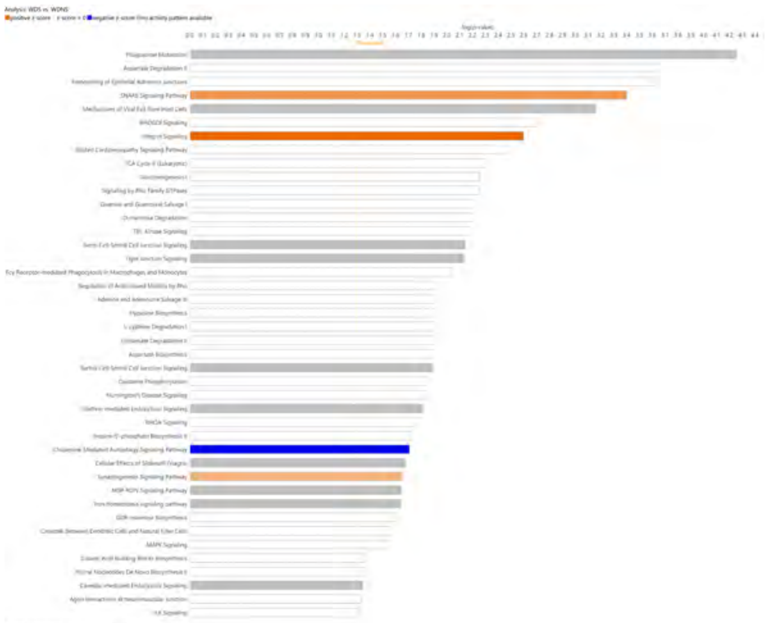

Fig. S12. Stress and Diet Sensitive Pathways in HPC tissue

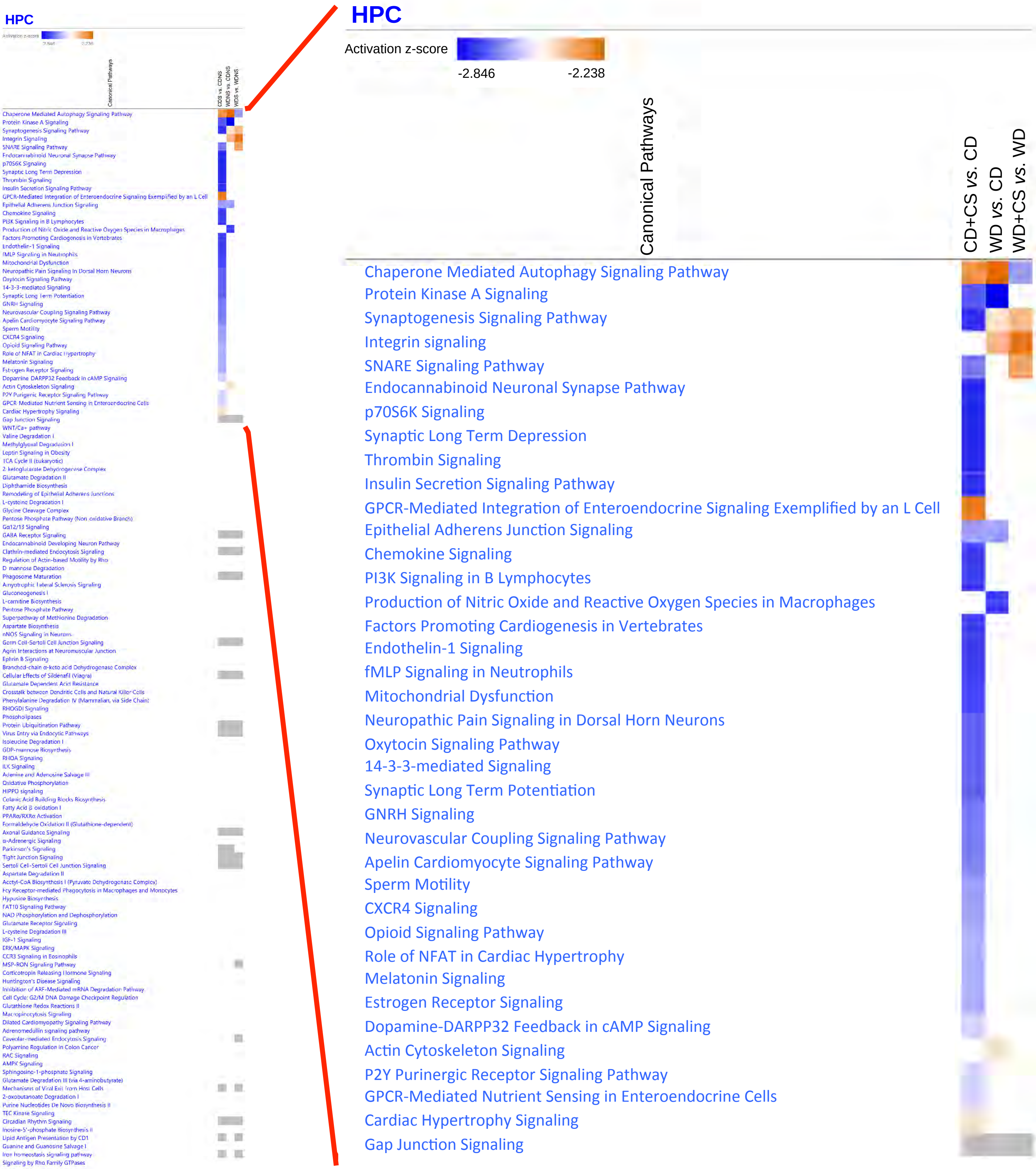

Pathway figures legend
(for Figs. S13-S21)

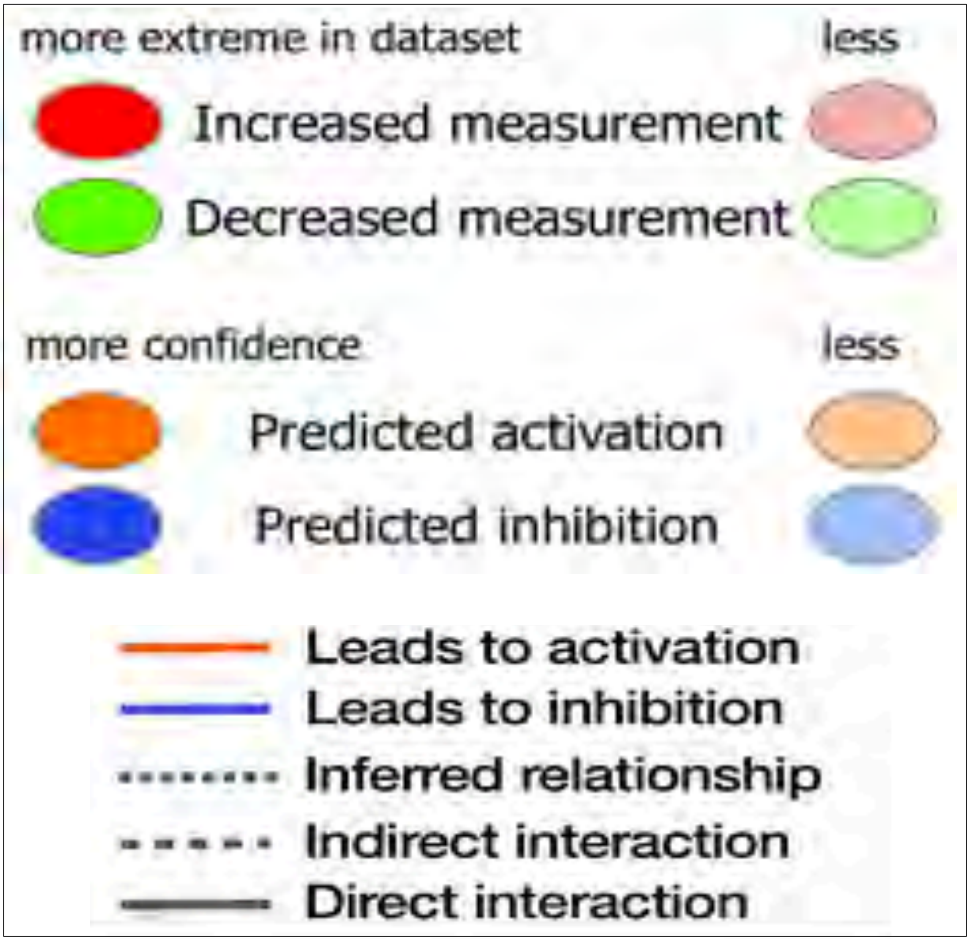

Fig. S13. Reelin Singaling Pathway Response in FC Tissue

WD *vs*. CD

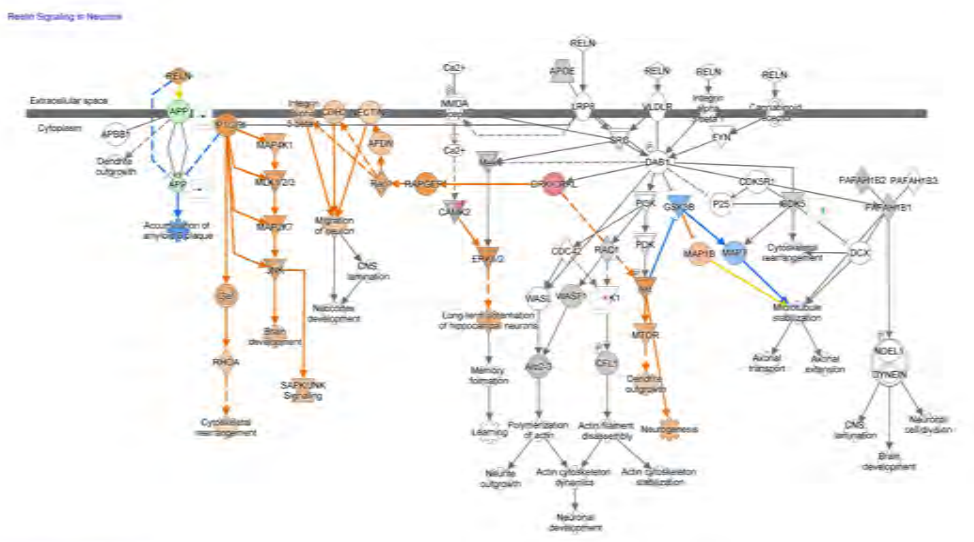

WD+CS *vs*. CD+CS

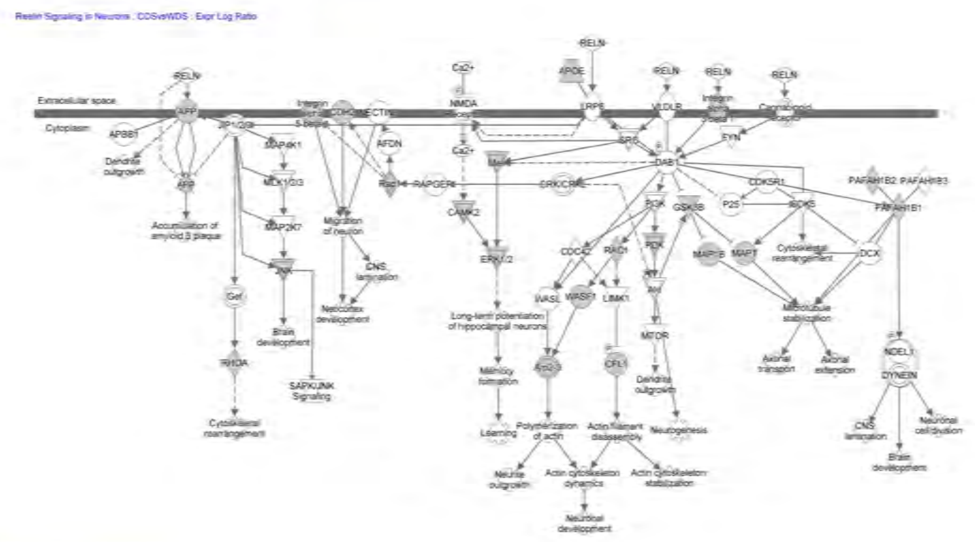

CD+CS *vs*. CD

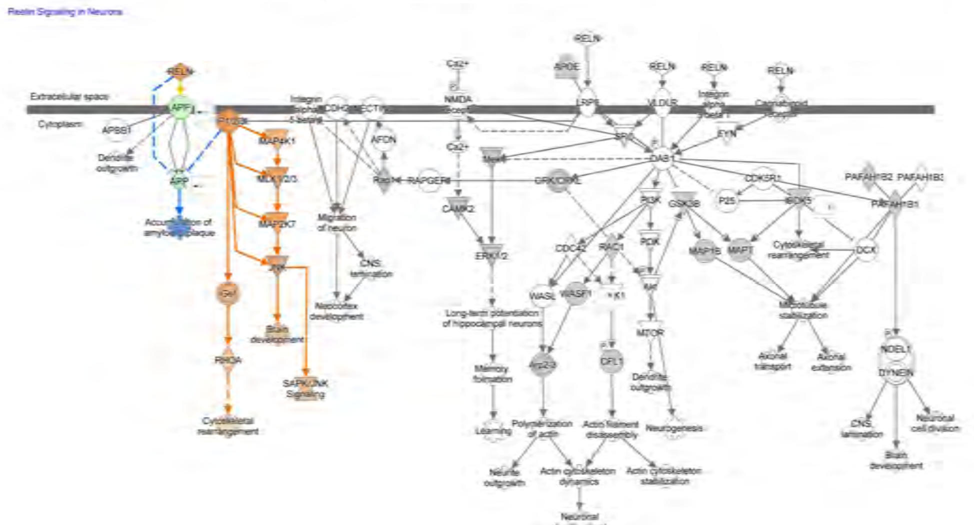

WD+CS *vs*. WD

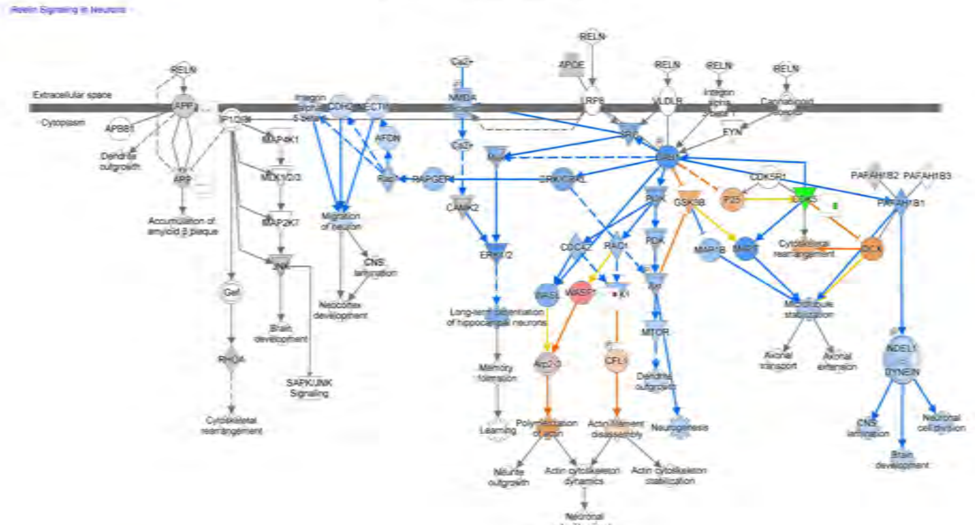

Fig. S14. NRF2 Oxidate Stress Pathway Response in FC Tissue

WD *vs*. CD

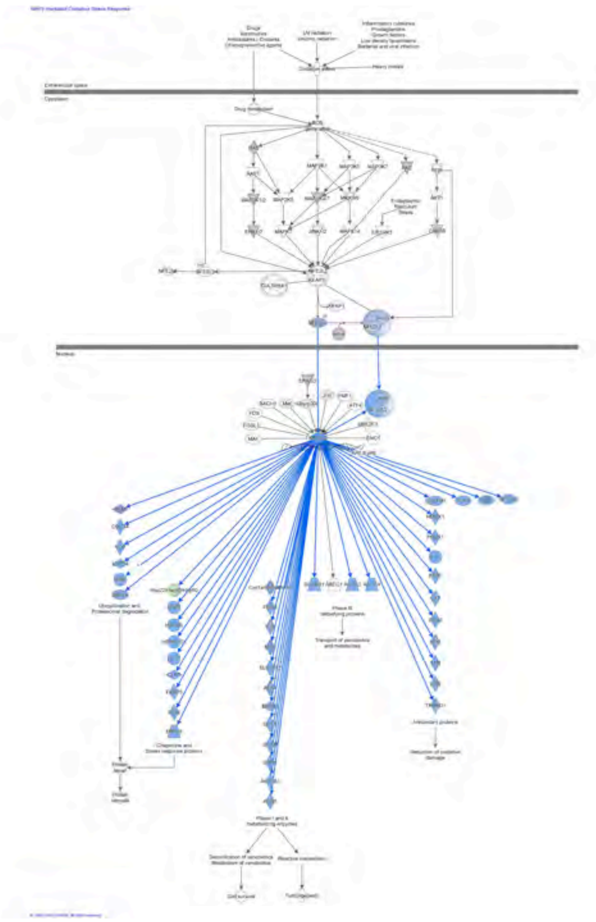

WD+CS *vs*. CD+CS

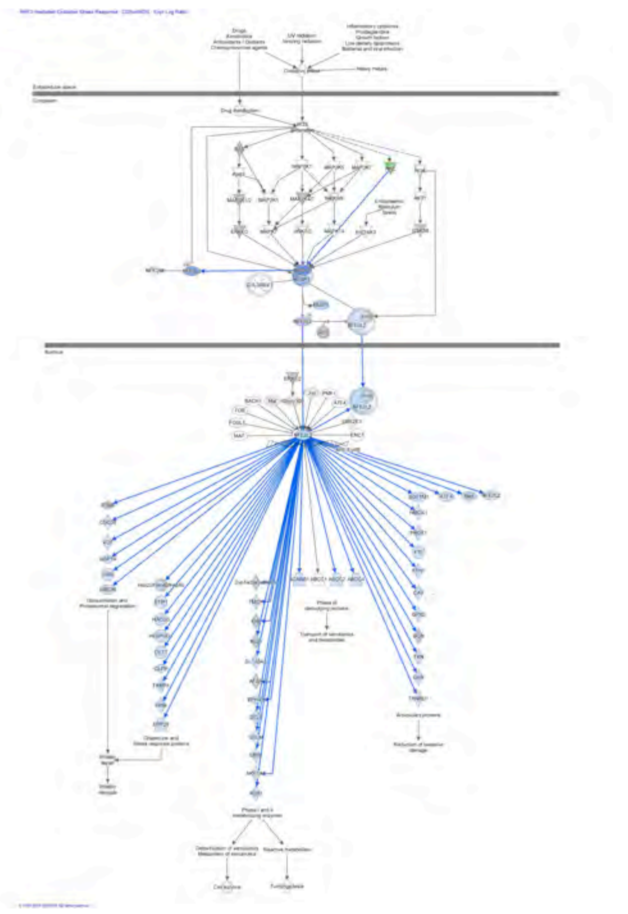

CD+CS *vs*. CD

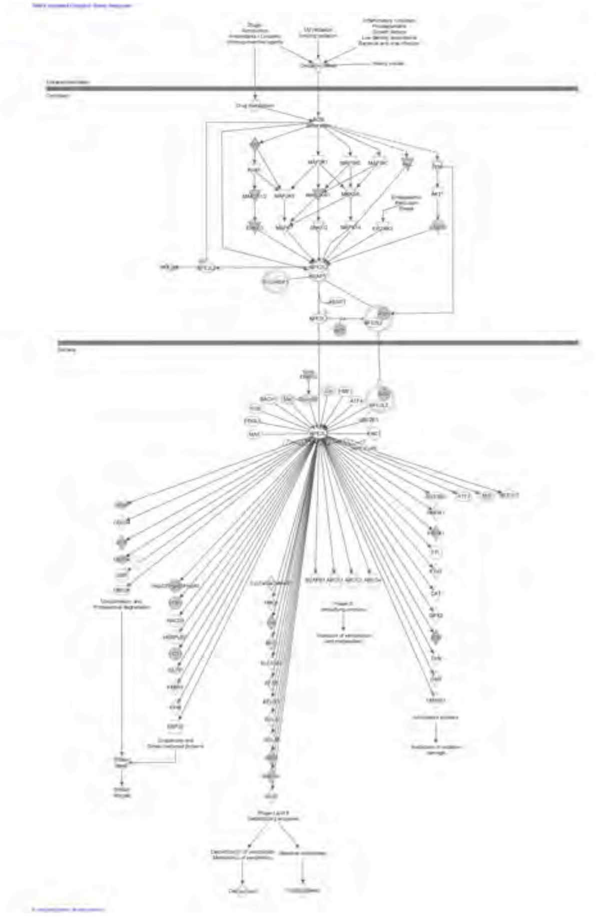

WD+CS *vs*. WD

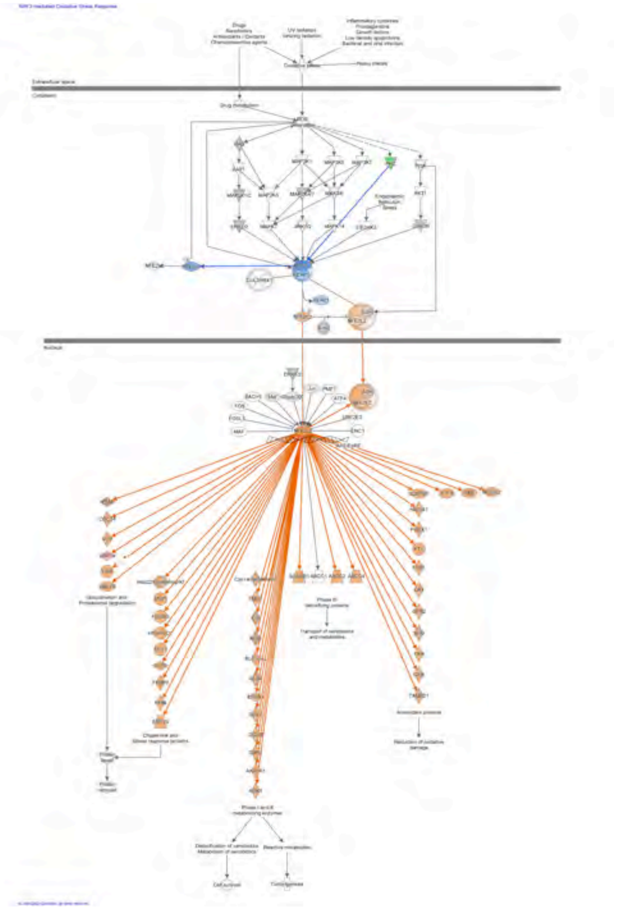

Fig. S15. EIF2 Pathway Response in FC Tissue

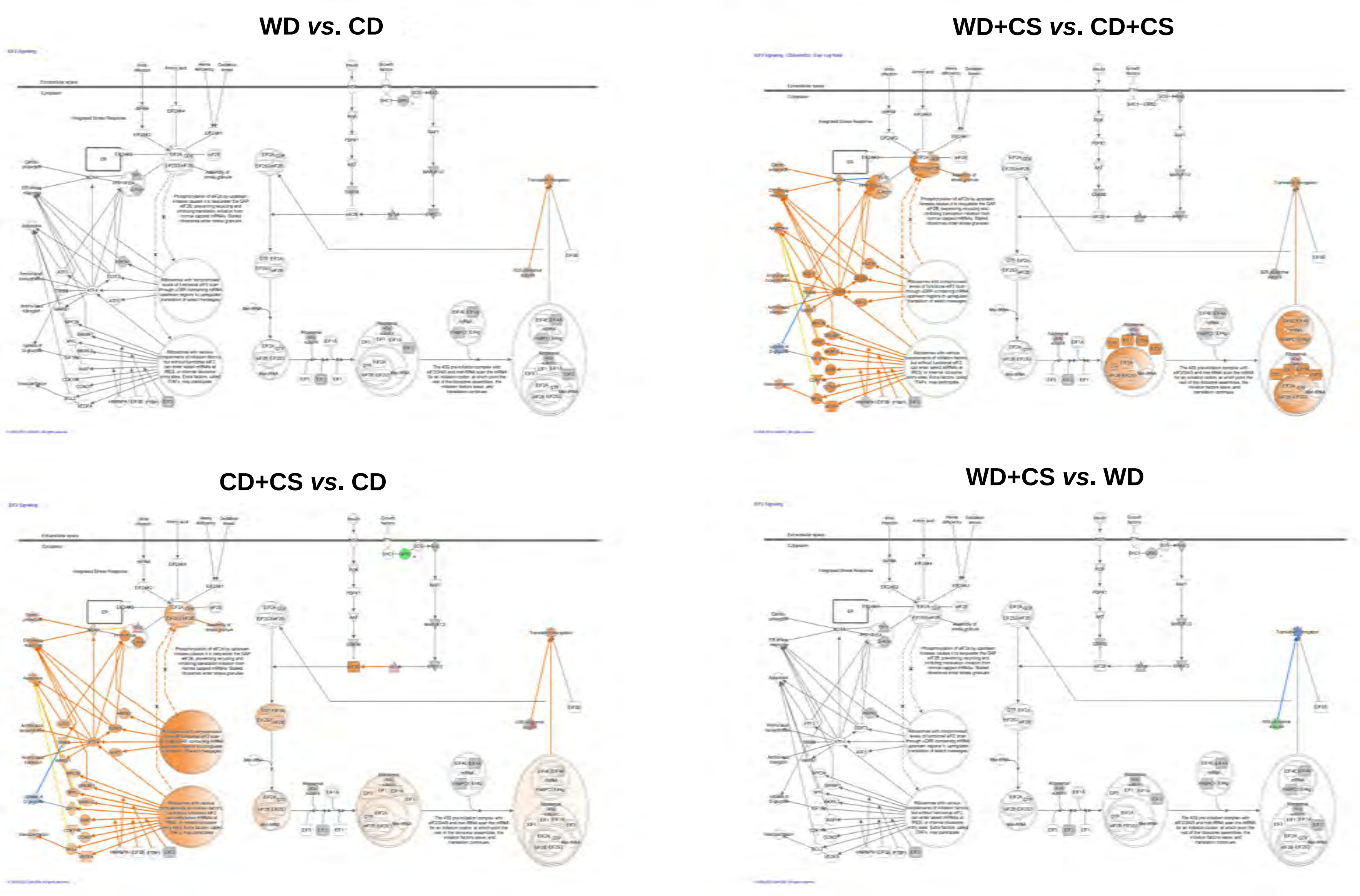

Fig. S16. Mitochondrial Dysfunction Pathway Response in HPC

WD *vs*. CD

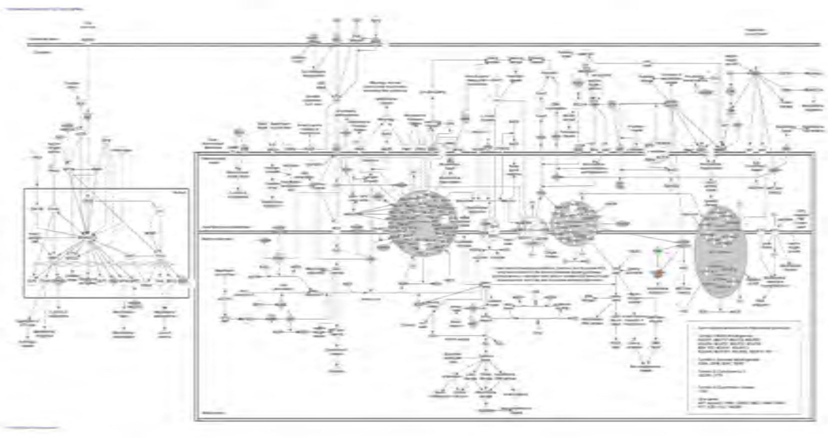

WD+CS *vs*. CD+CS

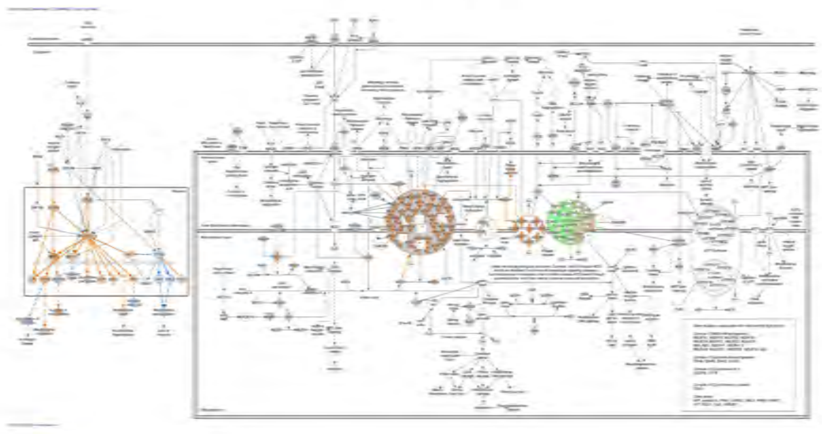

CD+CS *vs*. CD

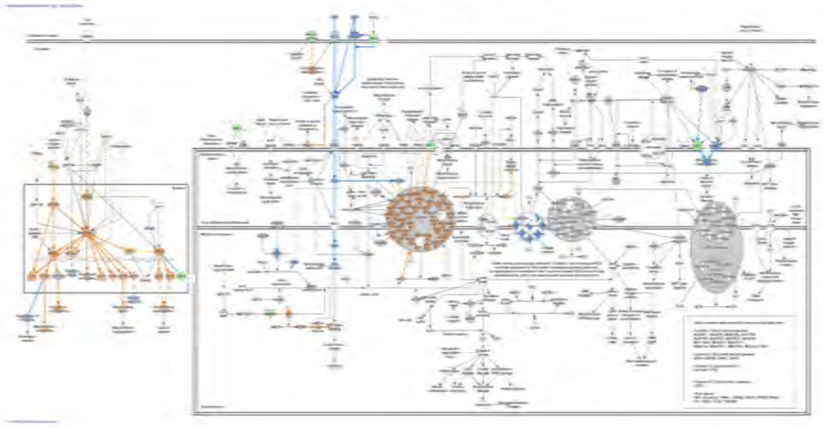

WD+CS *vs*. WD

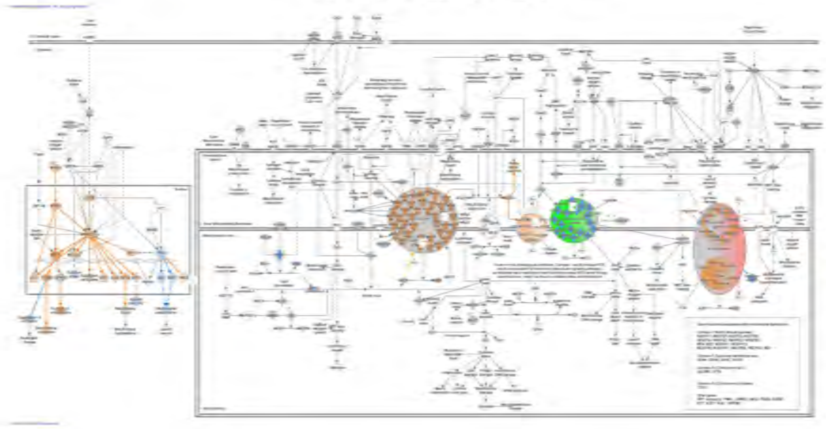

Fig. S17. Chaperone-Mediated Autophagy Pathway Response in HPC Tissue

WD *vs.* CD

WD+CS *vs.* CD+CS

CD+CS *vs.* CD

WD+CS *vs.* WD

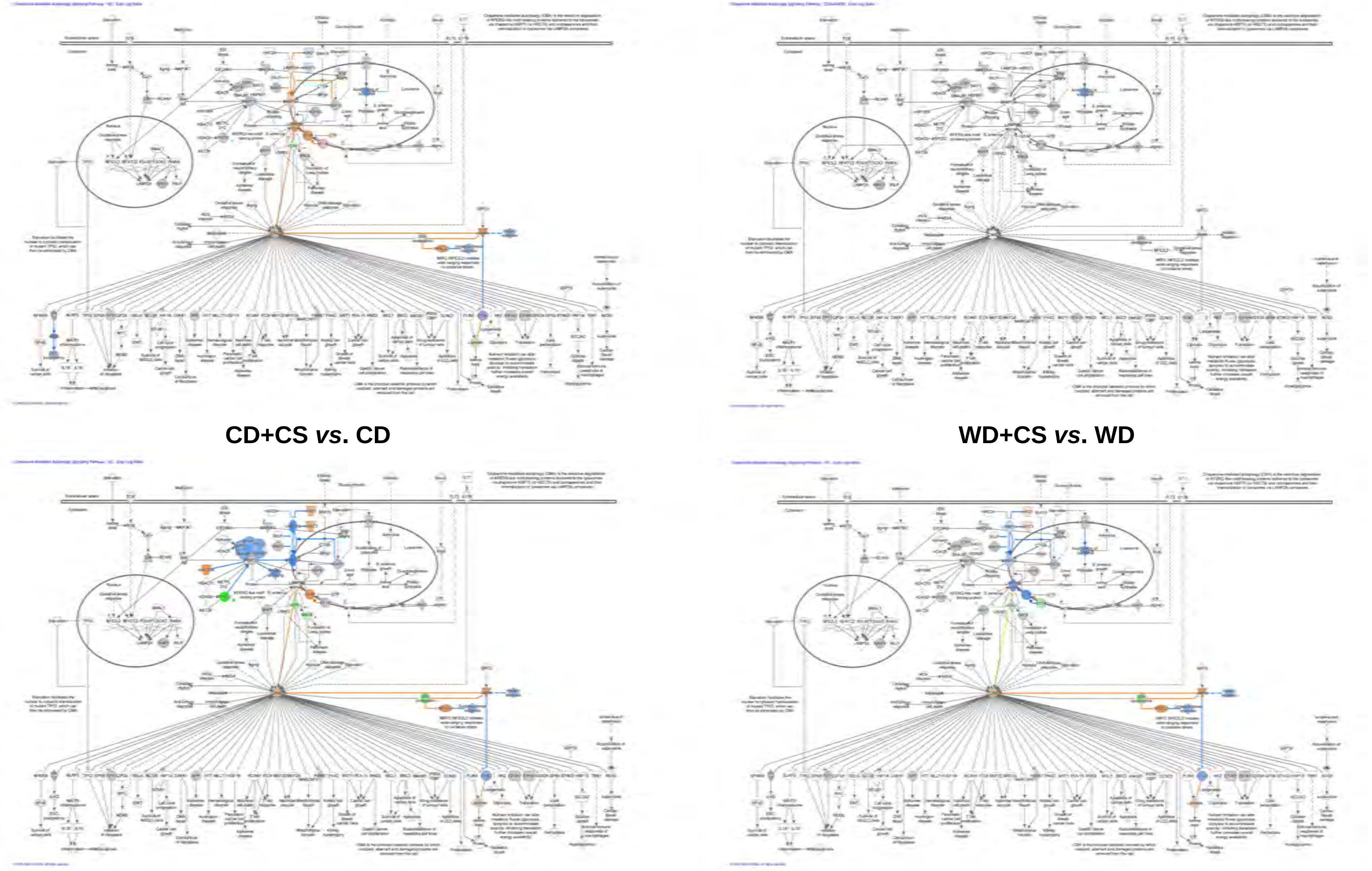

Fig. S18. Synaptogenesis Pathway Responses in HPC Tissue

WD *vs*. CD

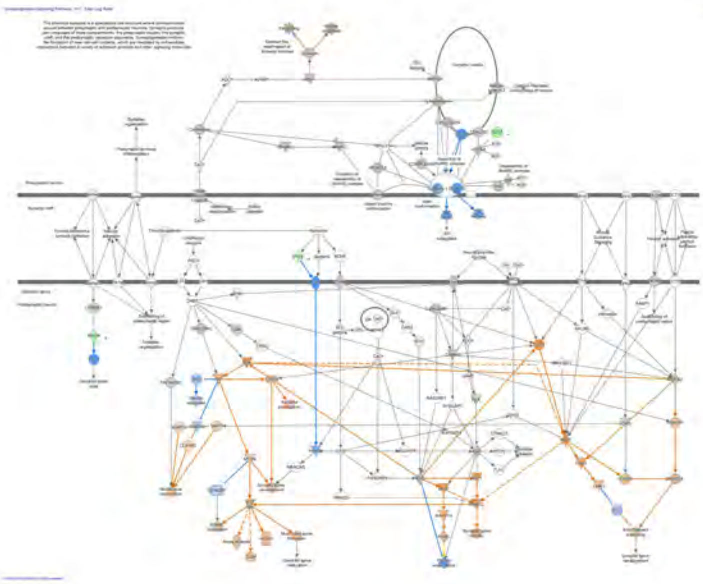

WD+CS *vs*. CD+CS

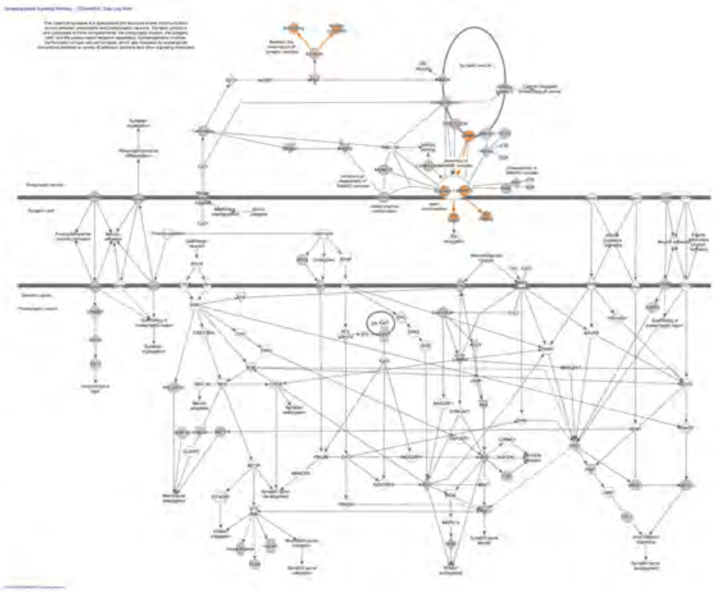

CD+CS *vs*. CD

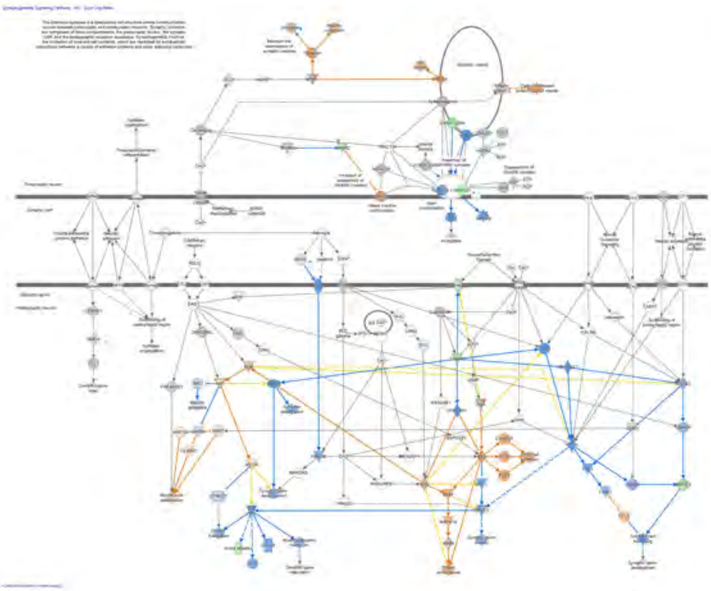

WD+CS *vs*. WD

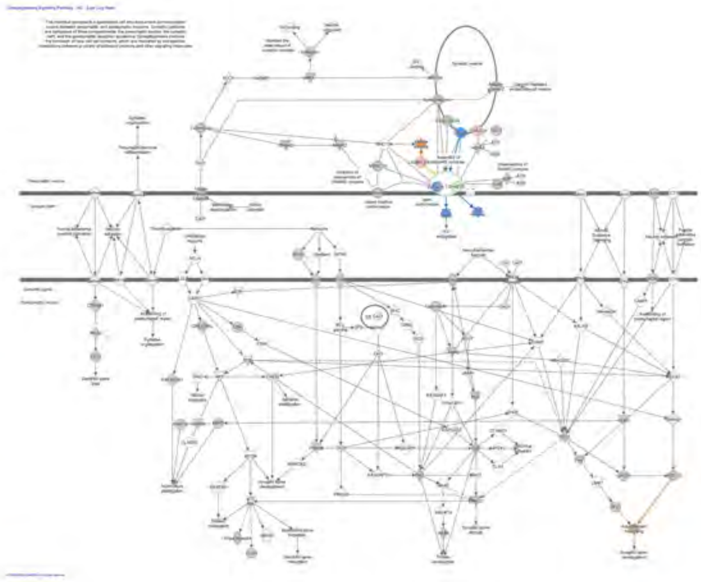

Fig. S19. Reelin Signaling Pathway Responses in HPC Tissue

WD *vs*. CD

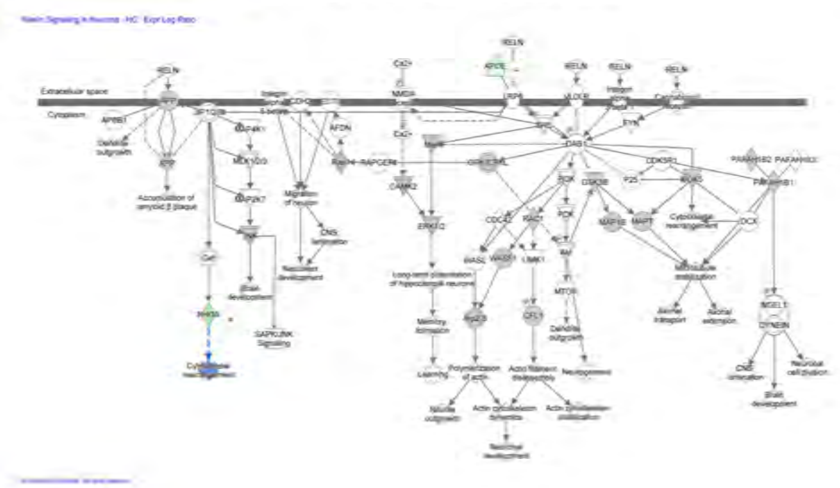

WD+CS *vs*. CD+CS

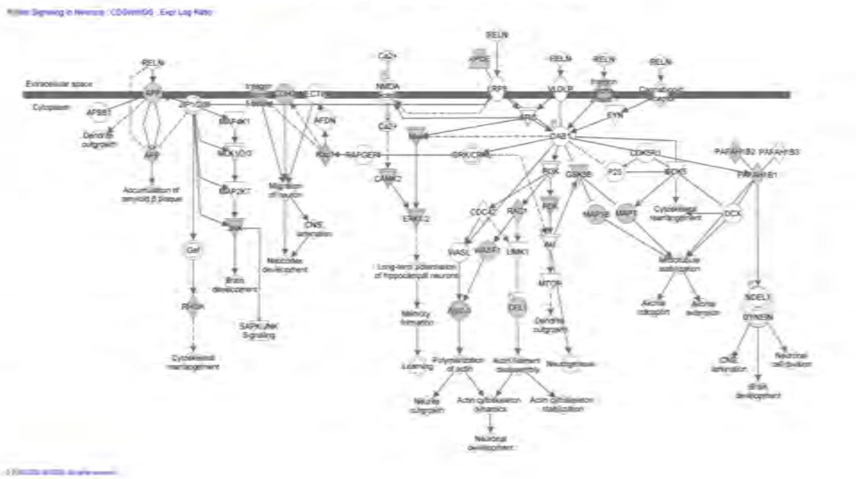

CD+CS *vs*. CD

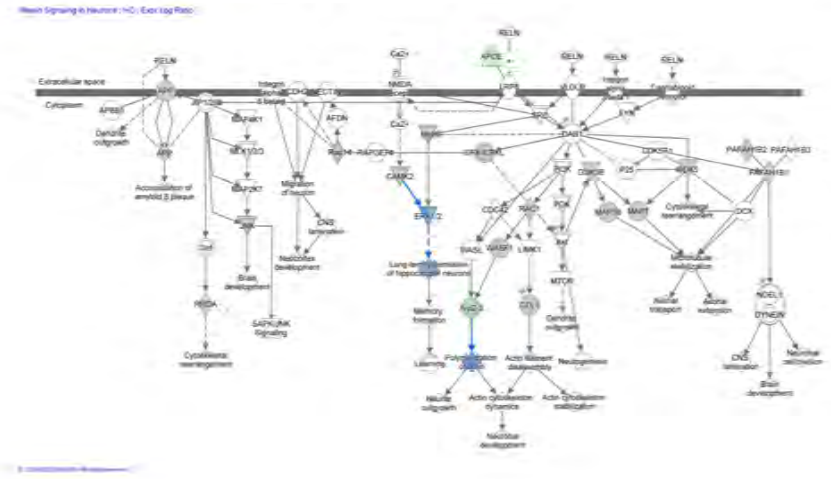

WD+CS *vs*. WD

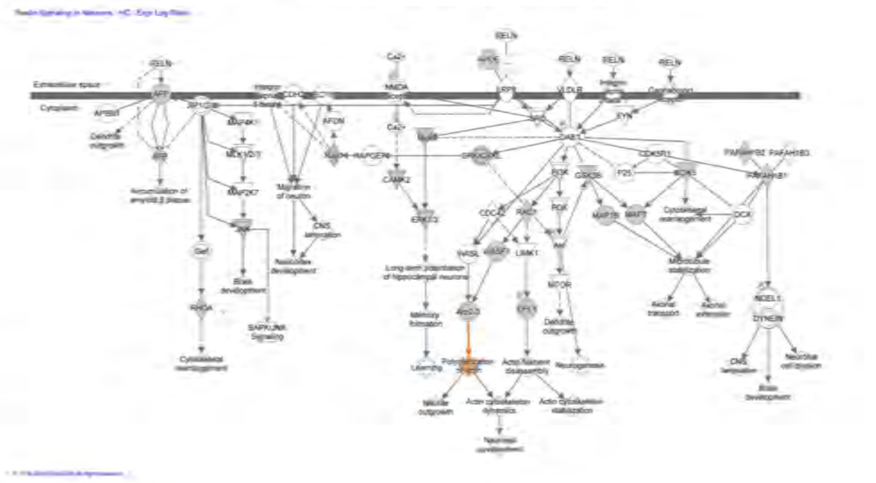

Fig. S20. NRF2 Pathway Response in HPC Tissue

WD *vs.* CD

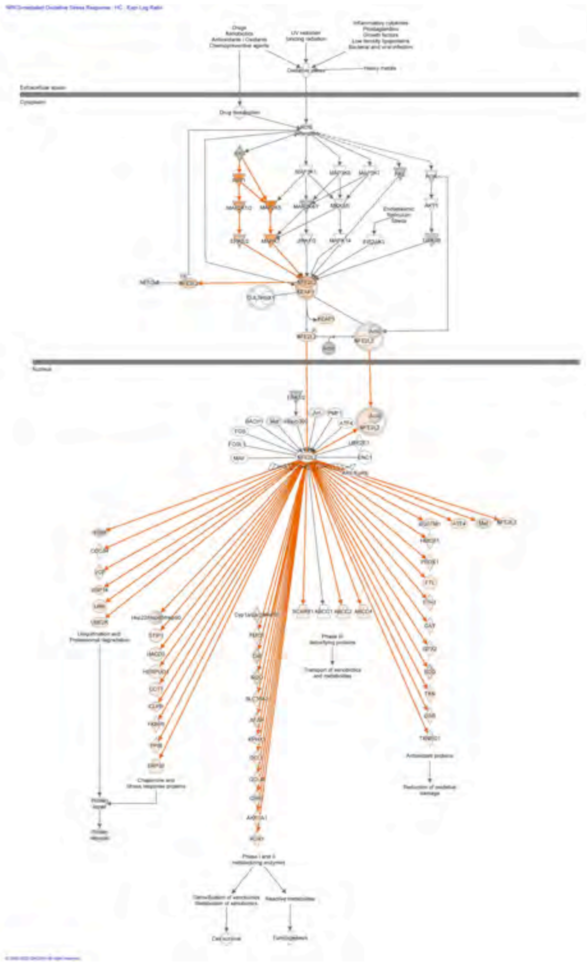

WD+CS *vs.* CD+CS

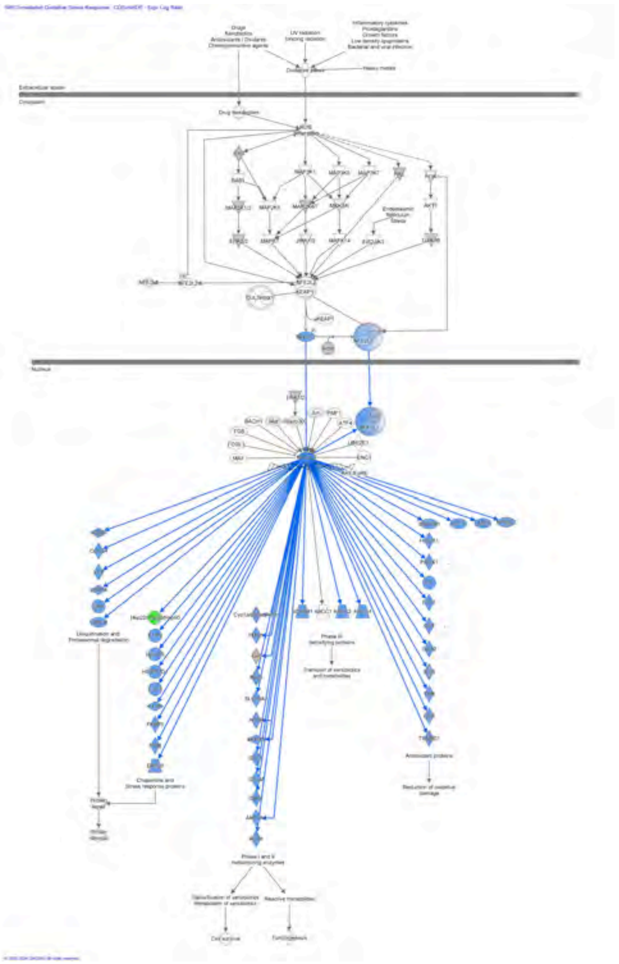

CD+CS *vs.* CD

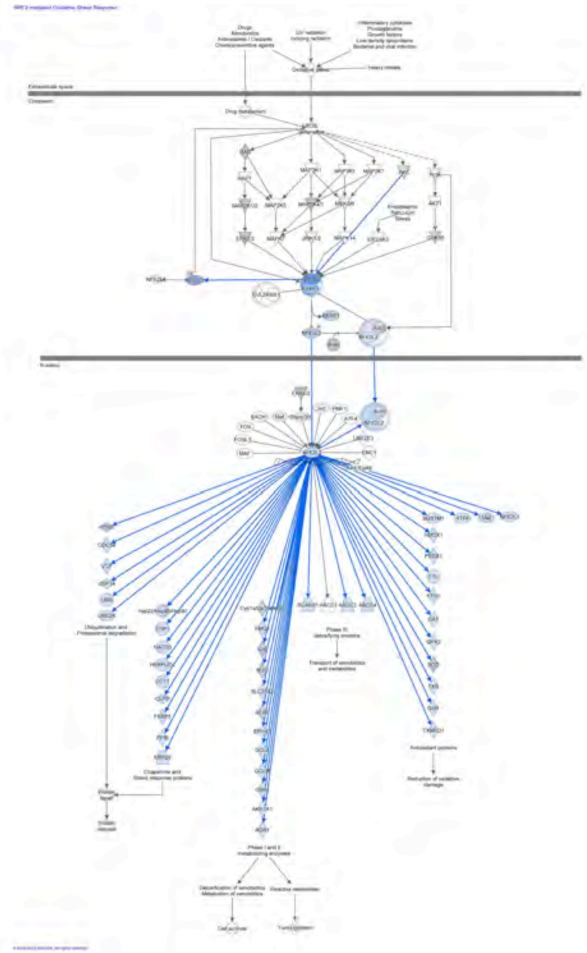

WD+CS *vs.* WD

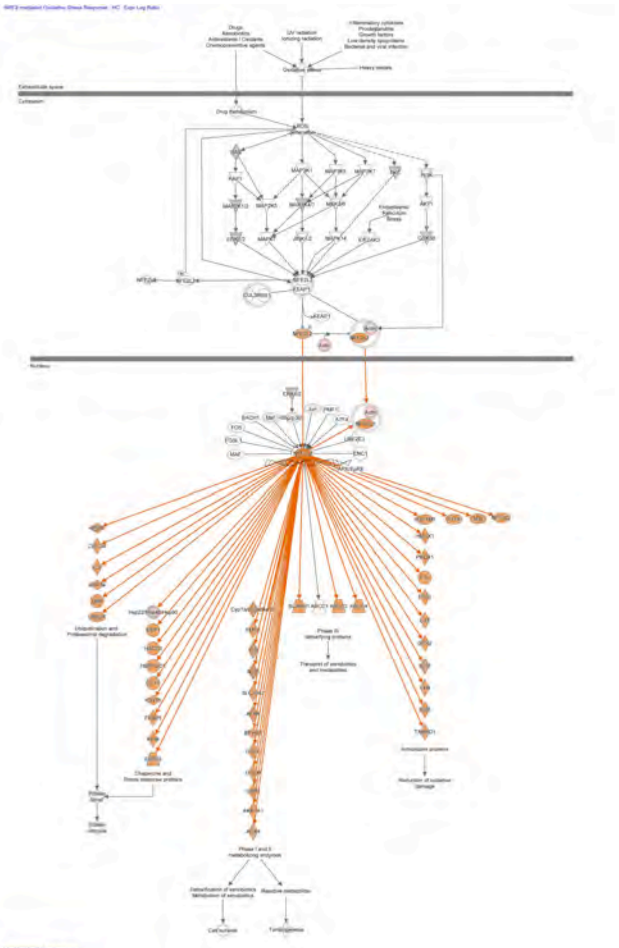

Fig. S21. EIF2 Pathway Response in HPC Tissue

WD *vs.* CD

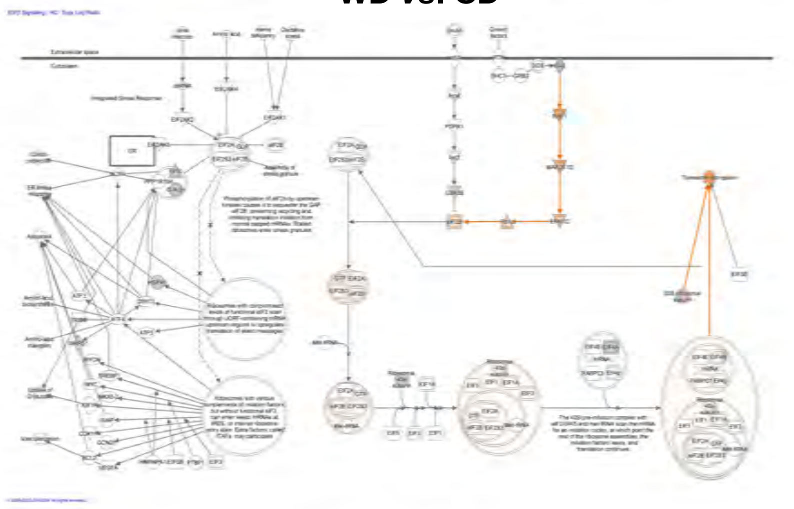

WD+CS *vs.* CD+CS

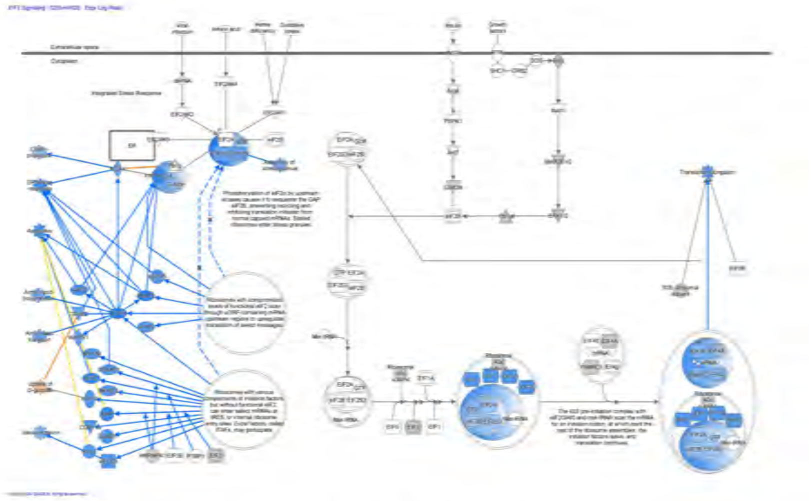

CD+CS *vs.* CD

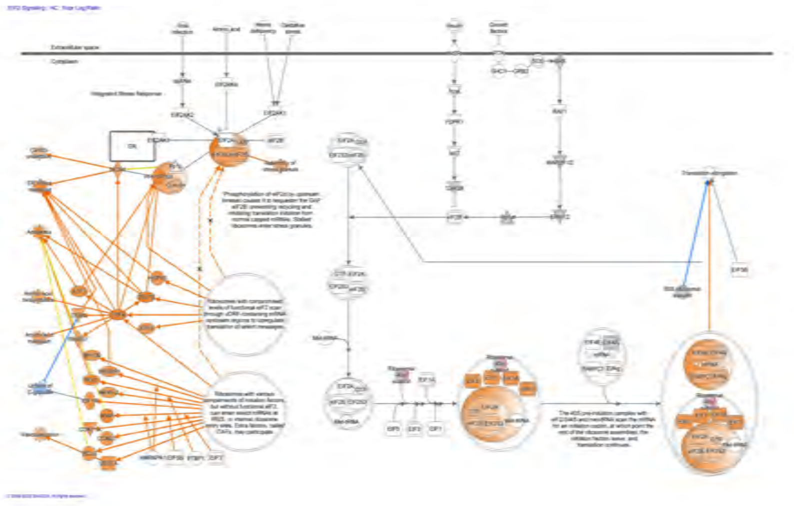

WD+CS *vs.* WD

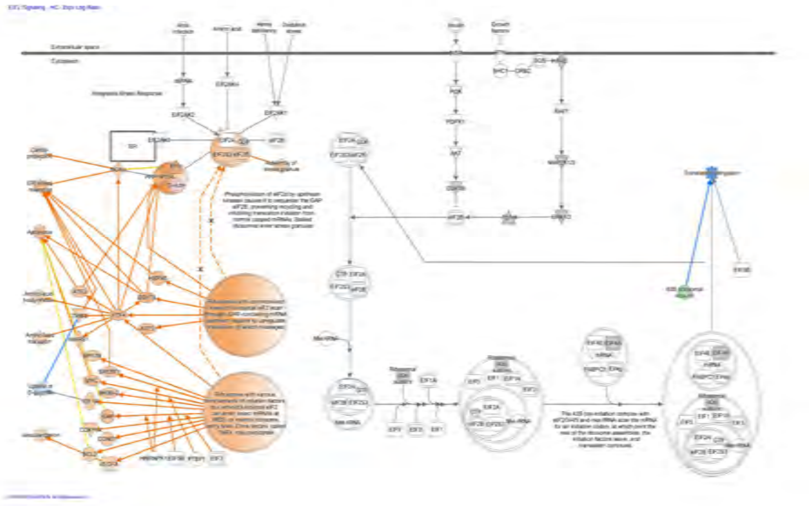

Supplement: Supplementary file 1 — Supplementary file1 (PDF 3460 KB) [file 11011_2026_1855_MOESM1_ESM.pdf]
